# Supplementary material for: Prediction models for post-discharge mortality among under-five children with suspected sepsis in Uganda: A multicohort analysis
Source: PLOS Glob Public Health. 2024 Apr 29;4(4):e0003050. doi: 10.1371/journal.pgph.0003050 (PMC11057737; doi:10.1371/journal.pgph.0003050)
Supplement: S1 Text — (DOCX) [file pgph.0003050.s002.docx]

Prediction models for post-discharge mortality among under-five children with suspected sepsis in Uganda: A multicohort analysis

**Supplementary Material S1 Text**

Contents

[S1: Details of study cohorts and variables used for the full and intermediary models 2](#_Toc163370872)

[Recruitment details, timelines and sites 2](#_Toc163370873)

[**Table A.** Study enrolment sites 2](#_Toc163370874)

[**Table B.** Candidate variables used in the full prediction model, intermediary clinical variable model, and the intermediary clinical and social variable model for both **0-6-month** and **6-60-month** cohorts. 3](#_Toc163370875)

[**Table C.** List of candidate variables used in the full prediction model, intermediary clinical variable model, and the intermediary clinical and social variable model for only the **0-6-month** cohort 4](#_Toc163370876)

[**Table D.** Diagnoses at discharge 4](#_Toc163370877)

[**Figure A.** Post-discharge mortality by age cohort. Both the derivation and validation cohorts were combined for each of the age cohorts. 5](#_Toc163370878)

# S1: Details of study cohorts and variables used for the full and intermediary models

## Recruitment details, timelines and sites

The overall cohort of children 6-60 months of age consisted of a previously collected and reported dataset of 1307 children, of whom 1242 were discharged alive,^1^ and data collected for the present study between July 2017 and July 2020;^2^ both studies used identical eligibility criteria. The main cohort of children 0-6 months of age were recruited between January 2018 and March 2020, along with an additional cohort enrolled between March 2020 and August 2021. All children were followed up for 6 months after hospital discharge.

Children who resided outside of the hospital catchment, or were admitted for a brief period of observation, were excluded. Children admitted immediately after birth (i.e., without having first been discharged) were also excluded from the 0-6-month cohort.

All study materials are available in the Smart Discharges study Dataverse, including: the study protocol and data collection tools;^3^ and the data, analysis code, and data dictionary.^4^

1 Wiens MO, Kumbakumba E, Larson CP, et al. Postdischarge mortality in children with acute infectious diseases: derivation of postdischarge mortality prediction models. BMJ Open 2015; 5: e009449.

2 Wiens MO, Bone JN, Kumbakumba E, et al. Mortality after hospital discharge among children younger than 5 years admitted with suspected sepsis in Uganda: a prospective, multisite, observational cohort study. Lancet Child Adolesc Heal 2023; : DOI: 10.1016/S2352-4642(23)00052-4.

3 Wiens M, Kissoon N (Tex), Ansermino JM, et al. Smart Discharges to improve post-discharge health outcomes in children: A prospective before-after study with staggered implementation. Borealis, V1. 2023. https://doi.org/10.5683/SP3/QRUMNQ (accessed May 2, 2023).

4 Wiens MO, Bone JN, Kumbakumba E, et al. Post-discharge mortality among children under 5 years admitted with suspected sepsis in Uganda: a prospective multi-site study ~ Smart Discharges. Borealis, V2. 2022. https://doi.org/10.5683/SP3/REPMSY (accessed Dec 19, 2022).

## **Table A.** Study enrolment sites

| **Enrolment period** | **2012 – 2013** | **2017-2019** | **2018-2020** | **2020 – 2021** |
| --- | --- | --- | --- | --- |
| **Cohort** | **6-60-month** | **6-60-month** | **0-6-month** | **0-6-month** |
| **Mbarara Regional Referral Hospital**  (Southwestern Uganda) | ✓ | ✓ | ✓ | ✓ |
| **Holy Innocents Children’s Hospital**  (Southwestern Uganda) | ✓ | ✓ | ✓ | ✓ |
| **Masaka Regional Referral Hospital**  (Central Uganda) |  | ✓ | ✓ | ✓ |
| **Jinja Regional Referral Hospital**  (Eastern Uganda) |  | ✓ | ✓ | ✓ |
| **Villa Maria Hospital**  (Central Uganda) |  |  | ✓ | ✓ |
| **Uganda Martyrs Hospital, Ibanda**  (Southwestern Uganda) |  |  | ✓ | ✓ |

## **Table B.** Candidate variables used in the full prediction model, intermediary clinical variable model, and the intermediary clinical and social variable model for both **0-6-month** and **6-60-month** cohorts.

See [Table C](#_Table_S1.3_List) for additional variables used only in the 0-6-month cohort.

| **Variable** | **Full Model** | **Intermediary Clinical Variable Model** | **Intermediary Clinical and Social Variable Model** |
| --- | --- | --- | --- |
| Sex, binary | ✓ | ✓ | ✓ |
| Age, months | ✓ | ✓ | ✓ |
| BMI Z-score | ✓ |  |  |
| MUAC, mm | ✓ | ✓ | ✓ |
| Weight for age Z-score | ✓ | ✓ | ✓ |
| Weight for length Z-score | ✓ |  |  |
| How long since last admission, categorical | ✓ | ✓ | ✓ |
| SpO_2,_ % | ✓ | ✓ | ✓ |
| SpO_2_ transformed | ✓ | ✓ | ✓ |
| Heart rate, beats per minute | ✓ |  |  |
| Respiratory rate, breaths per minute | ✓ | ✓ | ✓ |
| Systolic blood pressure, mmHg | ✓ |  |  |
| Diastolic blood pressure, mmHg | ✓ |  |  |
| Temperature, °C | ✓ | ✓ | ✓ |
| Temperature-squared | ✓ | ✓ | ✓ |
| Abnormal BCS, binary | ✓ | ✓ | ✓ |
| Malaria test positive, binary | ✓ | ✓ | ✓ |
| HIV+, binary | ✓ | ✓ | ✓ |
| Haemoglobin, g/dl | ✓ |  |  |
| Time to reach hospital, categorical | ✓ |  | ✓ |
| Maternal age, years | ✓ |  | ✓ |
| Number of children | ✓ |  | ✓ |
| Had a child who died previously, binary | ✓ |  | ✓ |
| Maternal education, categorical | ✓ |  | ✓ |
| Maternal HIV, categorical | ✓ |  | ✓ |
| Bed net use, categorical | ✓ |  | ✓ |
| Water source, categorical | ✓ |  | ✓ |
| Boil/disinfect/filter water, binary | ✓ |  | ✓ |

Abbreviations: BCS = Blantyre coma scale; HIV = human immunodeficiency virus; MUAC = mid-upper arm circumference; SpO_2_ = oxygen saturation

## **Table C.** List of candidate variables used in the full prediction model, intermediary clinical variable model, and the intermediary clinical and social variable model for only the **0-6-month** cohort

| **Variable** | **Full Model** | **Intermediary Clinical Variable Model** | **Intermediary Clinical and Social Variable Model** |
| --- | --- | --- | --- |
| Abdominal distension, binary | ✓ | ✓ | ✓ |
| Number of antenatal visits | ✓ |  | ✓ |
| Dehydration, WHO categories | ✓ | ✓ | ✓ |
| Delivery method, binary | ✓ |  | ✓ |
| Duration of present illness, categorical | ✓ | ✓ | ✓ |
| Fontenelle, binary | ✓ | ✓ | ✓ |
| Glucose, mmol/L | ✓ |  |  |
| Not previously tested for HIV, binary | ✓ |  |  |
| Referral visit, binary | ✓ | ✓ | ✓ |
| Neonatal jaundice, binary | ✓ | ✓ | ✓ |
| Lactate, mmol/L | ✓ |  |  |
| Mother currently acutely ill, binary | ✓ |  | ✓ |
| Mother has chronic illness, binary | ✓ |  | ✓ |
| Child less than 30 days old, binary | ✓ | ✓ | ✓ |
| Pallor, binary | ✓ | ✓ | ✓ |
| Premature birth, binary | ✓ | ✓ | ✓ |
| Prior care sought for current illness, binary | ✓ | ✓ | ✓ |
| Sucking well when breastfeeding, or feeding well if not breastfed, binary | ✓ | ✓ | ✓ |
| Sucking well when breastfeeding, or feeding well if not breastfed, prior to illness, binary | ✓ | ✓ | ✓ |
| When did baby cry after birth, categorical | ✓ |  |  |
| Abnormal tone, binary | ✓ | ✓ | ✓ |
| Decreased urine production, binary | ✓ | ✓ | ✓ |

Abbreviations: HIV = human immunodeficiency virus; WHO = World Health Organization

## **Table D.** Diagnoses at discharge

|  | **0-6-month cohort, n (%)**  **N=3,349** | **6-60-month cohort, n (%)**  **N=4,830** |
| --- | --- | --- |
| Bronchiolitis | 233 (7%) | 158 (3.3%) |
| Diarrhoea/gastroenteritis | 262 (7.8%) | 842 (17.4%) |
| Febrile convulsions | 6 (0.2%) | 23 (0.5%) |
| HIV or HIV related illness | 20 (0.6%) | 98 (2%) |
| Malaria | 287 (8.6%) | 1711 (35.4%) |
| Malnutrition | 131 (3.9%) | 393 (8.1%) |
| Measles | 56 (1.7%) | 376 (7.8%) |
| Meningitis/encephalitis | 155 (4.6%) | 118 (2.4%) |
| Pneumonia | 1130 (33.7%) | 1489 (30.8%) |
| Reactive airway disease/asthma | 6 (0.2%) | 33 (0.7%) |
| Respiratory tract infection (cold, flu, etc.) | 131 (3.9%) | 420 (8.7%) |
| Sepsis | 1479 (44.2%) | 818 (16.9%) |
| Skin or soft tissue infection | 166 (5%) | 68 (1.4%) |
| Tuberculosis | 17 (0.5%) | 86 (1.8%) |
| Other infectious disease | 61 (1.8%) | 119 (2.5%) |
| Other non-infectious disease | 319 (9.5%) | 202 (4.2%) |


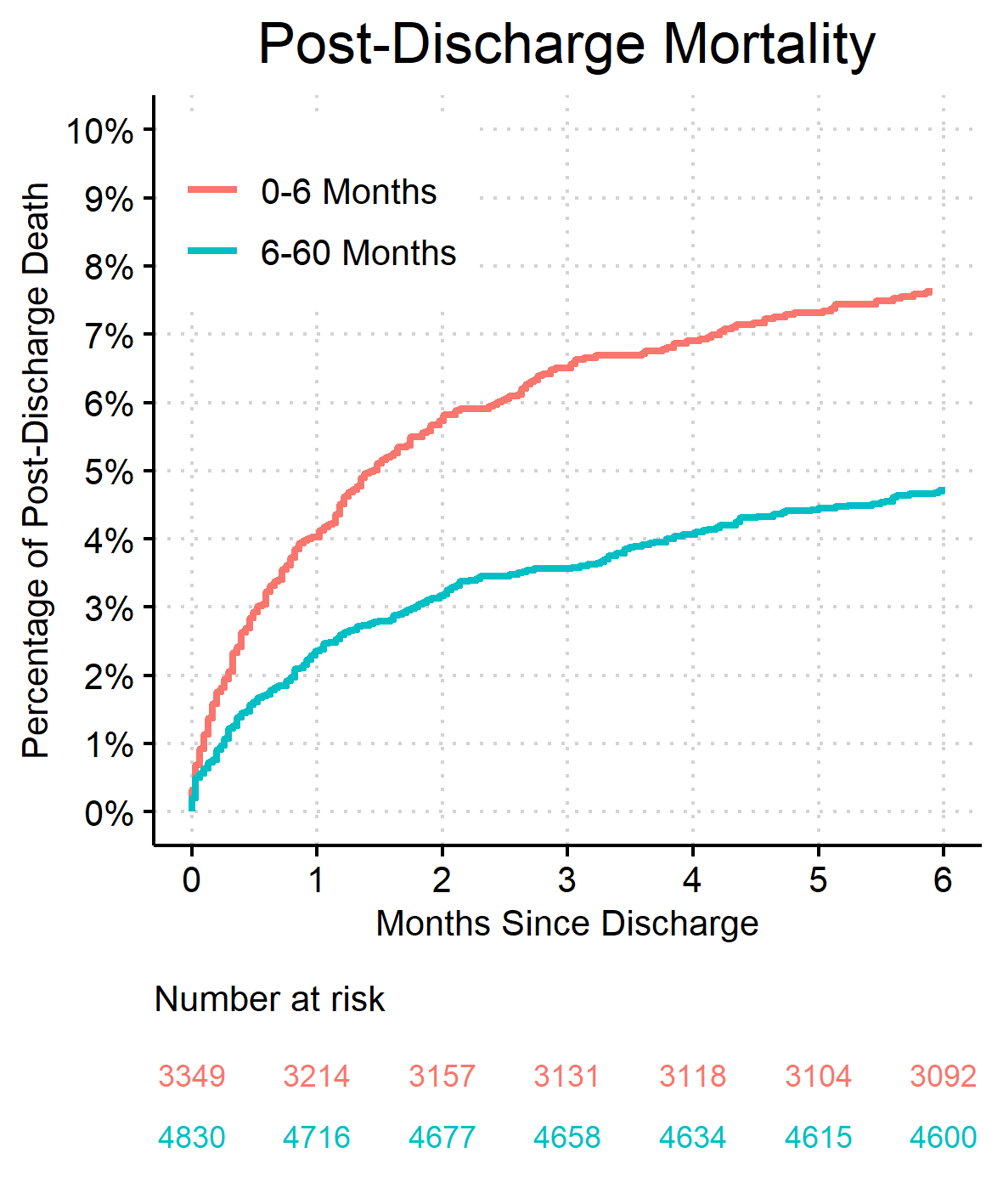


## **Figure A.** Post-discharge mortality by age cohort. Both the derivation and validation cohorts were combined for each of the age cohorts.
