# Supplementary material for: Prediction models for post-discharge mortality among under-five children with suspected sepsis in Uganda: A multicohort analysis
Source: PLOS Glob Public Health. 2024 Apr 29;4(4):e0003050. doi: 10.1371/journal.pgph.0003050 (PMC11057737; doi:10.1371/journal.pgph.0003050)
Supplement: S2 Text — (DOCX) [file pgph.0003050.s003.docx]

Prediction models for post-discharge mortality among under-five children with suspected sepsis in Uganda: A multicohort analysis

**Supplementary Material S2**

Contents

[S2: Statistical Methods and Analyses 2](#_Toc163371321)

[Adjustment and transformation of candidate predictors 2](#_Toc163371322)

[Sample size 2](#_Toc163371323)

[Learning Curve of Training Sample Size vs Performance 3](#_Toc163371324)

[**Figure A.** Learning curve showing the performance in terms of area under the curve (AUC) and 95% confidence intervals of the elastic net derivation model against the training sample size. 3](#_Toc163371325)

[Statistical methods applied during model development 3](#_Toc163371326)

[References for Statistical Methods 4](#_Toc163371327)

# S2: Statistical Methods and Analyses

## Adjustment and transformation of candidate predictors

All continuous variables were centred and scaled, and categorical variables converted to indicators (i.e. dummy variables). In addition to the raw oxygen saturation (SpO_2_) measurement, we used the transformation for SpO_2_ proposed by Zhou *et al.* to improve model prediction and calibration.^5^ Z-scored variables, including body mass index (BMI) z-scores, weight-for-age z-scores, and weight-for-length z-scores were calculated according to the World Health Organization (WHO) Child Growth Standards.^6^ Dehydration in the 0-6-month age group was determined using the WHO assessment criteria for dehydration.^7^ We included a quadratic term for temperature since both high and low temperatures may increase risk. Other nonlinearities were not considered due to risk of overfitting with the limited number of available events per variable.

## Sample size

The sample size for the primary study enrolment was determined to accomplish three primary aims. First, to explore the epidemiology of post-discharge mortality, which has been previously reported.^2^ Second, to develop prediction models. Third to act as a control period for a later interventional phase.

For the present analysis, we determined the sample size required to develop a prediction model based on criteria proposed by Riley *et al*., 2020.^8^ For binary outcomes, three criteria are recommended based on: 1) reducing overfitting (caused by small sample sizes or too many candidate predictors relative to the sample size or number of events), defined by an expected shrinkage of predictor effects by ≤10%; 2) a small absolute difference of 0.05 in the apparent and adjusted Nagelkerke’s R^2^ value of the model, whereby the apparent R^2^ reflects the model performance in the same way that was used to develop the model and the adjusted R^2^ is an approximately unbiased estimate of the model fit;^9^ and 3) estimating the outcome proportion to within ±5% precision. The estimated sample size required to satisfy the three criteria was 2,117 and 1,551 for the 0-6-month and 6-60-month cohorts, respectively.

We also created *post hoc* learning curves of the sample size used to develop the model versus the area under the receiver operating characteristic curve (AUROC) when tested against a 20% hold-out set using the full set of variable predictors (**Figure A in S2 Text**).^10^ This involved building models using an increasing subset of the population (up to 80% of the total sample) and evaluating their performance against the hold-out set. For the 6-60-month learning curve, we started with the initial 1,242 children from 2012-2014,^1^ followed by the first 258 children recruited starting from July 2017 (n=1,500) and thereafter added groups of 500 consecutively recruited children until 80% (n=3,864) of the available population was reached. For the 0-6-month learning curve, we started with the first 500 children recruited starting from January 2018 and continued to add groups of 500 consecutively recruited children until 80% (n=2,679) of the available population was reached.

The performance of the 6-60-month derivation model increased with increasing sample size up to approximately 2,500 children after which the AUROC stabilized. The low performance of the model using only the initial 1,242 children may indicate model drift. The AUROC for the 0-6-month derivation model stabilized at approximately 1,000 children. This suggests that increasing our sample sizes beyond what we have currently collected would not result in further improvement of model performance.

## Learning Curve of Training Sample Size vs Performance


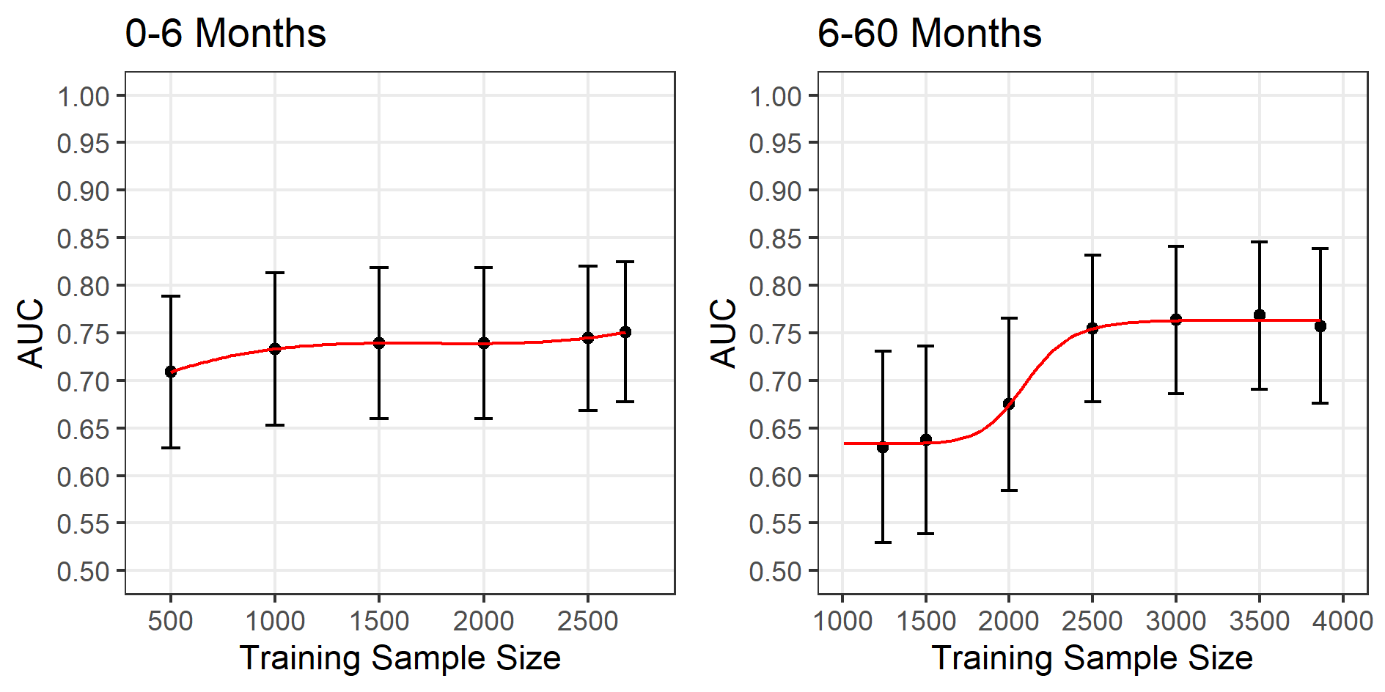


## **Figure A.** Learning curve showing the performance in terms of area under the curve (AUC) and 95% confidence intervals of the elastic net derivation model against the training sample size.

*Performance was evaluated using the last 20% of children that were recruited for each age cohort. The training samples were increased based on chronological order of recruitment, i.e., for the* ***0-6-month*** *cohort, the first 500 patients recruited in the study were used to create the model and its performance tested on the 20% holdout dataset. This was repeated sequentially until the remaining 80% of the cohort was used. The training sample for the* ***6-60-month*** *cohort started with the initial 1,242 patients recruited in 2012-2013.*

## Statistical methods applied during model development

All candidate predictors were summarised with means and standard deviations for continuous variables and counts and percentages for categorical variables. As the amount of missing data overall and on any individual predictor was low, we used single-imputation with K-nearest neighbours imputation to replace missing values.^11^ Multiple imputation was considered, but not used, as this leads to additional complexity in the model building and validation steps.^12^

We developed full variable, intermediary, and final models utilizing the subset of variables as described in the Model Development section of the main paper. Elastic net regression was used to estimate coefficients for the prediction model.^13^ In sample sizes similar to ours, elastic net has been shown to perform similarly to more data-driven machine learning algorithms.^14,15^

We conducted 10-fold nested cross-validation to optimise the tuning parameters of the elastic net and provide a final evaluation of model performance. Briefly, this process consists of an outer loop and an inner loop. In the outer loop, the entire dataset was split into 10 folds, each one consisting of 90% of the original sample (training set) while the remaining 10% was used as a test set for final evaluation. Within each training set of the outer loop, an internal 10-fold cross-validation was conducted in which the training set was split into 10 folds (inner loop). The inner loop was used to select optimal tuning parameters terms for the elastic net across a pre-specified grid of possible values. The model developed within each training set of the outer loop was then evaluated on the withheld test set.^16^ For each fold in the outer loop, we estimated the AUROC, the specificity, positive predictive value, and negative predictive value, using the probability threshold that gives 80% sensitivity, area under the precision recall curve (PRAUC), and Brier score. Internal model performance was assessed based on the cross-validated mean of the selected performance metrics. These performance metrics were also calculated on the entire dataset without cross-validation. We included additional plots for the gain curve, calibration, and the distribution of predicted probabilities stratified by mortality.

All analyses were conducted using R statistical software version 4.2.2 (R Foundation for Statistical Computing, Vienna, Austria) with the *caret* package (version 6.0-93) for model building and validation.^17,18^

## References for Statistical Methods

5 Zhou G, Karlen W, Brant R, Wiens M, Kissoon N, Ansermino JM. A transformation of oxygen saturation (the saturation virtual shunt) to improve clinical prediction model calibration and interpretation. Pediatr Res 2019; 86: 732–7.

6 World Health Organization. Child growth standards. 2023. http://www.who.int/childgrowth/standards/en/ (accessed March 27, 2023).

7 World Health Organization. The treatment of diarrhoea: a manual for physicians and other senior health workers, 4th rev. 2005. https://apps.who.int/iris/handle/10665/43209 (accessed March 27, 2023).

8 Riley RD, Ensor J, Snell KIE, et al. Calculating the sample size required for developing a clinical prediction model. BMJ 2020; 368: m441.

9 Nagelkerke NJD. A Note on a General Definition of the Coefficient of Determination. Biometrika 1991; 78: 691.

10 Balki I, Amirabadi A, Levman J, et al. Sample-Size Determination Methodologies for Machine Learning in Medical Imaging Research: A Systematic Review. Can Assoc Radiol J 2019; 70: 344–53.

11 Andridge RR, Little RJA. A Review of Hot Deck Imputation for Survey Non-response. Int Stat Rev 2010; 78: 40–64.

12 Mandel J SP. A Comparison of Six Methods for Missing Data Imputation. J Biom Biostat 2015; 06: 1000224.

13 Zou H, Hastie T. Regularization and variable selection via the elastic net. J R Stat Soc Ser B (Statistical Methodol 2005; 67: 301–20.

14 Sanchez-Pinto LN, Venable LR, Fahrenbach J, Churpek MM. Comparison of variable selection methods for clinical predictive modeling. Int J Med Inform 2018; 116: 10–7.

15 van der Ploeg T, Austin PC, Steyerberg EW. Modern modelling techniques are data hungry: a simulation study for predicting dichotomous endpoints. BMC Med Res Methodol 2014; 14: 137.

16 Steyerberg EW, Harrell FE, Borsboom GJJ., Eijkemans MJ., Vergouwe Y, Habbema JDF. Internal validation of predictive models: Efficiency of some procedures for logistic regression analysis. J Clin Epidemiol 2001; 54: 774–81.

17 R Core Team. R: A language and environment for statistical computing. R Founcation Stat. Comput. Vienna, Austria. 2021. https://www.r-project.org/ (accessed April 10, 2022).

18 Kuhn M. Building Predictive Models in R Using the caret Package. J Stat Softw 2008; 28. DOI:10.18637/jss.v028.i05.
