# Supplementary material for: Prediction models for post-discharge mortality among under-five children with suspected sepsis in Uganda: A multicohort analysis
Source: PLOS Glob Public Health. 2024 Apr 29;4(4):e0003050. doi: 10.1371/journal.pgph.0003050 (PMC11057737; doi:10.1371/journal.pgph.0003050)
Supplement: S3 Text — (DOCX) [file pgph.0003050.s004.docx]

Prediction models for post-discharge mortality among under-five children with suspected sepsis in Uganda: A multicohort analysis

**Supplementary Material S3**

Contents

[S3: Intermediary Clinical Variable Models – Variable Importance 2](#_Toc163371722)

[**Table A.** Average rank of variable importance and the number of times selected in the top 8 variables across 10 folds of cross-validation from the **0-6-month** intermediary clinical variable model. 2](#_Toc163371723)

[**Table B.** Average rank of variable importance and the number of times selected in the top 8 variables across 10 folds of cross-validation from the **6-60-month** intermediary clinical variable model. 3](#_Toc163371724)

# S3: Intermediary Clinical Variable Models – Variable Importance

## **Table A.** Average rank of variable importance and the number of times selected in the top 8 variables across 10 folds of cross-validation from the **0-6-month** intermediary clinical variable model.

Only the top 20 variables and interactions are shown. Interactions between variables are indicated by the multiplication sign. The top eight unique variables (highlighted in bold) by average rank were used in the final model including their interactions with age.

| **Variable** | **Average Rank** | **Times Selected in Top 8** |
| --- | --- | --- |
| **Weight for age z-score** | **1.4** | **10** |
| **MUAC** | **1.6** | **10** |
| **Sucking well when breastfeeding, or feeding well if not breastfed** | **3.4** | **10** |
| **SpO_2_** | **5.8** | **9** |
| **Duration of present illness, 8 days – 1 month** | **6.2** | **9** |
| **Age × Jaundice** | **7.8** | **7** |
| **Fontanelle** | **8.3** | **8** |
| Neonate | 8.7 | 5 |
| Prior care sought for current illness | 11.3 | 2 |
| Age × Sucking well when breastfeeding, or feeding well if not breastfed, prior to illness | 11.6 | 5 |
| Malaria | 11.9 | 0 |
| Abnormal tone | 12.5 | 2 |
| Visit referred | 13.0 | 1 |
| Age × How long since last admission, <7 days | 13.6 | 0 |
| Pallor | 16.6 | 0 |
| Abdominal tension | 17.4 | 0 |
| Decreased urine production | 18.7 | 0 |
| How long since last admission, 1 month – 1 years | 19.5 | 0 |
| How long since last admission, 7 days – 1 month | 21.6 | 0 |
| Age × SpO_2_ | 24.7 | 0 |

Abbreviations: MUAC = mid-upper arm circumference; SpO_2_ = oxygen saturation

## **Table B.** Average rank of variable importance and the number of times selected in the top 8 variables across 10 folds of cross-validation from the **6-60-month** intermediary clinical variable model.

Only the top 20 variables and interactions are shown. Interactions between variables are indicated by the multiplication sign. The top nine^1^ unique variables (highlighted in bold) were used in the final model including their interactions with age.

| **Variable** | **Average Rank** | **Times Selected in Top 8** |
| --- | --- | --- |
| **MUAC** | **1.0** | **10** |
| **SpO_2_** | **2.7** | **10** |
| **Weight for age z-score** | **2.8** | **10** |
| **How long since last admission, 7 days – 1 month** | **4.7** | **10** |
| **Abnormal BCS** | **5.1** | **9** |
| **Temperature, °C** | **6.4** | **9** |
| **HIV+** | **6.5** | **9** |
| **Temperature-squared, °C** | **8.0** | **6** |
| **Age × Respiratory rate** | **9.1** | **2** |
| Age × How long since last admission, 1 month – 1 year | 10.5 | 3 |
| How long since last admission, >1 year | 11.3 | 0 |
| How long since last admission, <7 days | 11.4 | 1 |
| Respiratory rate | 13.7 | 1 |
| Age × Abnormal BCS | 16.5 | 0 |
| Age × How long since last admission, <7 days | 17.4 | 0 |
| Age | 18.2 | 0 |
| How long since last admission, 1 month – 1 year | 18.2 | 0 |
| Age × How long since last admission, 7 days – 1 month | 19.4 | 0 |
| Age × HIV+ | 21.4 | 0 |
| Age × MUAC | 21.9 | 0 |

^1^ Nine variables were selected here as the eighth unique variable with the highest importance was within an interaction effect.

Abbreviations: BCS = Blantyre coma scale; HIV = human immunodeficiency virus; MUAC = mid-upper arm circumference; SpO_2_ = oxygen saturation
