# Supplementary material for: Prediction models for post-discharge mortality among under-five children with suspected sepsis in Uganda: A multicohort analysis
Source: PLOS Glob Public Health. 2024 Apr 29;4(4):e0003050. doi: 10.1371/journal.pgph.0003050 (PMC11057737; doi:10.1371/journal.pgph.0003050)
Supplement: S4 Text — (DOCX) [file pgph.0003050.s005.docx]

Prediction models for post-discharge mortality among under-five children with suspected sepsis in Uganda: A multicohort analysis

**Supplementary Material S4**

Contents

[S4: Intermediary Clinical and Social Variable Models – Variable Importance 2](#_Toc163372018)

[**Table A.** Average rank of variable importance and the number of times selected in the top 8 variables across 10 folds of cross-validation from the **0-6-month** intermediary clinical and social variable model. 2](#_Toc163372019)

[**Table B.** Average rank of variable importance and the number of times selected in the top 8 variables across 10 folds of cross-validation from the **6-60-month** intermediary clinical and social variable model. 3](#_Toc163372020)

# S4: Intermediary Clinical and Social Variable Models – Variable Importance

## **Table A.** Average rank of variable importance and the number of times selected in the top 8 variables across 10 folds of cross-validation from the **0-6-month** intermediary clinical and social variable model.

Only the top 20 variables and interactions are shown. Interactions between variables are indicated by the multiplication sign. The top eight unique variables (highlighted in bold) by average rank were used in the final model including their interactions with age.

| **Variable** | **Average Rank** | **Times Selected in Top 8** |
| --- | --- | --- |
| **Weight for age z-score** | **1.1** | **10** |
| **MUAC** | **1.9** | **10** |
| **Time it took to reach hospital, >1 hour** | **3.2** | **10** |
| **Sucking well when breastfeeding, or feeding well if not breastfed** | **4.5** | **10** |
| **SpO_2_** | **6.3** | **8** |
| **Duration of present illness, 8 days – 1 month** | **7.4** | **7** |
| **Age × Neonatal jaundice** | **9.4** | **4** |
| Abnormal tone | 9.7 | 4 |
| Fontanelle | 10.3 | 7 |
| Prior care sought for current illness | 15.0 | 0 |
| Age × How long since last admission, <7 days | 15.0 | 1 |
| Malaria | 15.3 | 1 |
| Neonate | 15.9 | 1 |
| Maternal education | 16.6 | 0 |
| Decreased urine production | 19.7 | 0 |
| Water source, open source | 19.9 | 0 |
| Pallor | 20.2 | 0 |
| Age × Weight for age z-score | 21.0 | 5 |
| How long since last admission, 1 month – 1 year | 21.1 | 0 |
| How long since last admission, 7 days – 1 month | 22.6 | 0 |

Abbreviations: MUAC = mid-upper arm circumference; SpO_2_ = oxygen saturation

## **Table B.** Average rank of variable importance and the number of times selected in the top 8 variables across 10 folds of cross-validation from the **6-60-month** intermediary clinical and social variable model.

Only the top 20 variables and interactions are shown. Interactions between variables are indicated by the multiplication sign. The top eight unique variables (highlighted in bold) by average rank were used in the final model including their interactions with age.

| **Variable** | **Average Rank** | **Times Selected in Top 8** |
| --- | --- | --- |
| **MUAC** | **1.0** | **10** |
| **Weight for age z-score** | **2.5** | **10** |
| **SpO_2_** | **2.8** | **10** |
| **How long since last admission, 7 days – 1 month** | **5.8** | **10** |
| **HIV+** | **5.9** | **8** |
| **Age × Water source, bore hole** | **6.6** | **7** |
| **Boil/disinfect/filter water** | **7.3** | **6** |
| Temperature | 8.3 | 4 |
| Abnormal BCS | 9.0 | 6 |
| Temperature-squared | 10.1 | 4 |
| Age × Time it took to reach hospital, >1 hour | 11.7 | 3 |
| Water source, municipal water | 12.5 | 2 |
| How long since last admission, <7 days | 15.3 | 0 |
| How long since last admission, >1 year | 15.4 | 0 |
| Age × How long since last admission, 1 month – 1 year | 16.5 | 0 |
| Respiratory rate | 16.6 | 0 |
| Age × Water source, municipal water | 17.3 | 0 |
| Malaria | 18.4 | 0 |
| Time it took to reach hospital, >1 hour | 18.4 | 0 |
| Maternal education | 19.0 | 0 |

Abbreviations: HIV = human immunodeficiency virus; MUAC = mid-upper arm circumference; SpO_2_ = oxygen saturation
