# Supplementary material for: Prediction models for post-discharge mortality among under-five children with suspected sepsis in Uganda: A multicohort analysis
Source: PLOS Glob Public Health. 2024 Apr 29;4(4):e0003050. doi: 10.1371/journal.pgph.0003050 (PMC11057737; doi:10.1371/journal.pgph.0003050)
Supplement: S5 Text — (DOCX) [file pgph.0003050.s006.docx]

Prediction models for post-discharge mortality among under-five children with suspected sepsis in Uganda: A multicohort analysis

**Supplementary Material S5**

Contents

[S5: Intermediary Any Variable Models – Performance Metrics, Coefficients and Variable Importance 2](#_Toc163372334)

[**Table A.** Performance metrics across 10 folds of cross-validation from the intermediary **0-6-month** any variable model using the probability threshold that gave 80% sensitivity 2](#_Toc163372335)

[**Table B.** Performance metrics across 10 folds of cross-validation from the intermediary **6-60-month** any variable model using the probability threshold that gave 80% sensitivity 2](#_Toc163372336)

[**Figure A.** Performance of the intermediary **0-6-month** any variable model tested on the entire dataset. 3](#_Toc163372337)

[**Figure B.** Performance of the intermediary **6-60-month** any variable model tested on the entire dataset. 4](#_Toc163372338)

[**Table C.** Coefficients of the intermediary **0-6-month** any variable model 5](#_Toc163372339)

[**Table D.** Coefficients of the intermediary **6-60-month** any variable model. 7](#_Toc163372340)

[**Table E.** Average rank of variable importance and the number of times selected in the top 8 variables across 10 folds of cross-validation from the intermediary **0-6-month** any variable model. 8](#_Toc163372341)

[**Table F.** Average rank of variable importance and the number of times selected in the top 8 variables across 10 folds of cross-validation from the intermediary **6-60-month** any variable model. 9](#_Toc163372342)

# S5: Intermediary Any Variable Models – Performance Metrics, Coefficients and Variable Importance

## **Table A.** Performance metrics across 10 folds of cross-validation from the intermediary **0-6-month** any variable model using the probability threshold that gave 80% sensitivity

| **Fold** | **Specificity** | **Sensitivity** | **AUC** | **PPV** | **NPV** | **PRAUC** | **Brier Score** |
| --- | --- | --- | --- | --- | --- | --- | --- |
| 1 | 0.748 | 0.808 | 0.847 | 0.212 | 0.979 | 0.307 | 0.063 |
| 2 | 0.518 | 0.808 | 0.727 | 0.124 | 0.970 | 0.165 | 0.069 |
| 3 | 0.539 | 0.808 | 0.701 | 0.128 | 0.971 | 0.209 | 0.068 |
| 4 | 0.825 | 0.800 | 0.869 | 0.270 | 0.981 | 0.320 | 0.060 |
| 5 | 0.455 | 0.808 | 0.699 | 0.111 | 0.966 | 0.231 | 0.067 |
| 6 | 0.605 | 0.800 | 0.768 | 0.141 | 0.974 | 0.193 | 0.065 |
| 7 | 0.738 | 0.808 | 0.803 | 0.206 | 0.979 | 0.195 | 0.068 |
| 8 | 0.621 | 0.808 | 0.782 | 0.152 | 0.975 | 0.200 | 0.069 |
| 9 | 0.667 | 0.800 | 0.821 | 0.163 | 0.976 | 0.203 | 0.065 |
| 10 | 0.521 | 0.808 | 0.691 | 0.124 | 0.970 | 0.128 | 0.072 |
| **Average** | **0.624** | **0.805** | **0.771** | **0.163** | **0.974** | **0.215** | **0.067** |

Abbreviations: AUC = area under the receiver operating characteristic curve; PPV = positive predictive value; NPV = negative predictive value; PRAUC = area under the precision-recall curve

## **Table B.** Performance metrics across 10 folds of cross-validation from the intermediary **6-60-month** any variable model using the probability threshold that gave 80% sensitivity

| **Fold** | **Specificity** | **Sensitivity** | **AUC** | **PPV** | **NPV** | **PRAUC** | **Brier Score** |
| --- | --- | --- | --- | --- | --- | --- | --- |
| 1 | 0.652 | 0.783 | 0.751 | 0.101 | 0.984 | 0.140 | 0.044 |
| 2 | 0.481 | 0.792 | 0.720 | 0.074 | 0.978 | 0.162 | 0.045 |
| 3 | 0.509 | 0.783 | 0.707 | 0.074 | 0.979 | 0.111 | 0.045 |
| 4 | 0.557 | 0.783 | 0.719 | 0.081 | 0.981 | 0.141 | 0.044 |
| 5 | 0.680 | 0.783 | 0.773 | 0.109 | 0.984 | 0.206 | 0.042 |
| 6 | 0.553 | 0.783 | 0.747 | 0.081 | 0.981 | 0.143 | 0.043 |
| 7 | 0.554 | 0.783 | 0.793 | 0.081 | 0.981 | 0.181 | 0.043 |
| 8 | 0.560 | 0.783 | 0.753 | 0.082 | 0.981 | 0.182 | 0.043 |
| 9 | 0.724 | 0.792 | 0.812 | 0.130 | 0.985 | 0.177 | 0.045 |
| 10 | 0.728 | 0.792 | 0.814 | 0.132 | 0.985 | 0.186 | 0.044 |
| **Average** | **0.600** | **0.785** | **0.759** | **0.094** | **0.982** | **0.163** | **0.044** |

Abbreviations: AUC = area under the receiver operating characteristic curve; PPV = positive predictive value; NPV = negative predictive value; PRAUC = area under the precision-recall curve

**
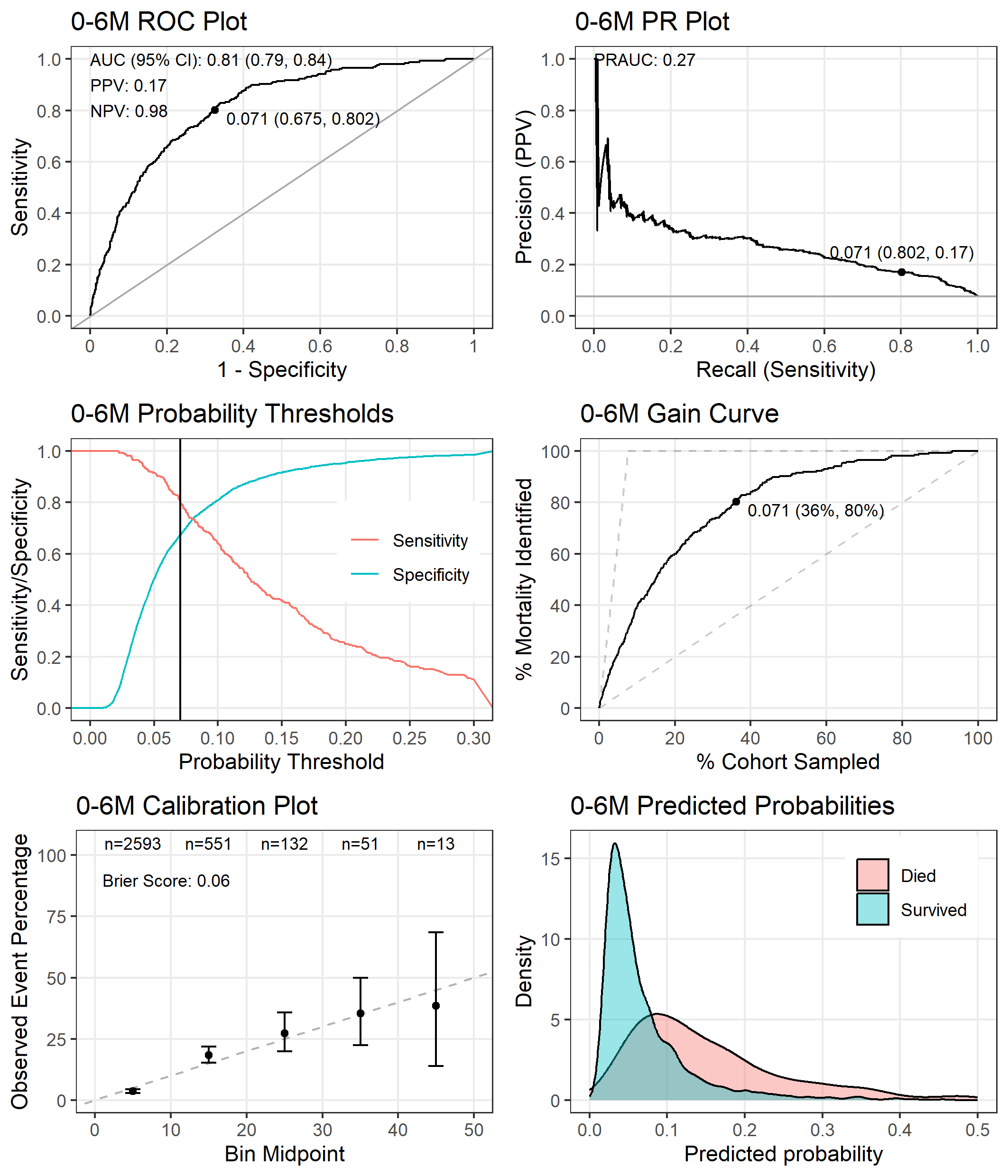
**

## **Figure A.** Performance of the intermediary **0-6-month** any variable model tested on the entire dataset.

The point on the receiver operating characteristic (ROC) plot, precision recall (PR) plot, and gain curve indicates the co-ordinates when using the probability threshold that gives a sensitivity of 80% (probability threshold = 0.071). The positive predictive value (PPV) and negative predictive value (NPV) are also reported in the ROC plot using this threshold.


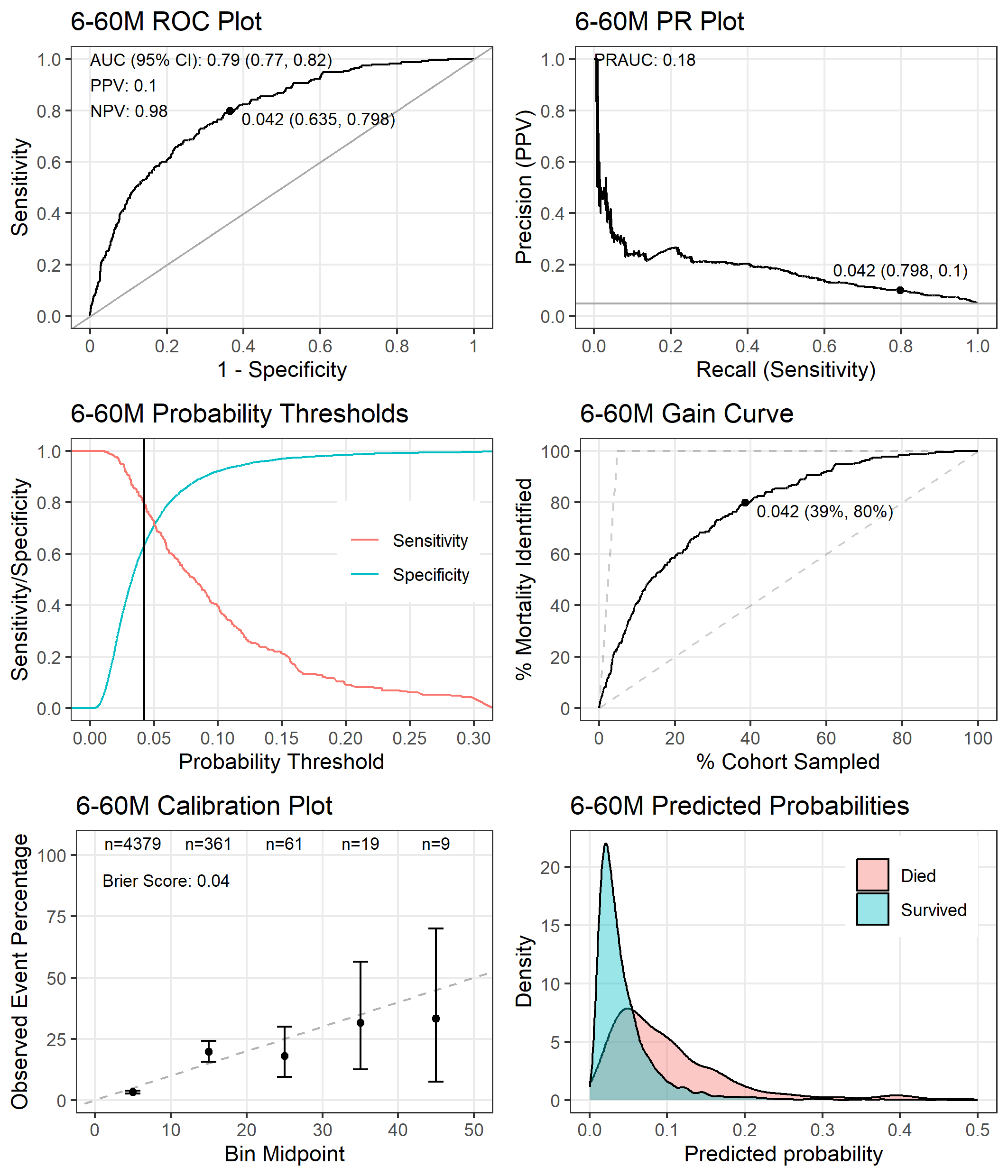


## **Figure B.** Performance of the intermediary **6-60-month** any variable model tested on the entire dataset.

The point on the receiver operating characteristic (ROC) plot, precision recall (PR) plot, and gain curve indicates the co-ordinates when using the probability threshold that gives a sensitivity of 80% (probability threshold = 0.042). The positive predictive value (PPV) and negative predictive value (NPV) are also reported in the ROC plot using this threshold.

## **Table C.** Coefficients of the intermediary **0-6-month** any variable model

| **Variable** | **Coefficient** |
| --- | --- |
| Intercept | -2.771 |
| Abdominal distension | 0.045 |
| Abnormal BCS | 0.022 |
| Number of children | -0.001 |
| BMI z-score | -0.009 |
| Dehydration WHO categories, severe dehydration | 0.013 |
| Duration of present illness, 8 days – 1 month | 0.094 |
| Duration of present illness, >1 month | 0.028 |
| Fontanelle | 0.087 |
| Glucose | 0.042 |
| Heart rate | -0.003 |
| Referral visit | 0.034 |
| Lactate | 0.065 |
| Malaria | -0.078 |
| Mother currently acutely ill | -0.029 |
| Maternal age | 0.009 |
| Maternal education | -0.069 |
| Maternal HIV, yes | 0.007 |
| MUAC | -0.257 |
| Neonate | -0.078 |
| Pallor | 0.047 |
| Prior care sought for current illness | 0.064 |
| How long since last admission, 7 days – 1 month | 0.049 |
| How long since last admission, 1 month – 1 year | 0.053 |
| Sex, male | 0.009 |
| SpO_2_ | -0.097 |
| Sucking well when breastfeeding, or feeding well if not breastfed | -0.135 |
| Temperature | -0.018 |
| Temperature-squared | -0.018 |
| When did the baby cry after birth, immediately | 0.063 |
| When did the baby cry after birth, 11-30 minutes | 0.009 |
| Abnormal tone | 0.075 |
| Time to reach hospital, 30 minutes – 1 hour | -0.036 |
| Time to reach hospital, >1 hour | 0.146 |
| Decreased urine production | 0.051 |
| Water source, municipal water | -0.025 |
| Water source, open source | 0.051 |
| Water source, fast running water | -0.032 |
| Weight for age z-score | -0.255 |
| Age × Abdominal distension | 0.012 |
| Age × Number of children | -0.045 |
| Age × Bed net use, sometimes | -0.020 |
| Age × Dehydration WHO categories, some dehydration | 0.021 |
| Age × Dehydration WHO categories, severe dehydration | 0.025 |
| Age × Delivery method, caesarean | -0.010 |
| Age × Diastolic blood pressure | -0.002 |
| Age × Glucose | 0.014 |
| Age × Referral visit | -0.010 |
| Age × Neonatal jaundice | 0.092 |
| Age × Malaria | -0.033 |
| Age × Mother currently acutely ill | -0.029 |
| Age × Maternal HIV, unknown | -0.017 |
| Age × Neonate | 0 |
| Age × Pallor | 0.027 |
| Age × Premature birth | -0.001 |
| Age × How long since last admission, <7 days | 0.073 |
| Age × How long since last admission, 7 days – 1 month | 0.011 |
| Age × SpO_2_ transformed | 0.034 |
| Age × Sucking well when breastfeeding, or feeding well if not breastfed, prior to illness | 0.058 |
| Age × Systolic blood pressure | -0.002 |
| Age × When did the baby cry after birth, immediately | 0.024 |
| Age × When did the baby cry after birth, <5 minutes | 0.009 |
| Age × Abnormal tone | 0.016 |
| Age × Boil/disinfect/filter water | 0.047 |
| Age × Weight for age z-score | -0.050 |

Interactions between variables are indicated by the multiplication sign.

Abbreviations: BCS = Blantyre coma scale; BMI = body mass index; HIV = human immunodeficiency virus; MUAC = mid-upper arm circumference; SpO_2_ = oxygen saturation; WHO = World Health Organisation

## **Table D.** Coefficients of the intermediary **6-60-month** any variable model.

| **Variable** | **Coefficient** |
| --- | --- |
| Intercept | -3.297 |
| Abnormal BCS | 0.099 |
| Bed net use, sometimes | 0.008 |
| Haemoglobin | -0.223 |
| HIV+ | 0.112 |
| Malaria | -0.113 |
| Maternal education | -0.029 |
| Maternal HIV, unknown | 0.019 |
| MUAC | -0.378 |
| How long since last admission, <7 days | 0.063 |
| How long since last admission, 7 days – 1 month | 0.128 |
| How long since last admission, 1 month – 1 year | 0.023 |
| How long since last admission, >1 year | -0.058 |
| Respiratory rate | 0.023 |
| SpO_2_ | -0.162 |
| Temperature | -0.104 |
| Temperature-squared | -0.096 |
| Time it took to reach hospital, >1 hour | 0.006 |
| Boil/disinfect/filter water | -0.095 |
| Water source, municipal water | -0.061 |
| Weight for age z-score | -0.223 |
| Age × Abnormal BCS | -0.008 |
| Age × Diastolic blood pressure | 0.001 |
| Age × How long since last admission, 7 days – 1 month | 0.005 |
| Age × How long since last admission, 1 month – 1 year | 0.060 |
| Age × Respiratory rate | 0.045 |
| Age × Time it took to reach hospital, >1 hour | 0.088 |
| Age × Water source, bore hole | 0.130 |
| Age × Water source, municipal water | -0.030 |
| Age × Weight for age z-score | 0.130 |

Interactions between variables are indicated by the multiplication sign.

Abbreviations: BCS = Blantyre coma scale; HIV = human immunodeficiency virus; MUAC = mid-upper arm circumference; SpO_2_ = oxygen saturation

## **Table E.** Average rank of variable importance and the number of times selected in the top 8 variables across 10 folds of cross-validation from the intermediary **0-6-month** any variable model.

Only the top 20 variables and interactions are shown. Interactions between variables are indicated by the multiplication sign. The top eight unique variables by average rank are highlighted in bold. Note that top eight variables are identical to those selected by the intermediary clinical and social model (see **Table A in S4 Text**).

| **Variable** | **Average Rank** | **Times Selected in Top 8** |
| --- | --- | --- |
| **Weight for age z-score** | **1.1** | **10** |
| **MUAC** | **1.9** | **10** |
| **Time it took to reach hospital, >1 hour** | **3.2** | **10** |
| **Sucking well when breastfeeding, or feeding well if not breastfed** | **4.2** | **10** |
| **SpO_2_** | **7**.**0** | **7** |
| **Duration of present illness, 8 days – 1 month** | **7.9** | **7** |
| **Age × Neonatal jaundice** | **8.3** | **6** |
| Abnormal tone | 10.3 | 4 |
| Fontanelle | 11.2 | 7 |
| Malaria | 14.0 | 1 |
| Neonate | 15.1 | 2 |
| Age × How long since last admission, <7 days | 15.1 | 1 |
| Prior care sought for current illness | 16.4 | 0 |
| Lactate | 16.7 | 0 |
| Maternal education | 17.6 | 1 |
| When did the baby cry after birth, immediately | 18.4 | 0 |
| Water source, open source | 20.8 | 0 |
| How long since last admission, 1 month – 1 year | 22.0 | 0 |
| Decreased urine production | 22.1 | 0 |
| How long since last admission, 7 days – 1 month | 24.7 | 0 |

Abbreviations: MUAC = mid-upper arm circumference; SpO_2_ = oxygen saturation

## **Table F.** Average rank of variable importance and the number of times selected in the top 8 variables across 10 folds of cross-validation from the intermediary **6-60-month** any variable model.

Only the top 20 variables and interactions are shown. Interactions between variables are indicated by the multiplication sign. The top eight unique variables by average rank are highlighted in bold.

| **Variable** | **Average Rank** | **Times Selected in Top 8** |
| --- | --- | --- |
| **MUAC** | **1.0** | **10** |
| **Haemoglobin** | **2.5** | **10** |
| **Weight for age z-score** | **2.7** | **10** |
| **SpO_2_** | **4.5** | **9** |
| **How long since last admission, 7 days – 1 month** | **7.1** | **7** |
| **Age × Water source, bore hole** | **7.9** | **5** |
| **HIV+** | **8.2** | **7** |
| Age × Weight for length z-score | 8.3 | 6 |
| Temperature | 9.7 | 2 |
| Malaria | 10.5 | 4 |
| Abnormal BCS | 11.2 | 3 |
| Boil/disinfect/filter water | 11.5 | 3 |
| Temperature-squared | 11.7 | 1 |
| Age × Time to reach hospital, >1 hour | 12.6 | 3 |
| Water source, municipal water | 15.8 | 0 |
| How long since last admission, <7 days | 16.5 | 0 |
| How long since last admission, >1 year | 17.8 | 0 |
| Age × How long since last admission, 1 month – 1 year | 15.9 | 0 |
| Age × Respiratory rate | 22.2 | 0 |
| Maternal education | 24.1 | 0 |

Abbreviations: BCS = Blantyre coma scale; HIV = human immunodeficiency virus; MUAC = mid-upper arm circumference; SpO_2_ = oxygen saturation
