# Supplementary material for: Prediction models for post-discharge mortality among under-five children with suspected sepsis in Uganda: A multicohort analysis
Source: PLOS Glob Public Health. 2024 Apr 29;4(4):e0003050. doi: 10.1371/journal.pgph.0003050 (PMC11057737; doi:10.1371/journal.pgph.0003050)
Supplement: S6 Text — (DOCX) [file pgph.0003050.s007.docx]

Prediction models for post-discharge mortality among under-five children with suspected sepsis in Uganda: A multicohort analysis

**Supplementary Material S6**

Contents

[S6: Final Clinical Variable Models, M6PD-C_0-6_ and M6PD-C_6-60_ – Performance Metrics and Coefficients 2](#_Toc163372754)

[**Table A.** Performance metrics across 10 folds of cross-validation from the **M6PD-C_0-6_ model** using the probability threshold that gave 80% sensitivity. 2](#_Toc163372755)

[**Table B.** Performance metrics across 10 folds of cross-validation from the **M6PD-C_6-60_ model** using the probability threshold that gave 80% sensitivity. 2](#_Toc163372756)

[**Table C.** Coefficients of the **M6PD-C_0-6_ model**. 3](#_Toc163372757)

[**Table D.** Coefficients of the **M6PD-C_6-60_ model**. 3](#_Toc163372758)

[**Figure A.** Variable importance plot of the **M6PD-C_0-6_** model 4](#_Toc163372759)

[**Figure B.** Variable importance plot of the **M6PD-C_6-60_** model 4](#_Toc163372760)

[**Figure C.** Zoomed in calibration plot of the **M6PD-C_0-6_** model for predicted probabilities between 0-10%. 5](#_Toc163372761)

[**Figure D.** Zoomed in calibration plot of the **M6PD-C_6-60_** model for predicted probabilities between 0-10%. 5](#_Toc163372762)

# S6: Final Clinical Variable Models, M6PD-C_0-6_ and M6PD-C_6-60_ – Performance Metrics and Coefficients

## **Table A.** Performance metrics across 10 folds of cross-validation from the **M6PD-C_0-6_ model** using the probability threshold that gave 80% sensitivity.

The top eight unique variables with the highest average variable importance from 10-fold cross-validation in the intermediary clinical variable model were used (see **Table A in S3 Text**).

| **Fold** | **Specificity** | **Sensitivity** | **AUC** | **PPV** | **NPV** | **PRAUC** | **Brier Score** |
| --- | --- | --- | --- | --- | --- | --- | --- |
| 1 | 0.757 | 0.808 | 0.848 | 0.219 | 0.979 | 0.331 | 0.064 |
| 2 | 0.440 | 0.808 | 0.688 | 0.108 | 0.965 | 0.159 | 0.070 |
| 3 | 0.616 | 0.808 | 0.722 | 0.150 | 0.974 | 0.186 | 0.068 |
| 4 | 0.725 | 0.800 | 0.814 | 0.190 | 0.978 | 0.302 | 0.063 |
| 5 | 0.429 | 0.808 | 0.694 | 0.106 | 0.964 | 0.248 | 0.067 |
| 6 | 0.589 | 0.800 | 0.763 | 0.136 | 0.973 | 0.296 | 0.063 |
| 7 | 0.663 | 0.808 | 0.795 | 0.168 | 0.976 | 0.196 | 0.068 |
| 8 | 0.715 | 0.808 | 0.795 | 0.193 | 0.978 | 0.206 | 0.068 |
| 9 | 0.583 | 0.800 | 0.794 | 0.134 | 0.973 | 0.218 | 0.065 |
| 10 | 0.469 | 0.808 | 0.632 | 0.114 | 0.967 | 0.113 | 0.072 |
| **Average** | **0.599** | **0.805** | **0.754** | **0.152** | **0.973** | **0.226** | **0.067** |

Abbreviations: AUC = area under the receiver operating characteristic curve; PPV = positive predictive value; NPV = negative predictive value; PRAUC = area under the precision-recall curve

## **Table B.** Performance metrics across 10 folds of cross-validation from the **M6PD-C_6-60_ model** using the probability threshold that gave 80% sensitivity.

The top nine unique variables with the highest average variable importance from 10-fold cross-validation in the intermediary clinical variable model were used (see **Table B in S3 Text**).

| **Fold** | **Specificity** | **Sensitivity** | **AUC** | **PPV** | **NPV** | **PRAUC** | **Brier Score** |
| --- | --- | --- | --- | --- | --- | --- | --- |
| 1 | 0.737 | 0.783 | 0.768 | 0.129 | 0.985 | 0.139 | 0.043 |
| 2 | 0.606 | 0.792 | 0.748 | 0.095 | 0.982 | 0.171 | 0.045 |
| 3 | 0.483 | 0.783 | 0.675 | 0.070 | 0.978 | 0.099 | 0.045 |
| 4 | 0.304 | 0.783 | 0.665 | 0.053 | 0.966 | 0.139 | 0.043 |
| 5 | 0.535 | 0.783 | 0.750 | 0.078 | 0.980 | 0.214 | 0.042 |
| 6 | 0.588 | 0.783 | 0.756 | 0.087 | 0.982 | 0.173 | 0.042 |
| 7 | 0.580 | 0.783 | 0.737 | 0.085 | 0.982 | 0.137 | 0.044 |
| 8 | 0.549 | 0.783 | 0.699 | 0.080 | 0.981 | 0.149 | 0.044 |
| 9 | 0.772 | 0.792 | 0.798 | 0.153 | 0.986 | 0.166 | 0.045 |
| 10 | 0.520 | 0.792 | 0.723 | 0.079 | 0.98 | 0.187 | 0.044 |
| **Average** | **0.567** | **0.785** | **0.732** | **0.091** | **0.98** | **0.157** | **0.044** |

Abbreviations: AUC = area under the receiver operating characteristic curve; PPV = positive predictive value; NPV = negative predictive value; PRAUC = area under the precision-recall curve

## **Table C.** Coefficients of the **M6PD-C_0-6_ model**.

| **Variable** | **Coefficient** |
| --- | --- |
| Intercept | -2.760 |
| Weight for age z-score | -0.344 |
| MUAC | -0.295 |
| Sucking well when breastfeeding, or feeding well if not breastfed | -0.248 |
| SpO_2_ | -0.165 |
| Duration of present illness, 48 hours – 7 days | 0.023 |
| Duration of present illness, 8 days – 1 month | 0.194 |
| Duration of present illness, >1 month | 0.068 |
| Age | 0.068 |
| Fontanelle | 0.134 |
| Age × Weight for age z-score | -0.054 |
| Age × Sucking well when breastfeeding | 0.023 |
| Age × Duration of present illness, 48 hours – 7 days | 0.060 |
| Age × Jaundice | 0.101 |

Interactions between variables are indicated by the multiplication sign.

Abbreviations: MUAC = mid-upper arm circumference; SpO_2_ = oxygen saturation

## **Table D.** Coefficients of the **M6PD-C_6-60_ model**.

| **Variable** | **Coefficient** |
| --- | --- |
| Intercept | -3.243 |
| MUAC | -0.411 |
| SpO_2_ | -0.186 |
| Weight for age z-score | -0.177 |
| Age | 0.029 |
| How long since last admission, <7 days | 0.083 |
| How long since last admission, 7 days – 1 month | 0.142 |
| How long since last admission, 1 month – 1 year | 0.030 |
| How long since last admission, >1 year | -0.081 |
| Respiratory rate | 0.054 |
| Abnormal BCS | 0.156 |
| Temperature, °C | -0.123 |
| Temperature-squared, °C | -0.116 |
| HIV+ | 0.127 |
| Age × MUAC | 0.016 |
| Age × SpO_2_ | 0.011 |
| Age × How long since last admission, <7 days | 0.026 |
| Age × How long since last admission, 7 days – 1 month | 0.023 |
| Age × How long since last admission, 1 month – 1 year | 0.086 |
| Age × How long since last admission >1 year | -0.012 |
| Age × Respiratory rate | 0.106 |
| Age × Abnormal BCS | -0.050 |
| Age × HIV+ | -0.021 |

Interactions between variables are indicated by the multiplication sign.

Abbreviations: BCS = Blantyre coma scale; HIV = human immunodeficiency virus; MUAC = mid-upper arm circumference; SpO_2_ = oxygen saturation


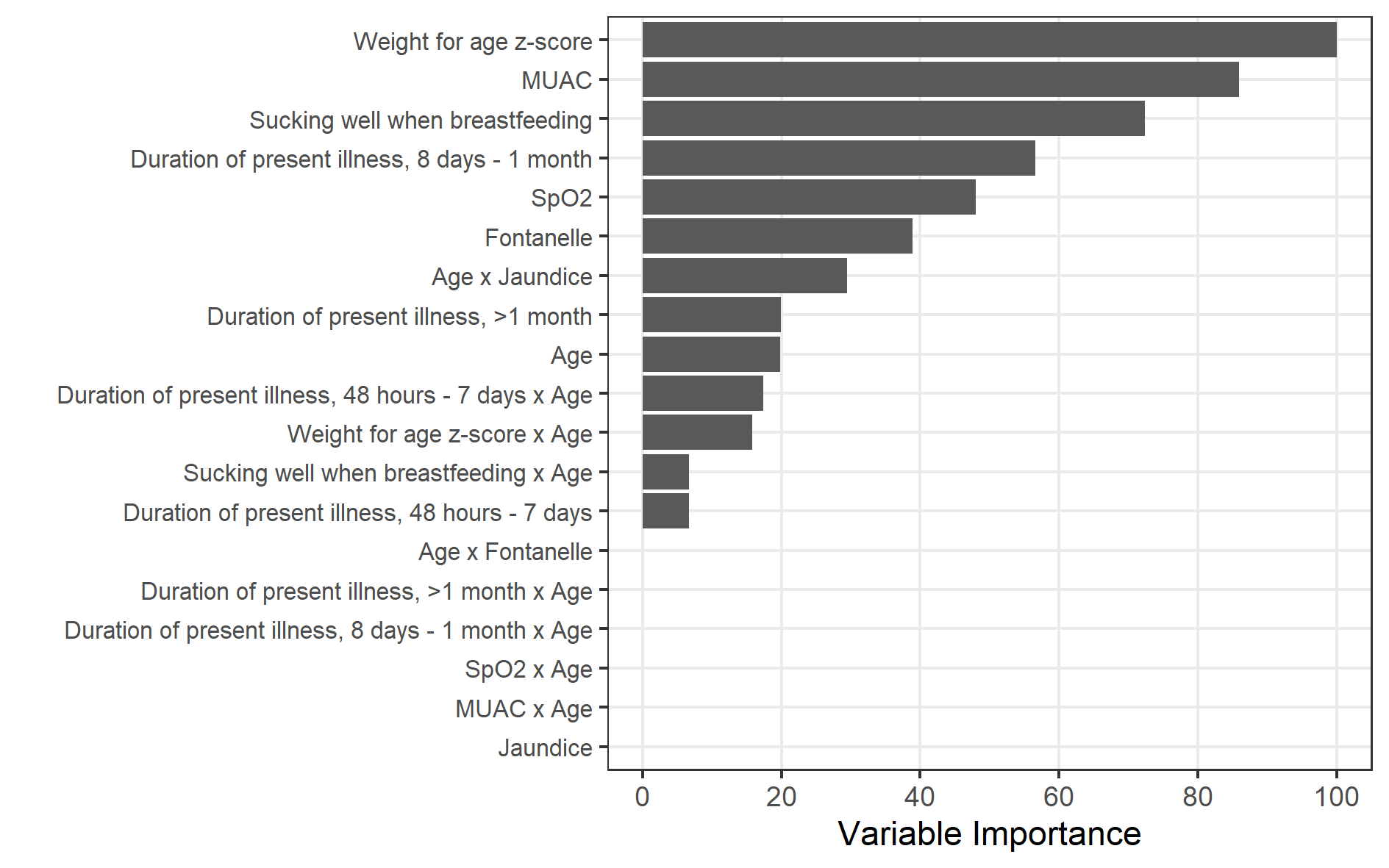


## **Figure A.** Variable importance plot of the **M6PD-C_0-6_** model

_
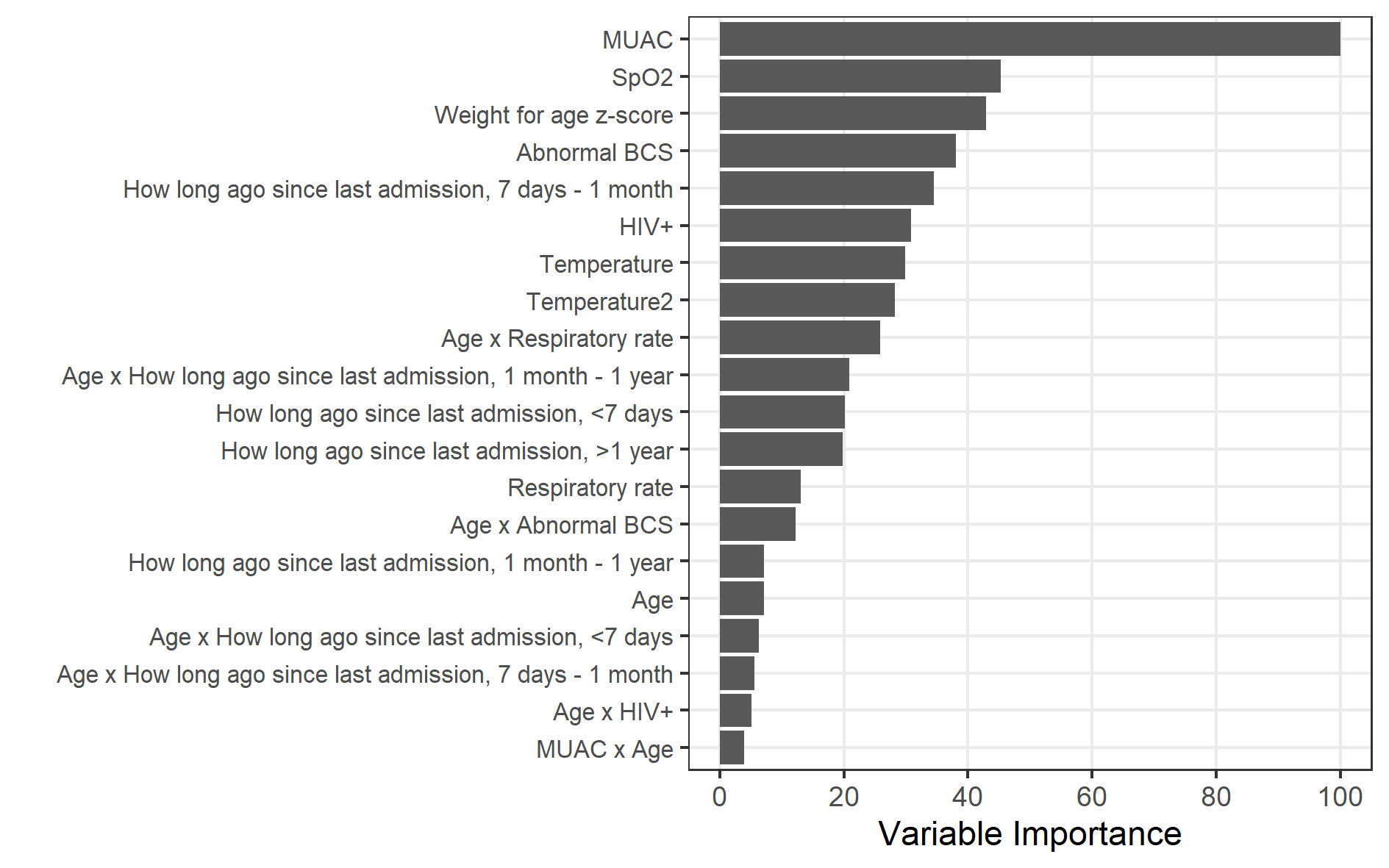
_

## **Figure B.** Variable importance plot of the **M6PD-C_6-60_** model


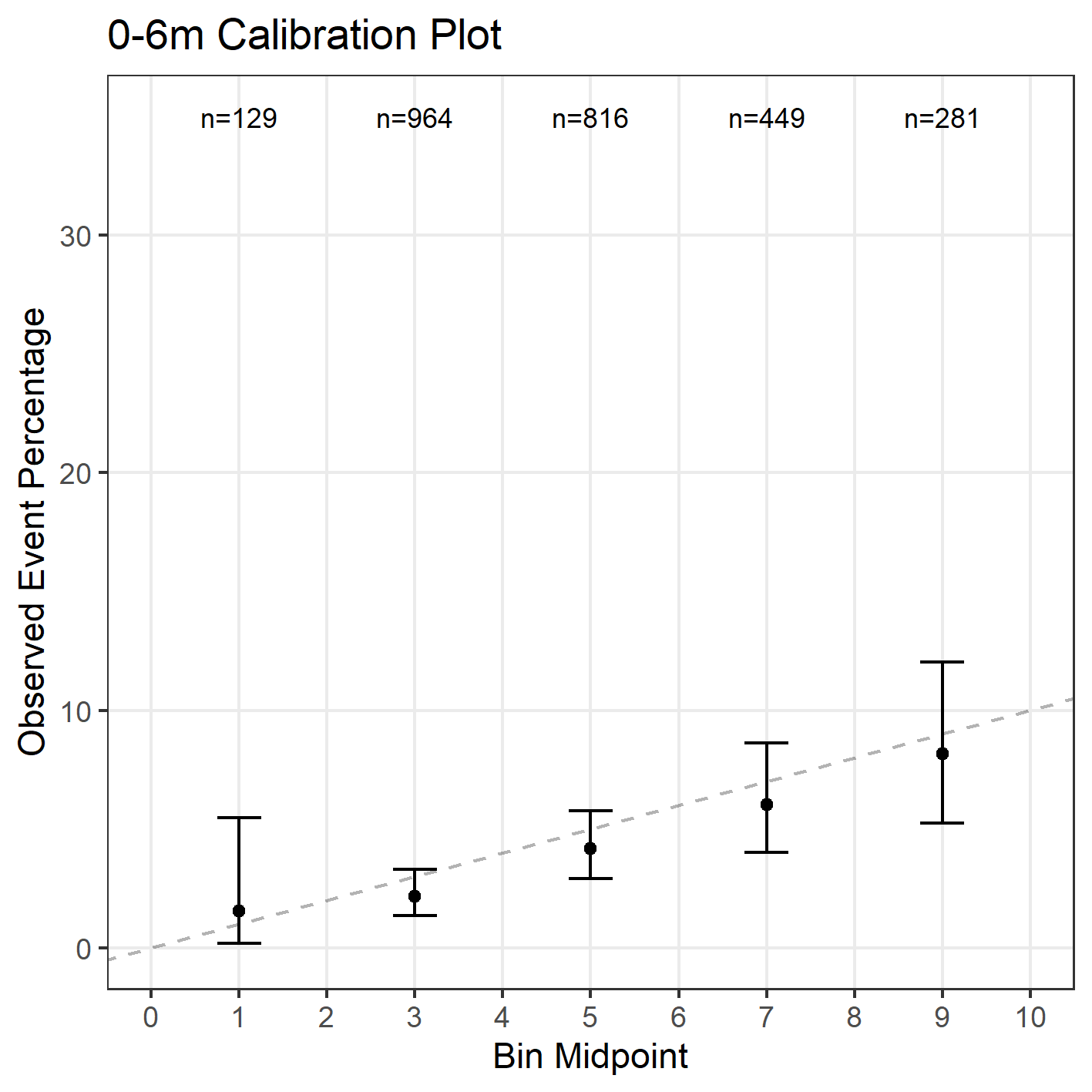


## **Figure C.** Zoomed in calibration plot of the **M6PD-C_0-6_** model for predicted probabilities between 0-10%.


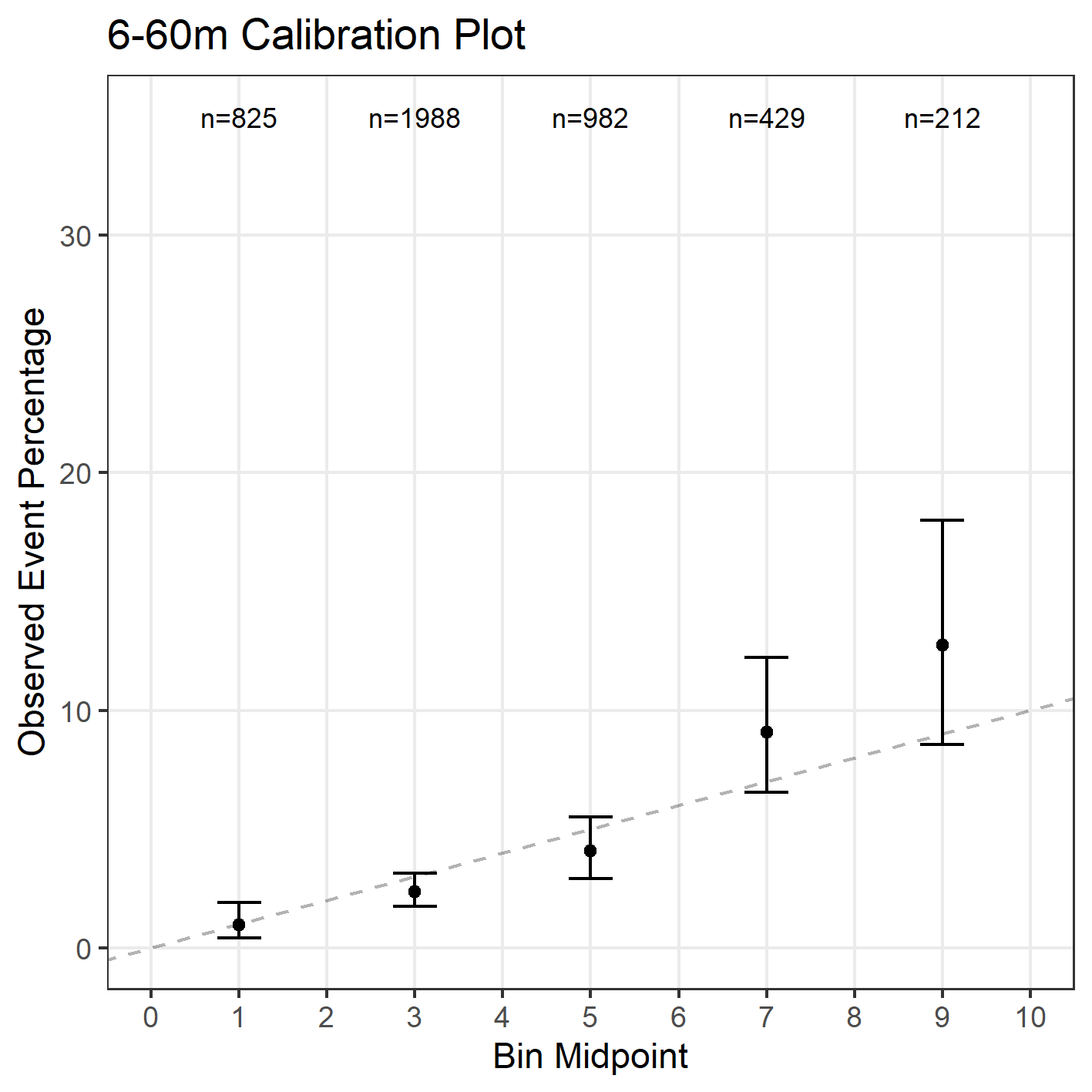


## **Figure D.** Zoomed in calibration plot of the **M6PD-C_6-60_** model for predicted probabilities between 0-10%.
