# Supplementary material for: Prediction models for post-discharge mortality among under-five children with suspected sepsis in Uganda: A multicohort analysis
Source: PLOS Glob Public Health. 2024 Apr 29;4(4):e0003050. doi: 10.1371/journal.pgph.0003050 (PMC11057737; doi:10.1371/journal.pgph.0003050)
Supplement: S7 Text — (DOCX) [file pgph.0003050.s008.docx]

Prediction models for post-discharge mortality among under-five children with suspected sepsis in Uganda: A multicohort analysis

**Supplementary Material S7**

Contents

[S7: Final Clinical and Social Variable Models, M6PD-CS_0-6_ and M6PD-CS_6-60_ – Performance Metrics and Coefficients 2](#_Toc163373318)

[**Table A.** Performance metrics across 10 folds of cross-validation from **M6PD-CS_0-6_ model** using the probability threshold that gave 80% sensitivity. 2](#_Toc163373319)

[**Table B.** Performance metrics across 10 folds of cross-validation from **M6PD-CS_6-60_ model** using the probability threshold that gave 80% sensitivity. 2](#_Toc163373320)

[**Figure A.** Performance of the **M6PD-CS_0-6_ model** tested on the entire dataset. 3](#_Toc163373321)

[**Figure B.** Performance of the **M6PD-CS_6-60_ model** tested on the entire dataset. 4](#_Toc163373322)

[**Table C.** Coefficients of the **M6PD-CS_0-6_ model**. 5](#_Toc163373323)

[**Table D.** Coefficients of the **M6PD-CS_6-60_ model**. 5](#_Toc163373324)

[**Figure C.** Variable importance plot of the **M6PD-CS_0-6_** model 6](#_Toc163373325)

[**Figure D.** Variable importance plot of the **M6PD-CS_6-60_** model 6](#_Toc163373326)

# S7: Final Clinical and Social Variable Models, M6PD-CS_0-6_ and M6PD-CS_6-60_ – Performance Metrics and Coefficients

Note that the **M6PD-CS_0-6_ model** is identical to **M6PD-A_0-6_** reported in S8 Text.

## **Table A.** Performance metrics across 10 folds of cross-validation from **M6PD-CS_0-6_ model** using the probability threshold that gave 80% sensitivity.

The top eight unique variables with the highest average variable importance from 10-fold cross-validation in the intermediary clinical and social variable model were used (see **Table A in S4 Text**).

| **Fold** | **Specificity** | **Sensitivity** | **AUC** | **PPV** | **NPV** | **PRAUC** | **Brier Score** |
| --- | --- | --- | --- | --- | --- | --- | --- |
| 1 | 0.832 | 0.808 | 0.853 | 0.288 | 0.981 | 0.354 | 0.063 |
| 2 | 0.408 | 0.808 | 0.709 | 0.103 | 0.962 | 0.162 | 0.070 |
| 3 | 0.545 | 0.808 | 0.715 | 0.130 | 0.971 | 0.153 | 0.070 |
| 4 | 0.731 | 0.800 | 0.848 | 0.194 | 0.978 | 0.342 | 0.062 |
| 5 | 0.513 | 0.808 | 0.710 | 0.122 | 0.970 | 0.227 | 0.067 |
| 6 | 0.508 | 0.800 | 0.756 | 0.116 | 0.969 | 0.278 | 0.064 |
| 7 | 0.709 | 0.808 | 0.812 | 0.189 | 0.978 | 0.207 | 0.067 |
| 8 | 0.728 | 0.808 | 0.805 | 0.200 | 0.978 | 0.208 | 0.067 |
| 9 | 0.686 | 0.800 | 0.791 | 0.171 | 0.977 | 0.192 | 0.065 |
| 10 | 0.421 | 0.808 | 0.655 | 0.105 | 0.963 | 0.126 | 0.071 |
| **Average** | **0.608** | **0.805** | **0.765** | **0.162** | **0.973** | **0.225** | **0.067** |

Abbreviations: AUC = area under the receiver operating characteristic curve; PPV = positive predictive value; NPV = negative predictive value; PRAUC = area under the precision-recall curve

## **Table B.** Performance metrics across 10 folds of cross-validation from **M6PD-CS_6-60_ model** using the probability threshold that gave 80% sensitivity.

The top eight unique variables with the highest average variable importance from 10-fold cross-validation in the intermediary clinical and social variable model were used (see **Table B in S4 Text**).

| **Fold** | **Specificity** | **Sensitivity** | **AUC** | **PPV** | **NPV** | **PRAUC** | **Brier Score** |
| --- | --- | --- | --- | --- | --- | --- | --- |
| 1 | 0.570 | 0.783 | 0.729 | 0.083 | 0.981 | 0.108 | 0.044 |
| 2 | 0.473 | 0.792 | 0.681 | 0.073 | 0.977 | 0.118 | 0.046 |
| 3 | 0.702 | 0.783 | 0.769 | 0.116 | 0.985 | 0.129 | 0.044 |
| 4 | 0.441 | 0.783 | 0.652 | 0.065 | 0.976 | 0.131 | 0.044 |
| 5 | 0.683 | 0.783 | 0.766 | 0.110 | 0.984 | 0.210 | 0.042 |
| 6 | 0.651 | 0.783 | 0.761 | 0.101 | 0.984 | 0.169 | 0.043 |
| 7 | 0.622 | 0.783 | 0.762 | 0.094 | 0.983 | 0.154 | 0.043 |
| 8 | 0.488 | 0.783 | 0.713 | 0.071 | 0.978 | 0.171 | 0.043 |
| 9 | 0.628 | 0.792 | 0.784 | 0.100 | 0.983 | 0.137 | 0.045 |
| 10 | 0.674 | 0.792 | 0.793 | 0.112 | 0.984 | 0.210 | 0.044 |
| **Average** | **0.593** | **0.785** | **0.741** | **0.093** | **0.982** | **0.154** | **0.044** |

Abbreviations: AUC = area under the receiver operating characteristic curve; PPV = positive predictive value; NPV = negative predictive value; PRAUC = area under the precision-recall curve


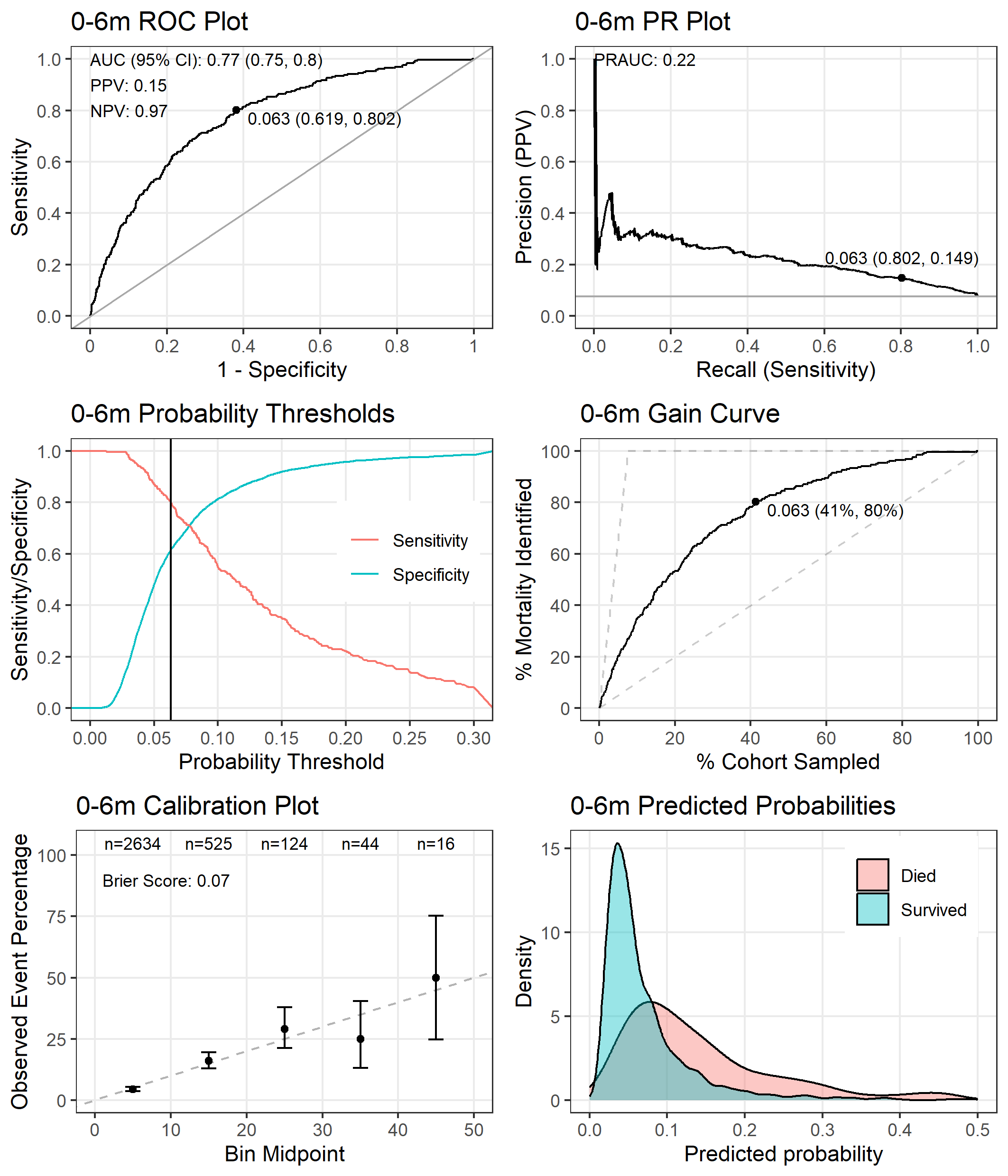


## **Figure A.** Performance of the **M6PD-CS_0-6_ model** tested on the entire dataset.

The point on the receiver operating characteristic (ROC) plot, precision recall (PR) plot, and gain curve indicates the co-ordinates when using the probability threshold that gives a sensitivity of 80% (probability threshold = 0.063). The positive predictive value (PPV) and negative predictive value (NPV) are also reported in the ROC plot using this threshold.


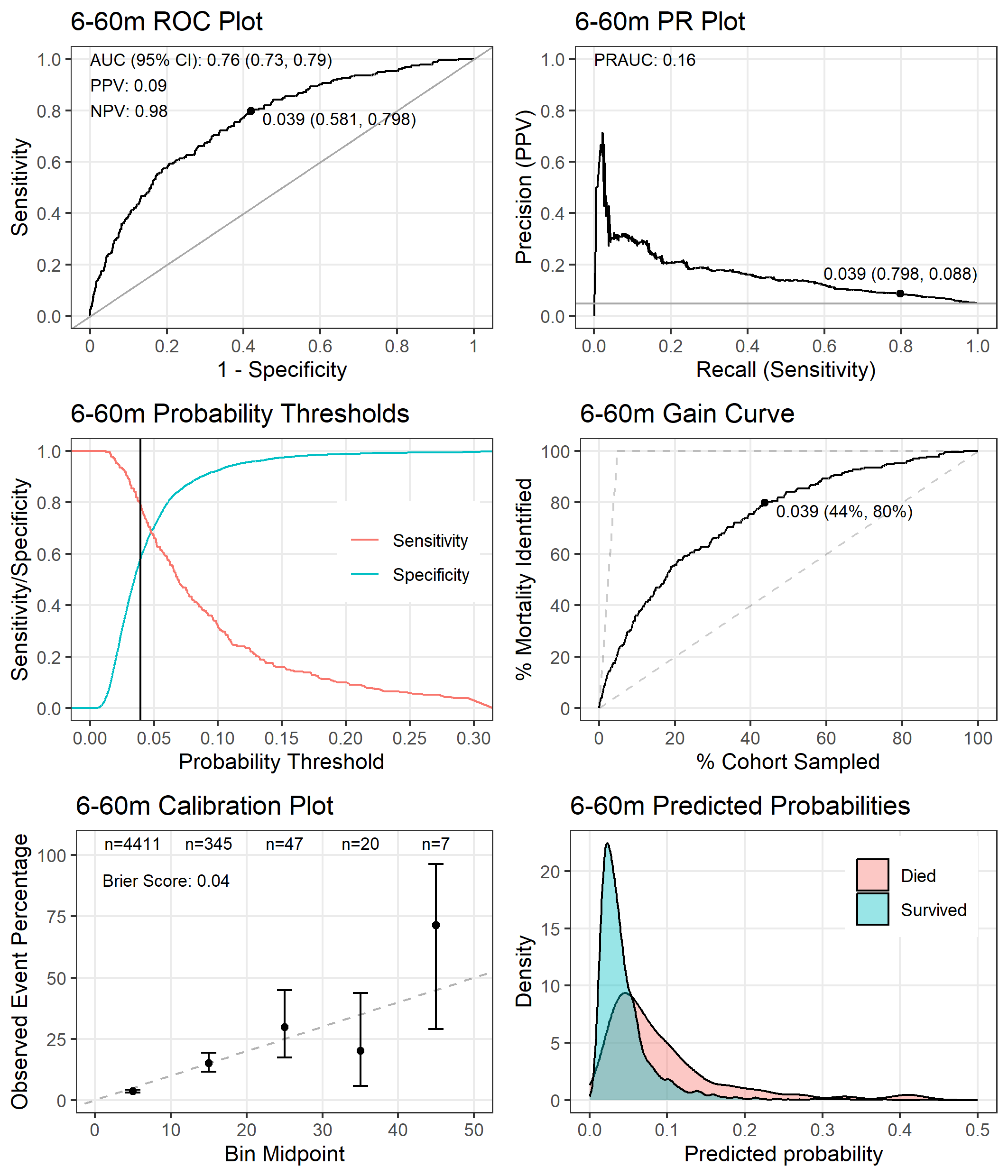


## **Figure B.** Performance of the **M6PD-CS_6-60_ model** tested on the entire dataset.

The point on the receiver operating characteristic (ROC) plot, precision recall (PR) plot, and gain curve indicates the co-ordinates when using the probability threshold that gives a sensitivity of 80% (probability threshold = 0.039). The positive predictive value (PPV) and negative predictive value (NPV) are also reported in the ROC plot using this threshold.

## **Table C.** Coefficients of the **M6PD-CS_0-6_ model**.

| **Variable** | **Coefficient** |
| --- | --- |
| Intercept | -2.753 |
| Weight for age z-score | -0.361 |
| MUAC | -0.219 |
| Time it took to reach hospital, >1 hour | 0.233 |
| Sucking well when breastfeeding, or feeding well if not breastfed | -0.206 |
| SpO_2_ | -0.149 |
| Duration of present illness, 8 days – 1 month | 0.130 |
| Duration of present illness, >1 month | 0.031 |
| Age | 0.031 |
| Age × Weight for age z-score | -0.064 |
| Age × Duration of present illness, 48 hours – 7 days | 0.005 |
| Age × Jaundice | 0.087 |

Interactions between variables are indicated by the multiplication sign.

Abbreviations: MUAC = mid-upper arm circumference; SpO_2_ = oxygen saturation

## **Table D.** Coefficients of the **M6PD-CS_6-60_ model**.

| **Variable** | **Coefficient** |
| --- | --- |
| Intercept | -3.241 |
| MUAC | -0.386 |
| Weight for age z-score | -0.180 |
| SpO_2_ | -0.186 |
| HIV+ | 0.111 |
| Age | 0.007 |
| Water source, municipal water | -0.102 |
| How long since last admission, <7 days | 0.074 |
| How long since last admission, 7 days – 1 month | 0.124 |
| How long since last admission, 1 month – 1 year | 0.013 |
| How long since last admission, >1 year | -0.059 |
| Boil/disinfect/filter water | -0.140 |
| Age × Water source, bore hole | 0.157 |
| Age × Water source, municipal water | -0.044 |
| Age × How long since last admission, <7 days | 0.006 |
| Age × How long since last admission, 7 days – 1 month | 0.007 |
| Age × How long since last admission, 1 month – 1 year | 0.077 |

Interactions between variables are indicated by the multiplication sign.

Abbreviations: HIV = human immunodeficiency virus; MUAC = mid-upper arm circumference; SpO_2_ = oxygen saturation


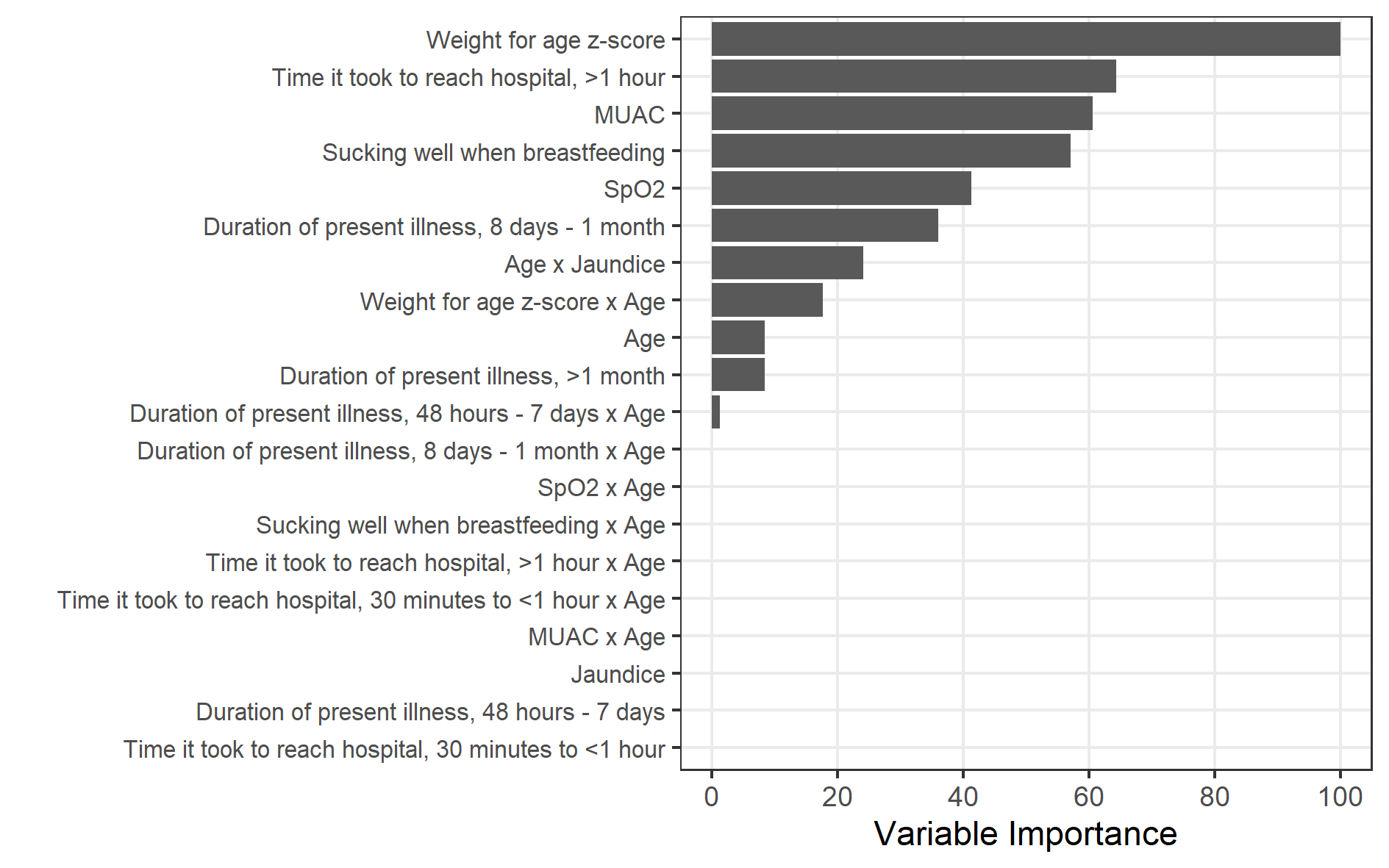


## **Figure C.** Variable importance plot of the **M6PD-CS_0-6_** model


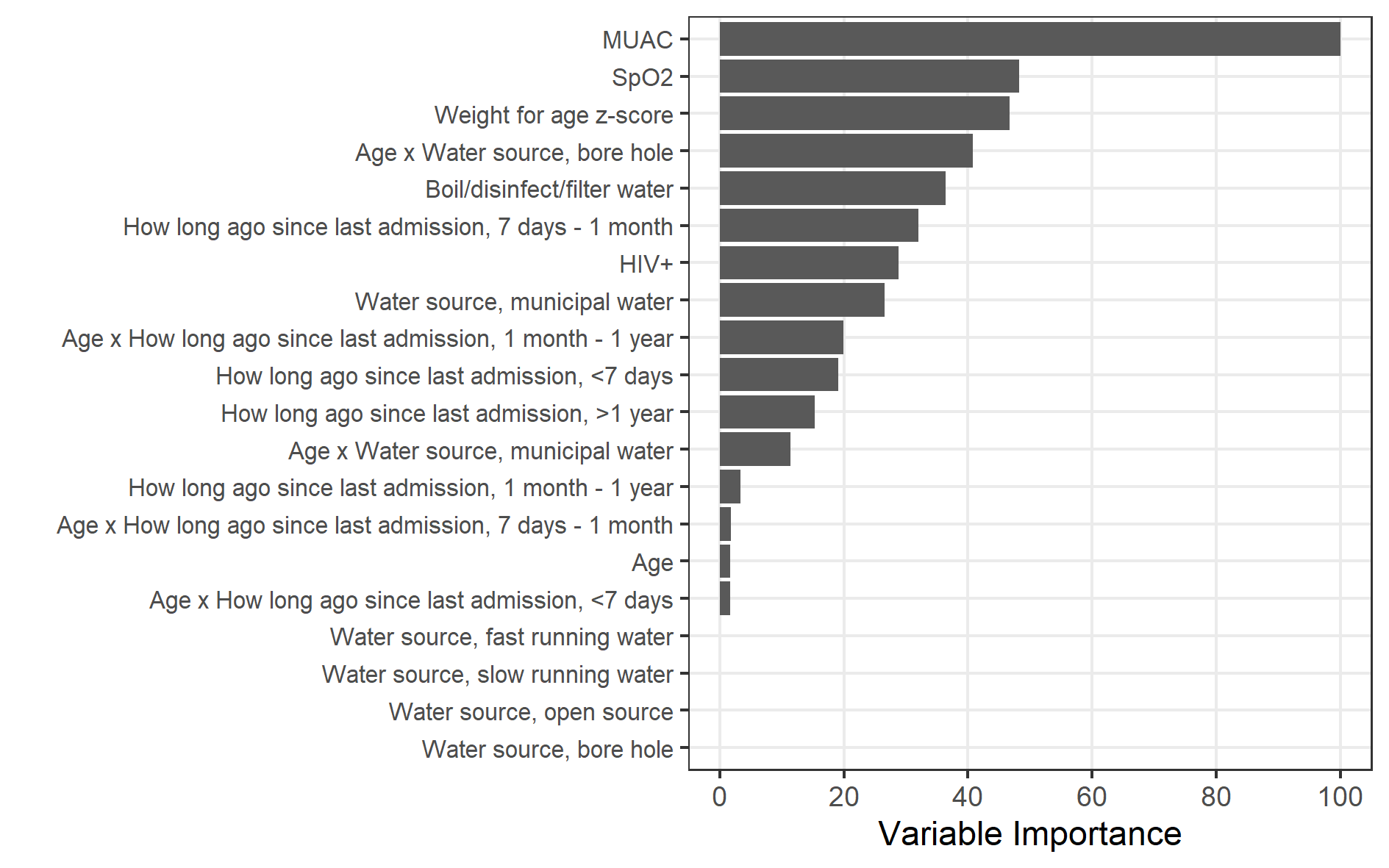


## **Figure D.** Variable importance plot of the **M6PD-CS_6-60_** model
