# Supplementary material for: Prediction models for post-discharge mortality among under-five children with suspected sepsis in Uganda: A multicohort analysis
Source: PLOS Glob Public Health. 2024 Apr 29;4(4):e0003050. doi: 10.1371/journal.pgph.0003050 (PMC11057737; doi:10.1371/journal.pgph.0003050)
Supplement: S9 Text — (DOCX) [file pgph.0003050.s010.docx]

Prediction models for post-discharge mortality among under-five children with suspected sepsis in Uganda: A multicohort analysis

**Supplementary Material S9**

Contents

[S9: Literature review 2](#_Toc163373956)

[**Table A.** Post-discharge risk prediction modelling study characteristics 2](#_Toc163373957)

[References 2](#_Toc163373958)

[**Table B.** Post-discharge model characteristics and reporting 3](#_Toc163373959)

# S9: Literature review

## **Table A.** Post-discharge risk prediction modelling study characteristics

| **Reference** | **Study population** | **Age group** | **Discharges/post-discharge deaths** | **Post-discharge follow-up window** | **Multiple models developed (Y/N)** |
| --- | --- | --- | --- | --- | --- |
| **CHAIN Network, 2022^1^** | Acute illness | 2 to 23 months | 2874/168 | 6 months | N |
| **Madrid** et al., **2019^2^** | All admissions | 1 to 15 years | 25632/935 | 90 days | Y |
| **Mwangome** et al., **2017^3^** | All admissions | 4 weeks to 6 months | 1455/75 | 12 months | Y |
| **Ngari** et al., **2017^4^** | Severe pneumonia | 1 to 59 months | 2324/70 | 12 months | Y |
| **Talbert** et al., **2019^5^** | Diarrhea | 2 to 59 months | 2505/49 | 12 months | N |
| **Wiens** et al., **2015^6^** | Proven or suspected infection | 6 months to 5 years | 1242/61 | 6 months | Y |

## References

1. The Childhood Acute Illness and Nutrition (CHAIN) Network*.* Childhood mortality during and after acute illness in Africa and south Asia: a prospective cohort study. *Lancet Glob Health* 2022; **10**: e673–684.
2. Madrid L, Casellas A, Sacoor C, *et al.* Postdischarge mortality prediction in Sub-Saharan Africa. *Pediatrics* 2019; **143**: e20180606.
3. Mwangome M, Ngari M, Fegan G, *et al.* Diagnostic criteria for severe acute malnutrition among infants aged under 6 mo. *Am J Clin Nutr* 2017; **105**: 1415–1423.
4. Ngari MM, Fegan G, Mwangome MK, *et al.* Mortality after inpatient treatment for severe pneumonia in children: a cohort study. *Paediatr Perinat Epidemiol* 2017; **31**: 233–242.
5. Talbert A, Ngari M, Bauni E, *et al.* Mortality after inpatient treatment for diarrhea in children: a cohort study. *BMC Med* 2019; published online January 28. DOI:10.1186/s12916-019-1258-0.
6. Wiens MO, Kumbakumba E, Larson CP, *et al.* Postdischarge mortality in children with acute infectious diseases: derivation of postdischarge mortality prediction models. *BMJ Open* 2015; **5**: e009449

## **Table B.** Post-discharge model characteristics and reporting

| **Reference** | **Model equation reported (Y/N)** | **Variables used** | **Number of variables in primary model** | **AUC (95% CI)** | **External validation (Y/N)** | **Explicit design for clinical use (Y/N)** |
| --- | --- | --- | --- | --- | --- | --- |
| **CHAIN Network, 2022^1^** | N | Individual variables: Age, sex, HIV status, admission duration, discharged against medical advice, nutritional strata at admission, change in nutritional strata at discharge  Exposure domains: Signs of illness severity at discharge (SIRS, respiratory distress, circulation, dehydration, severe anemia), signs of illness severity at admission (SIRS, respiratory distress, circulation, conscious level, dehydration, blood glucose, severe anemia), underlying medical conditions (small size at birth, height-for-age Z score, prior hospitalization, chronic conditions), child-level nutritional risk exposures (recommended appropriate diet, recent weight loss, poor feeding), caregiver characteristics (biological mother as primary caregiver, caregiver education level, mother mental health, mother sick, mother working), household-level exposures (assets quintiles, food insecurity, type of toilet, water availability), access to health care (distance to nearest health facility, means of travel to hospital, travel cost, travel time) | 39 | 0.81 (0.77-0.84) | N | N |
| **Madrid** et al., **2019^2^** | N | Age, rainy season, weight-for-height Z-score, history of diarrhea, history of cough, breathing difficulties, increased respiratory rate, skin pinch goes back slowly, nasal flaring, auscultatory crackles, oral candidiasis, edema (any location), depigmented or reddish hair, swollen lymph nodes, ear discharge, prostration, malaria diagnosis result, blood culture result, HIV status, outcome of the admission | 19 | Model 1: 0.79 (0.75-0.82)  Model 2: 0.78 (0.75-0.82)  Model 3: 0.75 (0.71-0.78)  Model 4: 0.76 (0.72-0.91) | N | Y |
| **Mwangome** et al., **2017^3^** | N | Age, sex, small size at birth, prior admission, HIV test positive, missing HIV results, weight-for-length Z-score, weight-for-age Z-score, length-for-age Z-score, MUAC | 8 | Model 1: 0.73 (0.67, 0.79)  Model 2: 0.80 (0.75, 0.86)  Model 3: 0.73 (0.67, 0.79)  Model 4: 0.81 (0.76, 0.86) | N | N |
| **Ngari** et al., **2017^4^** | N | Age, sex, distance from facility, reported premature/LBW, hospitalization time, previous admission, hypoxia, capillary refill >2 seconds, impaired consciousness, wheezing, cough >14 days, jaundice, severe anemia, axillary temperature, HIV test result, RSV test result, malaria slide result, bacteremia, MUAC, weight-for-length Z-score, weight-for-age Z-score, length-for-age Z-score, year of admission | 19 | Model 1: 0.91 (0.88, 0.95)  Model 2: 0.91 (0.88, 0.95)  Model 3: 0.92 (0.89, 0.95) | N | N |
| **Talbert** et al., **2019^5^** | Y | Prior hospital admission, lower chest wall indrawing, HIV antibody positive, bacteremia, MUAC | 5 | 0.87 (0.81–0.94) | N | N |
| **Wiens** et al., **2015^6^** | Y | MUAC, SpO_2_, time since last hospitalization, HIV positive, abnormal BCS | 5 | Model 1: 0.82 (0.75-0.87)  Model 2: 0.81 (0.75-0.87)  Model 3: 0.80 (0.74-0.86)  Model 4: 0.80 (0.73-0.86) | N | Y |

Abbreviations: BCS=Blantyre coma scale; HIV=Human immunodeficiency virus; LBW=Low birth weight; MUAC=Middle-upper arm circumference; SIRS: Systemic Inflammatory Response Syndrome; SpO_2_= Oxygen saturation
