# Supplementary material for: Prediction models for post-discharge mortality among under-five children with suspected sepsis in Uganda: A multicohort analysis
Source: PLOS Glob Public Health. 2024 Apr 29;4(4):e0003050. doi: 10.1371/journal.pgph.0003050 (PMC11057737; doi:10.1371/journal.pgph.0003050)
Supplement: Attachment — Submitted filename: Review Comments][Feb 8, 2024.doc [file pgph.0003050.s011.doc]

**REVIEWER REPORT**

**TOPIC**

Prediction models for post-discharge mortality among under-five children with suspected sepsis in Uganda: A multicohort analysis

**Remark(s) 1:** Reads fine.

**ABSTRACT**

**Introduction:** “Identifying those at risk has limited progress to improve outcomes.”

**Remark(s) 2, page 2, line 3:** This sentence does not read well. Recast, please.

**Methods:** Adequate.

**Results:** Adequate.

**Conclusion:** Adequate.

**INTRODUCTION**

**Page 3, para 2, lines 9 & 10:** “Robust epidemiological data for pediatric post-discharge mortality in the context of sepsis and severe infection have been limited.”

**Remark(s) 3:** Provide reference for this statement.

**MATERIALS AND METHODS**

**Protocol**

Detailed and rigorous

**RESULTS**

Adequately presented

**DISCUSSION**

**Remark(s):** While I credit the authors for a thorough presentation of this session, the session must be re-written completely. Typically, discussions are expected to highlight the main findings of the current study (not a mere repeat of the results) and show evidence of how these findings affirm or disaffirm existing evidence or previous studies. This way, readers can clearly relate the findings of the current study to existing evidence or previous studies and make better sense of the study. As it stands, the discussion looks more like the ‘results’. I further suggest that, if possible, the discussion be organised under themes for ease of understanding by readers.

**CONCLUSION**

**Remark(s):** Authors should make provision for contribution to knowledge and practice and also propose some clear recommendations for further research.

**OVERALL COMMENTS**

On the whole, the study was very well executed and presented. The authors have demonstrated a very knowledge of the subject matter and language. I commend the authors for a very good job done and suggest that they address the few remarks provided to improve the overall quality of the manuscript. The main issue is with the discussion. The authors need to reorganise this session so as the readers can make a better sense of the study.
