# Supplementary material for: Prediction models for post-discharge mortality among under-five children with suspected sepsis in Uganda: A multicohort analysis
Source: PLOS Glob Public Health. 2024 Apr 29;4(4):e0003050. doi: 10.1371/journal.pgph.0003050 (PMC11057737; doi:10.1371/journal.pgph.0003050)
Supplement: Attachment — Submitted filename: PDModel_PLOSgph_ResponseToReviewers_02Mar2024.docx [file pgph.0003050.s012.docx]

2^nd^ March 2024

Dear Dr Collins Otieno Asweto,

Re: **PGPH-D-24-00205**, Prediction models for post-discharge mortality among under-five children with suspected sepsis in Uganda: A multicohort analysis, PLOS Global Public Health

Please thank Dr Botha and Dr Likaka for reviewing our manuscript and for their thoughtful comments and helpful suggestions. We address each of their points below along with your journal requirements. Each point (journal requirement or reviewer’s comment) is presented on a light grey background; our response is underneath, with any revisions to the text marked in italics.

Some of these comments have prompted modifications to the manuscript text. A revised version has been submitted, with tracked changes along with a final version, as requested.

We hope that we have addressed the reviewers’ questions adequately and that you find our proposed revisions to the manuscript acceptable.

Sincerely,

**Dr Matthew O Wiens**, PharmD, PhD

Assistant Professor | Department of Anesthesiology, Pharmacology & Therapeutics | Institute for Global Health

The University of British Columbia | BC Children's Hospital

938 W 28th Ave | Vancouver BC | V5Z 4H4 Canada, Phone 604 997 7753, [matthew.wiens@bcchr.ca](mailto:matthew.wiens@bcchr.ca)

**Dr Jerome Kabakyenga**, MBChB, PhD

Professor | Maternal Newborn & Child Health Institute | Mbarara University of Science and Technology

P.O. Box 1410 | Mbarara, Uganda. Phone +256 77 259 0409 [jkabakyenga@must.ac.ug](mailto:jkabakyenga@must.ac.ug)

# Journal Requirements

1. Please review your reference list to ensure that it is complete and correct. If you have cited papers that have been retracted, please include the rationale for doing so in the manuscript text, or remove these references and replace them with relevant current references. Any changes to the reference list should be mentioned in the rebuttal letter that accompanies your revised manuscript. If you need to cite a retracted article, indicate the article’s retracted status in the References list and also include a citation and full reference for the retraction notice.

We have checked each of our references to ensure they are complete and correct. We amended minor citation details for existing references and added two additional references:

- Knappett et al. Pediatric post-discharge mortality in resource-poor countries: a systematic review and meta-analysis. EClinicalMedicine. 2024;67: 102380. doi:10.1016/j.eclinm.2023.102380 (addressing reviewer 1 comment on our Introduction)
- Janssen et al. Dealing with missing predictor values when applying clinical prediction models. Clin Chem. 2009;55: 994–1001. doi:10.1373/clinchem.2008.115345 (addressing reviewer 1 comment on our Discussion)
- Hoogland et al. Handling missing predictor values when validating and applying a prediction model to new patients. Stat Med. 2020;39: 3591–3607. doi:10.1002/sim.8682 (again, addressing reviewer 1 comment on our Discussion)

2. Please note that PLOS GPH has specific guidelines on code sharing for submissions in which author-generated code underpins the findings in the manuscript. In these cases, all author-generated code must be made available without restrictions upon publication of the work. Please review our guidelines at <https://journals.plos.org/globalpublichealth/s/materials-and-software-sharing#loc-sharing-code> and ensure that your code is shared in a way that follows best practice and facilitates reproducibility and reuse.

We have identified the code that we will release. It will be available openly, as described in our data sharing statement on p23 of the manuscript.

3. Please provide separate figure files in .tif or .eps format only and remove any figures embedded in your manuscript file. Please also ensure all files are under our size limit of 10MB.

For more information about figure files please see our guidelines:

<https://journals.plos.org/globalpublichealth/s/figures> 
<https://journals.plos.org/globalpublichealth/s/figures#loc-file-requirement>

We have provided all figures as .eps files. We noticed a small correction was required in the x-axis of one of the component graphs in figure 3. In correcting this, we have decided to separate the two halves of figure 3 into separate figures (now figure 3 and figure 4) as each is already a multi-component figure. We have renumbered figure citations in the manuscript and provided the updated legends.

4. We have noticed that you have uploaded Supporting Information files, but you have not included a list of legends. Please add a full list of legends for your Supporting Information files after the references list.

We apologize for missing this in our original submission. We have added a list of legends after the references – for our four figures (see above, we have split our original figure 3 into separate figure 3 and figure 4) and our one supporting information file, which includes a detailed contents list on its first two pages.

# Reviewers' comments

## Reviewer #1: BOTHA, Nkosi Nkosi

On the whole, the study was very well executed and presented. The authors have demonstrated a very knowledge of the subject matter and language. I commend the authors for a very good job done and suggest that they address the few remarks provided to improve the overall quality of the manuscript. The main issue is with the discussion.

Thank you for these comments. We address each of your separate points below.

**TOPIC**

Prediction models for post-discharge mortality among under-five children with suspected sepsis in Uganda: A multicohort analysis

**Remark(s) 1:** Reads fine.

Thank you.

**ABSTRACT**

**Introduction:** “Identifying those at risk has limited progress to improve outcomes.”

**Remark(s) 2, page 2, line 3:** This sentence does not read well. Recast, please.

**Methods:** Adequate.

**Results:** Adequate.

**Conclusion:** Adequate.

We have re-written this sentence:

*“**The lack of available tools to identify those at risk of post-discharge mortality has limited the ability to make progress towards improving outcomes.”*

**INTRODUCTION**

**Page 3, para 2, lines 9 & 10:** “Robust epidemiological data for pediatric post-discharge mortality in the context of sepsis and severe infection have been limited.”

**Remark(s) 3:** Provide reference for this statement.

We are now able to cite our recently published meta-analysis, which includes all post-discharge research including the few sepsis focused studies, and have added this reference to the manuscript:

- Knappett et al. Pediatric post-discharge mortality in resource-poor countries: a systematic review and meta-analysis. EClinicalMedicine. 2023;67:102380. doi: 10.1016/j.eclinm.2023.102380.

**MATERIALS AND METHODS**

**Protocol**

Detailed and rigorous

**RESULTS**

Adequately presented

Thank you.

**DISCUSSION**

**Remark(s):** While I credit the authors for a thorough presentation of this session, the session must be re-written completely. Typically, discussions are expected to highlight the main findings of the current study (not a mere repeat of the results) and show evidence of how these findings affirm or disaffirm existing evidence or previous studies. This way, readers can clearly relate the findings of the current study to existing evidence or previous studies and make better sense of the study. As it stands, the discussion looks more like the ‘results’. I further suggest that, if possible, the discussion be organised under themes for ease of understanding by readers.

Thank you for your careful review of our paper and your comments specifically related to the discussion. We agree that parts of the discussion were perhaps too focused on repeating some of our results. In particular, the 2^nd^ paragraph seems to inappropriately repeat some of our key findings. We have re-written this paragraph completely, removing several sentences and re-writing the remaining parts to focus specifically on the issue of (1) how our results fit into the existing literature on post-discharge risk and (2) how model deployment considerations, such as missingness of parameters is critically important for adoption and reach, and thus how having multiple models helps assuage such concerns. See p19 of the revised manuscript.

With regards to the remaining paragraphs, we do not feel we have re-stated our results.

- The 3^rd^ paragraph focuses on how prediction alone is insufficient to improve outcomes and we cite research to this effect. Of note, no studies have been conducted to address post-discharge mortality in the context of sepsis, thus we have no other interventional literature to draw on.
- The 4^th^ paragraph highlights the perennial issue of choosing model thresholds. What defines high vs low risk is context and resources. This issue is rarely addressed in the modelling literature, and we feel it’s critical to include such a discussion given the context where these kinds of models might be deployed.
- The 5^th^ paragraph relates to validation and the gradient that defines external vs internal validation. This is key given most models are never validated.
- The 6^th^ paragraph relates to digital health strategies within low-income country settings. Increasingly countries are implementing digital systems to facilitate patient care in both facility and community settings. As nations adopt such strategies, its importance (and potential) as it pertains to prediction cannot be understated.

These paragraphs form our discussion, and we feel they are quite relevant to the topic of prediction models in child health. We would welcome further discussion on this and are keen to do any further revisions on our discussion as it is deemed necessary by the editorial and review team.

**CONCLUSION**

**Remark(s):** Authors should make provision for contribution to knowledge and practice and also propose some clear recommendations for further research.

Thank you for this comment. We have re-written the conclusion highlighting the contribution to knowledge (development of models, reporting of post-discharge outcomes) and a call to further research (external validation, interventional research). See p22 of the revised manuscript.

**OVERALL COMMENTS**

On the whole, the study was very well executed and presented. The authors have demonstrated a very knowledge of the subject matter and language. I commend the authors for a very good job done and suggest that they address the few remarks provided to improve the overall quality of the manuscript. The main issue is with the discussion. The authors need to reorganise this session so as the readers can make a better sense of the study.

Thank you for the positive comments. See our response above to your comment about our Discussion.

## Reviewer #2: Dr Andrew Likaka

The study has been rigorous, following all research standards. The statistics are well thoughtful and easy to apply. Great findings too. Relevance to the study setting and the results can be generalized due to the methodology used.

Thank you for these positive comments.
