# Supplementary material for: Prediction models for post-discharge mortality among under-five children with suspected sepsis in Uganda: A multicohort analysis
Source: PLOS Glob Public Health. 2024 Apr 29;4(4):e0003050. doi: 10.1371/journal.pgph.0003050 (PMC11057737; doi:10.1371/journal.pgph.0003050)
Supplement: Attachment — Submitted filename: PGPH-D-24-00205_R1-3 word.docx [file pgph.0003050.s013.docx]

PLOS Global Public Health

## Prediction models for post-discharge mortality among under-five children with suspected sepsis in Uganda: A multicohort analysis

--Manuscript Draft--

| **Manuscript Number:** | PGPH-D-24-00205R1 |
| --- | --- |
| **Article Type:** | Research Article |
| **Full Title:** | Prediction models for post-discharge mortality among under-five children with suspected sepsis in Uganda: A multicohort analysis |
| **Short Title:** | Predicting post-discharge mortality in young children with sepsis |
| **Corresponding Author:** | Matthew O. Wiens  BC Children's Hospital  Vancouver, British Columbia CANADA |
| **Order of Authors:** | Matthew O Wiens, PhD |
|  | Vuong Nguyen, PhD |
|  | Jeffrey N Bone, MSc |
|  | Elias Kumbakumba, MBChB |
|  | Stephen Businge, MBChB |
|  | Abner Tagoola, MBChB |
|  | Sheila Oyella Sherine, MBChB |
|  | Emmanuel Byaruhanga, MBChB |
|  | Edward Ssemwanga, MBChB |
|  | Celestine Barigye, MBChB |
|  | Jesca Nsungwa, MBChB |
|  | Charles Olaro, MBChB |
|  | J Mark Ansermino, MBBCh |
|  | Niranjan Kissoon, MD |
|  | Joel Singer, PhD |
|  | Charles P Larson, MDCM |
|  | Pascal M Lavoie, MDCM |
|  | Dustin Dunsmuir, MSc |
|  | Peter Moschovis, MD |
|  | Stefanie Novakowski, PhD |
|  | Clare Komugisha, BSc |
|  | Mellon Tayebwa, MA |
|  | Douglas Mwesigwa, BA |
|  | Martina Knappett, BSc |
|  | Nicholas C West, MSc |
|  | Nathan Kenya Mugisha, MBChB |
|  | Jerome Kabakyenga, PhD |
| **Keywords:** | Global Health; Sepsis; Child, Hospitalized; Patient Discharge; Child Mortality; Models, Statistical |

| **Abstract:** | In many low-income countries, over five percent of hospitalized children die following hospital discharge. The lack of available tools to identify those at risk of post-discharge mortality has limited the ability to make progress towards improving outcomes. We aimed to develop algorithms designed to predict post-discharge mortality among children admitted with suspected sepsis. Four prospective cohort studies of children in two age groups (0-6 and 6-60 months) were conducted between 2012-2021 in six Ugandan hospitals. Prediction models were derived for six-months post-discharge mortality, based on candidate predictors collected at admission, each with a maximum of eight variables, and internally validated using 10-fold cross-validation. 8,810 children were enrolled: 470 (5.3%) died in hospital; 257 (7.7%) and 233 (4.8%) post-discharge deaths occurred in the 0-6-month and 6-60-month age groups, respectively. The primary models had an area under the receiver operating characteristic curve (AUROC) of 0.77 (95%CI 0.74-0.80) for 0-6-month-olds and 0.75 (95%CI 0.72-0.79) for  6-60-month-olds; mean AUROCs among the 10 cross-validation folds were 0.75 and 0.73, respectively. Calibration across risk strata was good: Brier scores were 0.07 and 0.04, respectively. The most important variables included anthropometry and oxygen saturation. Additional variables included: illness duration, jaundice-age interaction, and a bulging fontanelle among 0-6-month-olds; and prior admissions, coma score, temperature, age-respiratory rate interaction, and HIV status among 6-60-month-olds. Simple prediction models at admission with suspected sepsis can identify children at risk of post-discharge mortality. Further external validation is recommended for different contexts. Models can be digitally integrated into existing processes to improve peri-discharge care as children transition from the hospital to the community. |
| --- | --- |
| **Additional Information:** |  |
| **Question** | **Response** |
| **Financial Disclosure**  Enter a financial disclosure statement that describes the sources of funding for the work included in this submission and the role the funder(s) played. This includes grants and any commercial funding of the work or authors.  This statement will be typeset if the manuscript is accepted for publication.  *Please review the* [*submission guidelines*](http://journals.plos.org/globalpublichealth/s/submission-guidelines#loc-financial-disclosure-statement) *and the instructions link below for detailed requirements and guidance.* | The study was supported by funds from Grand Challenges Canada (MW; grant #TTS- 1809-1939, [https://www.grandchallenges.ca/),](http://www.grandchallenges.ca/)) Thrasher Research Fund (MW; grant #13878, [https://www.thrasherresearch.org/),](http://www.thrasherresearch.org/)) BC Children’s Hospital Foundation [(https://www.bcchf.ca/),](http://www.bcchf.ca/)) and Mining4Life (https://mining4life.org/). The funders had no role in study design, data collection and analysis, decision to publish, or preparation of the manuscript. |
| **Competing Interests**  On behalf of all authors, disclose any competing interests that could be perceived to bias this work.  This statement will be typeset if the manuscript is accepted for publication. | The authors have declared that no competing interests exist. |

| *Please review the instructions link below and PLOS Global Public Health’s* [*competing interests*](https://journals.plos.org/globalpublichealth/s/competing-interests) *policy to determine what information must be disclosed at submission.* |  |
| --- | --- |
| **Data Availability**  Before publication, Authors are required to make fully available and without restriction all data underlying their findings. Please see our [PLOS Data](https://journals.plos.org/globalpublichealth/s/data-availability) [Policy](https://journals.plos.org/globalpublichealth/s/data-availability) page for detailed information on this policy.  A **Data Availability Statement**, detailing where the data can be accessed, is required at first submission. Insert your Data Availability Statement in the box below.  Please see the [data reporting](https://journals.plos.org/globalpublichealth/s/submission-guidelines#loc-data-reporting) section of our submission guidelines for instructions on what you need to include in your Data Availability Statement.  This statement will be typeset if the manuscript is accepted for publication.  *PLOS allows rare exemptions to address legal and ethical concerns. If you have legal or ethical restrictions, please use the box below to detail these in full sentences for the Journal team to consider.* | Study materials including the study protocol, consent form, data collection tools, de- identified participant data, data dictionary, and the analysis code are available on request to the corresponding author (Matthew O. Wiens, matthew.wiens@bcchr.ca) or to the Institute for Global Health at BC Children’s and Women’s Hospital (Jessica Trawin, jessica.trawin@cw.bc.ca) or through the published protocol (https://doi.org/10.5683/SP3/QRUMNQ) and dataset (https://doi.org/10.5683/SP3/REPMSY). Owing to the sensitive nature of clinical data, access to the de-identified data is granted on a case-by-case basis and will require the signing of a data sharing agreement. |

<https://www.editorialmanager.com/pgph/download.aspx?id=310889&guid=9e124e32-82b0-4418-9f79-fe15e01bfa57&scheme=1>Manuscript	[Click here to](https://www.editorialmanager.com/pgph/download.aspx?id=310889&guid=9e124e32-82b0-4418-9f79-fe15e01bfa57&scheme=1)

[access/download;Manuscript;PDModel_PLOSgph_Manuscript_0](https://www.editorialmanager.com/pgph/download.aspx?id=310889&guid=9e124e32-82b0-4418-9f79-fe15e01bfa57&scheme=1)

1 Prediction models for post-discharge mortality among under-five
2 children with suspected sepsis in Uganda: A multicohort analysis

3

4 *Authors:* **Matthew O Wiens**^1,2,3,16^, PhD; **Vuong Nguyen**^1^, PhD; **Jeffrey N Bone**^3^, MSc; **Elias**
5 **Kumbakumba**^4^, MBChB; **Stephen Businge**^5^, MBChB; **Abner Tagoola**^6^, MBChB; **Sheila**
6 **Oyella Sherine**^7^, MBChB; **Emmanuel Byaruhanga**^8^, MBChB; **Edward Ssemwanga**^9^,
7 MBChB; **Celestine Barigye**^10^, MBChB; **Jesca Nsungwa**^11^, MBChB; **Charles Olaro**^11^, MBChB;
8 **J Mark Ansermino**^1,2,3^, MBBCh; **Niranjan Kissoon**^3,12^, MD; **Joel Singer**^13^, PhD; **Charles P**
9 **Larson**^14^, MDCM; **Pascal M Lavoie**^3,12^, MDCM; **Dustin Dunsmuir**^1,3^, MSc; **Peter P**
10 **Moschovis**^15^, MD; **Stefanie Novakowski**^1,2^, PhD; **Clare Komugisha**^16^, BSc; **Mellon**
11 **Tayebwa**^16^, MA; **Douglas Mwesigwa**^16^, BA; **Martina Knappett**^1^, BSc; **Nicholas West**^3^, MSc;
12 **Nathan Kenya Mugisha**^16^, MBChB; **Jerome Kabakyenga**^17,18^, PhD 13
14 *Affiliations:* **[1]** Institute for Global Health at BC Children’s and Women’s Hospital, 305-4088 Cambie
15 Street, Vancouver, BC V5Z 2X8, Canada; **[2]** Dept of Anesthesiology, Pharmacology & Therapeutics,
16 University of British Columbia, 217-2176 Health Sciences Mall, Vancouver, BC V6T 1Z3, Canada; **[3]**
17 BC Children’s Hospital Research Institute, 938 West 28th Ave, Vancouver, BC V5Z 4H4, Canada; **[4]**
18 Dept of Paediatrics and Child Health, Mbarara University of Science and Technology, Mbarara, Uganda;
19 **[5]** Holy Innocents Children's Hospital, PO Box 1685, Plot 6, Kaberebere-Insingiro Rd., Mbarara,
20 Uganda; **[6]** Jinja Regional Referral Hospital, P.O Box 43, Rotary Road, Jinja City, Uganda; **[7]** Masaka
21 Regional Referral Hospital, Alex Ssebowa, Masaka, Uganda; **[8]** Kawempe National Referral Hospital,
22 Kampala, Uganda; **[9]** Villa Maria Hospital, Masaka, Uganda; **[10]** Mbarara Regional Referral Hospital,
23 Mbarara, Uganda; **[11]** Ministry of Health for the Republic of Uganda, Kampala, Uganda; **[12]** Dept of
24 Pediatrics, University of British Columbia, Rm 2D19, 4480 Oak Street, BC Children’s Hospital,
25 Vancouver, BC V6H 3V4, Canada; **[13]** School of Population and Public Health, University of British
26 Columbia, 2206 East Mall, Vancouver, BC V6T 1Z3, Canada; **[14]** School of Population and Global
27 Health, McGill University, Suite 1200, 2001 McGill College Avenue, Montréal, Québec H3A 1G1,
28 Canada; **[15]** Division of Global Health, Massachusetts General Hospital, 125 Nashua Street, Suite 722,
29 Boston, MA 02114, Massachusetts, USA; **[16]** Walimu, Plot 5-7, Coral Crescent, Kololo, P .O. Box 9924,
30 Kampala, Uganda; **[17]** Maternal Newborn & Child Health Institute, Mbarara University of Science and
31 Technology, Mbarara, Uganda; **[18]** Dept of Community Health, Faculty of Medicine, Mbarara
32 University of Science and Technology, Mbarara, Uganda.

33

34	*Short title:* Predicting post-discharge mortality in young children with sepsis 35

36 *Corresponding authors:* **Matthew O. Wiens.** Institute for Global Health at BC Children’s and
37 Women’s Hospital, 305 - 4088 Cambie Street, Vancouver, BC, V5Z 2X8, Canada. tel: +1-604-
38 829-2562. email: [matthew.wiens@bcchr.ca](mailto:matthew.wiens@bcchr.ca); **Jerome Kabakyenga.** Mbarara University of
39 Science and Technology, Mbarara, Uganda. Tel: + 256-77-259-0409. email:
40 [jkabakyenga@must.ac.ug](mailto:jkabakyenga@must.ac.ug)

## 1 Abstract

2 In many low-income countries, over five percent of hospitalized children die following hospital
3 discharge. The lack of available tools to identify those at risk of post-discharge mortality has
4 limited the ability to make progress towards improving outcomes. We aimed to develop
5 algorithms designed to predict post-discharge mortality among children admitted with suspected
6 sepsis. Four prospective cohort studies of children in two age groups (0-6 and 6-60 months) were
7 conducted between 2012-2021 in six Ugandan hospitals. Prediction models were derived for six-
8 months post-discharge mortality, based on candidate predictors collected at admission, each with
9 a maximum of eight variables, and internally validated using 10-fold cross-validation. 8,810
10 children were enrolled: 470 (5.3%) died in hospital; 257 (7.7%) and 233 (4.8%) post-discharge
11 deaths occurred in the 0-6-month and 6-60-month age groups, respectively. The primary models
12 had an area under the receiver operating characteristic curve (AUROC) of 0.77 (95%CI 0.74-
13 0.80) for 0-6-month-olds and 0.75 (95%CI 0.72-0.79) for 6-60-month-olds; mean AUROCs
14 among the 10 cross-validation folds were 0.75 and 0.73, respectively. Calibration across risk
15 strata was good: Brier scores were 0.07 and 0.04, respectively. The most important variables
16 included anthropometry and oxygen saturation. Additional variables included: illness duration,
17 jaundice-age interaction, and a bulging fontanelle among 0-6-month-olds; and prior admissions,
18 coma score, temperature, age-respiratory rate interaction, and HIV status among 6-60-month-
19 olds. Simple prediction models at admission with suspected sepsis can identify children at risk of
20 post-discharge mortality. Further external validation is recommended for different contexts.
21 Models can be digitally integrated into existing processes to improve peri-discharge care as
22 children transition from the hospital to the community.

23

## 1 Introduction

2 Morbidity and mortality secondary to sepsis disproportionately affect children in low- and
3 middle-income countries, where >85% of global cases and deaths occur [1]. Lower income
4 regions are plagued by poorly resilient health systems, widespread socio-economic deprivation,
5 and unique vulnerabilities, including malnutrition. Reducing the overall sepsis burden requires a
6 multi-pronged strategy that addresses three periods along the care continuum – pre-facility,
7 facility and post-facility [2]. Of these, post-facility issues have been largely neglected in
8 research, policy, and practice [3].
9 Robust epidemiological data for pediatric post-discharge mortality in the context of sepsis and
10 severe infection have been limited [4]. Growing evidence points to a significant burden of post-
11 discharge mortality, which accounts for as many deaths as the acute hospital phase of illness
12 [5,6]. While comorbid conditions such as malnutrition and anemia have been linked to risk, other
13 factors such as illness severity (at admission and discharge), prior hospitalizations, and
14 underlying social vulnerability, are also independently associated with poor post-discharge
15 outcomes [7]. However, we lack simple data-driven methods to identify those at highest risk of
16 mortality.
17 Current epidemiological evidence has demonstrated critical gaps in care following discharge [8].
18 Most post-discharge deaths occur at home, rather than during a subsequent readmission,
19 indicating poor health utilization among the most vulnerable. Effective healthcare utilization is
20 often hampered by poverty, community and family social dynamics, and poorly linked and
21 unresponsive health facilities [9–11]. Providing quality care during and after discharge is a
22 significant challenge in many facilities, in part due to severely strained human and material
23 resources.
1 Effective solutions to improving the transition of care from hospital to home within poorly
2 resourced health systems must be child-centred and focused on identifying the most vulnerable
3 children [12]. In this study, we aim to update the development and validation of clinical
4 prediction models that identify children, admitted with suspected sepsis, who are at risk of post-
5 discharge mortality [13].

6

## 1 Materials and Methods

2 Study design and approvals
3 Four independently funded, prospective observational cohort studies were conducted with a
4 primary objective of generating model-building data: two among children under six months and
5 two among children 6-60 months of age. These studies were approved by the Mbarara University
6 of Science and Technology Research Ethics Committee (No. 05/11-11, 10-Nov-2011; and No.
7 15/10-16, 27-Jan-2017) and the University of British Columbia – Children’s and Women’s
8 Health Centre of BC Research Ethics Board (H10-01927, 01-Dec-2011; and H16-02679, 09-
9 May-2017). Written informed consent was obtained from the parent or legal guardian of all study
10 participants. This manuscript adheres to the Transparent Reporting of a multivariable prediction
11 model for Individual Prognosis Or Diagnosis (TRIPOD) statement [14].
12 Study setting and population
13 Subjects were enrolled from six hospitals in Uganda (**Supplementary-S1**). These facilities serve
14 catchments of 30 districts with a population of approximately 8.2 million individuals, including
15 approximately 1.4 million children under five years [15], in a mix of urban and rural areas,
16 reflecting a representative sampling of the Ugandan pediatric population.
17 All study cohorts had identical eligibility criteria. Any child admitted with suspected sepsis was
18 eligible. Suspected sepsis was defined as children admitted with a proven or suspected infection
19 (as determined by the treating medical team). We previously demonstrated that 90% of children
20 enrolled using these criteria meet the international pediatric sepsis consensus conference
21 (IPSCC) definition [16]. The IPSCC defines sepsis as the presence of the systemic inflammatory
22 response syndrome alongside a suspected or proven infection.
1 The first cohort (enrolment 13-Mar-2012 to 13-Jan-2014) was used previously to report a
2 predictive model for post-discharge mortality in 6-60-month-olds [13]. The second and third
3 cohorts were the primary enrolment for the present analysis, and were defined by age range: 0-6-
4 month-olds (enrolment 11-Jan-2018 to 30-Mar-2020) and 6-60-month-olds (enrolment 13-Jul-
5 2017 to 02-Jul-2019); these data have been previously reported [6]. The fourth cohort enrolled
6 only 0-6-month-olds (enrolment 31-Mar-2020 to 05-Aug-2021) in order to understand how the
7 early COVID-19 period impacted post-discharge outcomes. Protocols and procedures were
8 largely overlapping, and the same research staff were involved in data collection during all four
9 enrolment periods [17].
10 Data collection
11 Data collection tools are available through the Smart Discharges Dataverse [17]. Data collection
12 procedures were previously described (also **Supplementary-S1**) [6,13]. Briefly, trained study
13 nurses collected clinical, social, and demographic data from consented participants at hospital
14 admission; largely overlapping between the two age groups, some variables were specific to 0-6-
15 month-olds. These variables were our candidate predictors and were selected based on clinical
16 and contextual knowledge of possible factors relating to post-discharge mortality, using a
17 modified Delphi process to identify promising variables in each age group [18,19].
18 Study nurses recorded discharge diagnosis and status (died, discharged, discharged against
19 medical advice, referred). A field officer contacted enrolled children by phone two and four
20 months after discharge, with an in-person visit at six months to determine mortality status and, if
21 applicable, date of death. All data were collected using encrypted study tablets and uploaded to a
22 Research Electronic Data Capture (REDCap) database hosted at the BC Children’s Hospital
23 Research Institute (Vancouver, Canada) [20,21].
1 Model development
2 *Outcome definition and ascertainment*
3 The primary outcome of the prediction model was post-discharge mortality within six months of
4 discharge, analyzed as a binary outcome. While data were available to build a time-to-event
5 prediction model, time of death was considered irrelevant for modelling mortality. Complete six-
6 month follow-up data for vital status was available for 98% of our cohort.
7 Prediction model performance was evaluated primarily using area under the receiver operating
8 characteristic curve (AUROC). We also reported the precision-recall curve and area under the
9 precision-recall curve (PR-AUC), which are more appropriate for imbalanced datasets [22].
10 *Variable selection*
11 Recognising the challenges of implementing large prediction models in resource-constrained
12 settings, we determined *a priori* to develop three models for each age group and restricted each
13 model to eight variables drawing from a different pool of available predictors: one model
14 focused solely on commonly-available *clinical* variables; one model focused on commonly-
15 available *clinical and social* variables; and one model used *any* candidate predictor variable
16 (**Figure 1**). This approach aimed to reduce the impact of missingness in an implementation
17 scenario. A feature of our modelling approach (elastic net regression) was that final model size
18 could not be pre-specified, often resulting in large models. Therefore, we conducted two rounds
19 of variable selection.

20

**Fig 1. Variable selection for model development**

21

22 To prioritize parsimony, the first variable selection round reduced the list of possible predictors
23 to two subsets: one including only the most relevant *clinical* variables; and a second including
1 only the most relevant *clinical and social* variables. Variables included in these subsets were
2 determined *a priori*, based on clinical significance and ease of measurement in low-resource
3 settings*.* These subsets were used to derive ***intermediary models*** that were either clinically-
4 focused or clinically- and socially-focused; the full candidate predictor list for each age group
5 was also used to derive ***intermediary models*** that used any available variable (**Supplementary-**

#### 6 Tables S1.2, S1.3).

7 The second variable selection round involved ranking the importance of variables from each
8 intermediary model, which was calculated as the weighted sums of the absolute regression
9 coefficients [23]. The top eight unique variables (e.g., temperature and its quadratic term were
10 considered a single unique variable) were selected based on average ranking from 10-fold cross-
11 validation of the intermediary models. If an interaction term was ranked in the top eight
12 variables, both interaction terms were included. This second variable selection round produced a
13 family of ***final models*** to predict mortality within six months post-discharge (M6PD) that used
14 only the eight top-ranked variables in each age group: models using only ***clinical*** variables,
15 denoted by M6PD-C0-6 for 0-6-month-olds and M6PD-C6-60 for 6-60-month-olds; models using
16 ***clinical and social*** variables, denoted by M6PD-CS0-6 and M6PD-CS6-60; and models using ***any***
17 of available predictor variable, denoted by M6PD-A0-6 and M6PD-A6-60 (**Figure 1**).
18 Statistical analysis
19 The primary study sample size was determined to accomplish three aims: to explore the
20 epidemiology of post-discharge mortality, as previously reported;[6] to develop prediction
21 models; and as a control period for a later interventional phase. The estimated sample size was
22 determined as 2,117 and 1,551 for the 0-6-month and 6-60-month cohorts, respectively
1 (**Supplementary-S2**). All analyses were conducted using R version 4.2.2 (R Foundation for
2 Statistical Computing, Vienna, Austria) [24], reported in detail in **Supplementary-S2**.

3

## 1 Results

2 Study population
3 During the four enrolment periods, a total of 22,166 consecutively admitted children were
4 screened and 8,810 enrolled (**Figure 2**). Among 0-6-month-olds (n=3,665), 3,424 (93.4%)
5 survived to discharge. Complete 6-month outcomes were available for 3,349 (97.8%) of these
6 children, forming the full dataset for model development in this age group. Among 6-60-month-
7 olds (n=5,145), 4,916 (95.5%) survived to discharge. Complete 6-month outcomes were
8 available for 4,830 (98.2%) of these children, forming the full dataset for model development in
9 this age group.

10

**Fig 2. Study enrolment flow diagram**

11

12 Mortality within 6 months of discharge occurred in 257 (7.7%) 0-6-month-olds, with median
13 (interquartile range [IQR]) time to death of 31 (9-80) days, and in 233 (4.8%) 6-60-month-olds,
14 with time to death of 36 (11-105) days (**Supplementary**-**Figure S1.1**). Missing data were
15 minimal (**Table 1**).
16 These cohorts’ clinical and demographic details have been previously described (**Table 1**).[6,13]
17 The mean ±standard deviation [SD] age was 2.1 ±1.8 months with 1,884 (56.3%) male in the 0-
18 6-month group, and 21.7 ±13.7 months with 2,670 (55.3%) male in the 6-60-month group. Poor
19 growth/malnutrition was common, with 463 (13.8%) 0-6-month-olds and 668 (13.8%) 6-60-
20 month-olds classified as severely underweight (weight-for-age z-score <-3) and similar weight-
21 for-age z-score distributions in both age groups. Discharge diagnoses recorded by the clinical
22 team could be overlapping in the case of multiple diagnoses (**Supplementary-Table S1.4**). Most
23 predictor variables considered were associated with post-discharge mortality (**Table 1**).

1	**Table 1.** Demographics and univariable odds ratios for the risk of post-discharge infant mortality.

|  | **0-6 month (n = 3349)** | | | | **6-60 month (n = 4830)** | | | |
| --- | --- | --- | --- | --- | --- | --- | --- | --- |
| **Variable** | **n (%)/Mean (SD)** | **n Missing (%)** | **OR (95%CI)** | **P-value** | **n (%)/Mean (SD)** | **n Missing (%)** | **OR (95%CI)** | **P-value** |
| **A) Demographics** | | | | | | | | |
| **Sex, male** | 1884 (56.3%) | 0 (0%) | 1.18 (0.91, 1.53) | 0.218 | 2670 (55.3%) | 0 (0%) | 0.9 (0.69, 1.17) | 0.433 |
| **Age, months** | 2.1 (1.8) | 0 (0%) | 1.05 (0.98, 1.12) | 0.188 | 21.7 (13.7) | 1 (0.02%) | 1 (0.99, 1.01) | 0.471 |
| **B) Admission Anthropometry** | | | | | | | | |
| **BMI Z-scores** | -1 (2.2) | 5 (0.15%) | 0.78 (0.74, 0.82) | **<0.001** | -1 (9.8) | 32 (0.66%) | 0.86 (0.81, 0.91) | **<0.001** |
| ***< -3*** | 565 (16.9%) |  | 4.31 (3.24, 5.75) | **<0.001** | 775 (16%) |  | 2.31 (1.68, 3.16) | **<0.001** |
| ***-3 to -2*** | 399 (11.9%) |  | 2.36 (1.61, 3.40) | **<0.001** | 684 (14.2%) |  | 1.84 (1.28, 2.60) | **0.001** |
| ***> -2*** | 2380 (71.1%) |  | ref. | **<0.001** | 3339 (69.1%) |  | ref. | **<0.001** |
| **MUAC, mm a** | 113.7 (17.7) | 3 (0.09%) | 0.96 (0.96, 0.97) | **<0.001** | 139.2 (16.1) | 18 (0.37%) | 0.96 (0.95, 0.97) | **<0.001** |
| ***<110 / <115*** | 1304 (38.9%) |  | 3.81 (2.7, 5.51) | **<0.001** | 321 (6.6%) |  | 6.66 (4.76, 9.25) | **<0.001** |
| ***110-120 / 115-125*** | 942 (28.1%) |  | 1.58 (1.04, 2.42) | **0.033** | 514 (10.6%) |  | 2.77 (1.92, 3.92) | **<0.001** |
| ***>120 / >125*** | 1100 (32.8%) |  | ref. | **<0.001** | 3977 (82.3%) |  | ref. | **<0.001** |
| **Weight for age Z-scores** | -1.1 (2) | 2 (0.06%) | 0.71 (0.67, 0.75) | **<0.001** | -1.3 (1.7) | 12 (0.25%) | 0.71 (0.66, 0.76) | **<0.001** |
| ***< -3*** | 463 (13.8%) |  | 6.15 (4.58, 8.26) | **<0.001** | 668 (13.8%) |  | 4.58 (3.40, 6.17) | **<0.001** |
| ***-3 to -2*** | 356 (10.6%) |  | 3.61 (2.51, 5.14) | **<0.001** | 723 (15%) |  | 1.77 (1.20, 2.55) | **0.003** |
| ***> -2*** | 2528 (75.5%) |  | ref. | **<0.001** | 3427 (71%) |  | ref. | **<0.001** |
| **Weight for length Z-scores** | -1 (2.6) | 5 (0.15%) | 0.87 (0.84, 0.91) | **<0.001** | -1.2 (2) | 30 (0.62%) | 0.83 (0.78, 0.89) | **<0.001** |
| ***< -3*** | 627 (18.7%) |  | 2.52 (1.88, 3.35) | **<0.001** | 725 (15%) |  | 2.52 (1.83, 3.45) | **<0.001** |
| ***-3 to -2*** | 365 (10.9%) |  | 1.73 (1.16, 2.52) | **0.006** | 718 (14.9%) |  | 1.86 (1.30, 2.61) | **<0.001** |
| ***> -2*** | 2352 (70.2%) |  | ref. | **<0.001** | 3357 (69.5%) |  | ref. | **<0.001** |
| **C) Admission Clinical Assessment** | | | | | | | | |
| **How long ago since last admission** | 20 (0.6%) | | | | 20 (0.41%) | | | |
| ***Never*** | 2848 (85%) |  | ref. | **<0.001** | 2647 (54.8%) |  | ref. | **<0.001** |
| ***< 7days*** | 122 (3.6%) |  | 2.04 (1.12, 3.47) | **0.013** | 191 (4%) |  | 2.37 (1.34, 3.94) | **0.002** |
| ***7 days to <1 month*** | 180 (5.4%) |  | 2.68 (1.71, 4.06) | **<0.001** | 400 (8.3%) |  | 2.39 (1.60, 3.52) | **<0.001** |
| ***1 month to <1 year*** | 179 (5.3%) |  | 2.47 (1.56, 3.79) | **<0.001** | 1175 (24.3%) |  | 1.42 (1.03, 1.94) | **0.031** |
| ***≥1 year*** | 0 (0%) | | | | 397 (8.2%) |  | 0.50 (0.22, 0.97) | 0.06 |
| **SpO2** | 93.8 (6.8) | 9 (0.27%) | 0.96 (0.95, 0.98) | **<0.001** | 94.2 (6.5) | 22 (0.46%) | 0.95 (0.94, 0.97) | **<0.001** |
| ***< 90%*** | 598 (17.9%) |  | 1.78 (1.31, 2.41) | **<0.001** | 774 (16%) |  | 2.07 (1.49, 2.84) | **<0.001** |
| ***90% to 95%*** | 891 (26.6%) |  | 0.87 (0.62, 1.20) | 0.406 | 1236 (25.6%) |  | 1.15 (0.82, 1.58) | 0.404 |
| ***> 95%*** | 1851 (55.3%) |  | ref. | **<0.001** | 2798 (57.9%) |  | ref. | **<0.001** |
| **Heart rate, beats per minute** | 149.2 (23.6) | 3 (0.09%) | 1.00 (0.99, 1.00) | 0.276 | 144.8 (25.5) | 3 (0.06%) | 1.00 (0.99, 1.00) | 0.599 |

|  | **0-6 month (n = 3349)** | | | | **6-60 month (n = 4830)** | | | |
| --- | --- | --- | --- | --- | --- | --- | --- | --- |
| **Variable** | **n (%)/Mean (SD)** | **n Missing (%)** | **OR (95%CI)** | **P-value** | **n (%)/Mean (SD)** | **n Missing (%)** | **OR (95%CI)** | **P-value** |
| **Respiratory rate, breaths per minute** | 57.4 (17) | 5 (0.15%) | 1 (0.99, 1.01) | 0.875 | 48.1 (15.7) | 7 (0.14%) | 1.01 (1.00, 1.02) | **0.003** |
| **Systolic blood pressure, mmHg** | 85.1 (16.5) | 10 (0.3%) | 0.99 (0.99, 1.00) | 0.08 | 95.2 (13.4) | 8 (0.17%) | 0.99 (0.98, 1.00) | **0.028** |
| **Diastolic blood pressure, mmHg** | 46.3 (12.8) | 10 (0.3%) | 0.99 (0.98, 1.00) | 0.213 | 54.4 (11.6) | 8 (0.17%) | 0.99 (0.98, 1.00) | 0.079 |
| **Temperature, °C** | 37.4 (0.9) | 1 (0.03%) | 0.90 (0.78, 1.04) | 0.167 | 37.7 (1.2) | 3 (0.06%) | 0.81 (0.72, 0.91) | **<0.001** |
| ***< 36.5*** | 386 (11.5%) |  | 0.96 (0.62, 1.43) | 0.835 | 505 (10.5%) |  | 1.28 (0.84, 1.89) | 0.234 |
| ***36.5 to 37.5*** | 1699 (50.7%) |  | ref. | 0.581 | 1868 (38.7%) |  | ref. | **0.014** |
| ***37.6 to 39*** | 1072 (32%) |  | 1.01 (0.76, 1.34) | 0.923 | 1638 (33.9%) |  | 0.82 (0.6, 1.12) | 0.222 |
| ***> 39*** | 191 (5.7%) |  | 0.65 (0.32, 1.20) | 0.202 | 816 (16.9%) |  | 0.58 (0.37, 0.89) | **0.016** |
| **Abnormal BCS score** | 285 (8.5%) | 0 (0%) | 2.37 (1.64, 3.34) | **<0.001** | 408 (8.4%) | 0 (0%) | 1.93 (1.30, 2.78) | **0.001** |
| **Malaria test positive** | 324 (9.7%) | 1 (0.03%) | 0.56 (0.31, 0.92) | **0.032** | 1480 (30.6%) | 11 (0.23%) | 0.76 (0.56, 1.02) | 0.075 |
| **HIV+** | 119 (3.6%) | 2 (0.06%) | 1.37 (0.70, 2.42) | 0.317 | 144 (3%) | 22 (0.46%) | 3.81 (2.31, 6.00) | **<0.001** |
| **Haemoglobin, g/dL** | 13 (3.3) | 4 (0.12%) | 0.96 (0.92, 1.00) | **0.036** | 10.4 (3.2) | 608 (12.59%) b | 0.88 (0.85, 0.92) | **<0.001** |
| ***No anaemia*** | 2435 (72.7%) |  | ref. | **0.003** | 1983 (41.1%) |  | ref. | **<0.001** |
| ***Mild anaemia*** | 788 (23.5%) |  | 1.29 (0.96, 1.72) | 0.091 | 1535 (31.8%) |  | 1.59 (1.15, 2.21) | **0.006** |
| ***Severe anaemia*** | 122 (3.6%) |  | 2.47 (1.44, 4.04) | **0.001** | 704 (14.6%) |  | 2.67 (1.87, 3.82) | **<0.001** |
| **D) Maternal and Social Characteristics** | | | | | | | | |
| **Time it took to reach hospital** | 0 (0%) | | | | 1 (0.02%) | | | |
| ***<30 minutes*** | 806 (24.1%) |  | ref. | **<0.001** | 1015 (21%) |  | ref. | **<0.001** |
| ***30 minutes to <1 hour*** | 1224 (36.5%) |  | 1.15 (0.77, 1.75) | 0.498 | 1519 (31.4%) |  | 1.67 (1.04, 2.75) | **0.037** |
| ***≥1 hour*** | 1319 (39.4%) |  | 2.65 (1.86, 3.88) | **<0.001** | 2295 (47.5%) |  | 2.89 (1.90, 4.58) | **<0.001** |
| **Maternal age, years** | 26.3 (5.7) | 50 (1.49%) | 1.00 (0.98, 1.02) | 0.871 | 27.9 (6.4) | 167 (3.46%) | 1.00 (0.98, 1.02) | 0.899 |
| **Number of children** | 2.8 (1.8) | 1 (0.03%) | 1.01 (0.94, 1.08) | 0.87 | 3.2 (2.1) | 3 (0.06%) | 1.04 (0.98, 1.10) | 0.228 |
| **Had a child who died previously** | 577 (17.2%) | 1 (0.03%) | 1.21 (0.87, 1.65) | 0.249 | 1066 (22.1%) | 3 (0.06%) | 1.27 (0.93, 1.70) | 0.123 |
| **Maternal education** | 16 (0.48%) | | | | 49 (1.01%) | | | |
| ***No school*** | 105 (3.1%) |  | ref. | **0.001** | 334 (6.9%) |  | ref. | **<0.001** |
| ***≤P3*** | 207 (6.2%) |  | 1.16 (0.57, 2.48) | 0.684 | 345 (7.1%) |  | 1.30 (0.70, 2.43) | 0.411 |
| ***P4-P7*** | 1327 (39.6%) |  | 0.71 (0.39, 1.41) | 0.297 | 2088 (43.2%) |  | 0.98 (0.61, 1.65) | 0.922 |
| ***S1-S6*** | 1175 (35.1%) |  | 0.58 (0.32, 1.16) | 0.098 | 1517 (31.4%) |  | 0.61 (0.36, 1.07) | 0.073 |
| ***Post-Secondary*** | 519 (15.5%) |  | 0.36 (0.18, 0.77) | **0.006** | 497 (10.3%) |  | 0.41 (0.19, 0.85) | **0.018** |
| **Maternal HIV** | 1 (0.03%) | | | | 6 (0.12%) | | | |
| ***No*** | 3052 (91.1%) |  | ref. | 0.125 | 3915 (81.1%) |  | ref. | **0.012** |
| ***Yes*** | 246 (7.3%) |  | 1.48 (0.95, 2.24) | 0.071 | 432 (8.9%) |  | 1.63 (1.07, 2.40) | **0.016** |
| ***Unknown*** | 50 (1.5%) |  | 1.71 (0.65, 3.77) | 0.222 | 477 (9.9%) |  | 1.57 (1.05, 2.29) | **0.023** |

|  | **0-6 month (n = 3349)** | | | | **6-60 month (n = 4830)** | | | |
| --- | --- | --- | --- | --- | --- | --- | --- | --- |
| **Variable** | **n (%)/Mean (SD)** | **n Missing (%)** | **OR (95%CI)** | **P-value** | **n (%)/Mean (SD)** | **n Missing (%)** | **OR (95%CI)** | **P-value** |
| **Bed net use** | 1 (0.03%) | | | | 3 (0.06%) | | | |
| ***Never*** | 2809 (83.9%) |  | 0.85 (0.44, 1.57) | 0.611 | 3630 (75.2%) |  | 1.05 (0.64, 1.73) | 0.841 |
| ***Sometimes*** | 337 (10.1%) |  | ref. | 0.544 | 631 (13.1%) |  | ref. | 0.443 |
| ***Always*** | 202 (6%) |  | 0.80 (0.55, 1.20) | 0.262 | 566 (11.7%) |  | 0.85 (0.59, 1.26) | 0.389 |
| **Water source** | 0 (0%) | | | | 3 (0.06%) | | | |
| ***Bore hole*** | 655 (19.6%) |  | 1.72 (1.22, 2.4) | **0.002** | 1042 (21.6%) |  | 2.61 (1.84, 3.72) | **<0.001** |
| ***Fast running water*** | 21 (0.6%) |  | 0.84 (0.05, 4.08) | 0.862 | 515 (10.7%) |  | 1.76 (1.08, 2.80) | **0.019** |
| ***Municipal water*** | 1630 (48.7%) |  | ref. | **<0.001** | 1981 (41%) |  | ref. | **<0.001** |
| ***Open source*** | 541 (16.2%) |  | 2.12 (1.51, 2.98) | **<0.001** | 558 (11.6%) |  | 1.88 (1.19, 2.93) | **0.006** |
| ***Protected spring*** | 392 (11.7%) |  | 1.49 (0.97, 2.23) | 0.063 | 590 (12.2%) |  | 2.03 (1.30, 3.11) | **0.001** |
| ***Slow running water*** | 110 (3.3%) |  | 1.67 (0.80, 3.16) | 0.14 | 141 (2.9%) |  | 1.99 (0.86, 4.03) | 0.075 |
| **Boil/disinfect/filter water** | 2526 (75.4%) | 0 (0%) | 0.84 (0.64, 1.13) | 0.237 | 3402 (70.4%) | 2 (0.04%) | 0.51 (0.39, 0.67) | **<0.001** |
| **E) Discharge Characteristics** | | | | | | | | |
| **Length of stay, days** | 5.6 (4.4) | 0 (0%) |  |  | 5.1 (8.2) | 0 (0%) |  |  |
| **Discharge status** | 2 (0.06%) | | | | 0 (0%) | | | |
| ***Referred to higher level of care*** | 164 (4.9%) | | | | 101 (2.1%) | | | |
| ***Routine discharge*** | 2810 (83.9%) | | | | 4143 (85.8%) | | | |
| ***Unplanned discharge*** | 373 (11.1%) | | | | 586 (12.1%) | | | |
| **F) Variables collected only for 0-6-month** | | | | | | | | |
| **Abdominal distension** | 217 (6.5%) | 2 (0.06%) | 1.79 (1.15, 2.70) | **0.007** |  | | | |
| **Antenatal visits** | 4.9 (1) | 48 (1.43%) | 0.89 (0.78, 1.01) | 0.066 |  |  |  |  |
| **Dehydration, WHO categories** | 11 (0.33%) | | | |  |  |  |  |
| ***No dehydration*** | 2844 (84.9%) |  | ref. | **<0.001** |  |  |  |  |
| ***Some dehydration*** | 399 (11.9%) |  | 1.64 (1.15, 2.30) | **0.005** |  |  |  |  |
| ***Severe dehydration*** | 95 (2.8%) |  | 3.40 (1.96, 5.62) | **<0.001** |  |  |  |  |
| **Delivery method, caesarean** | 497 (14.8%) | 4 (0.12%) | 0.74 (0.49, 1.08) | 0.136 |  |  |  |  |
| **Duration of present illness** | 4 (0.12%) | | | |  |  |  |  |
| ***<48 hours*** | 957 (28.6%) |  | ref. | **<0.001** |  |  |  |  |
| ***48 hours to 7 days*** | 60 (1.8%) |  | 1.46 (1.05, 2.06) | **0.026** |  |  |  |  |
| ***8 days to 1 month*** | 1985 (59.3%) |  | 3.16 (2.09, 4.80) | **<0.001** |  |  |  |  |
| ***>1 month*** | 343 (10.2%) |  | 5.13 (2.52, 9.87) | **<0.001** |  |  |  |  |
| **Fontanelle** | 132 (3.9%) | 6 (0.18%) | 2.4 (1.44, 3.81) | **<0.001** |  |  |  |  |
| **Glucose, mmol/L** | 5.7 (2.5) | 2 (0.06%) | 1.03 (0.98, 1.08) | 0.188 |  |  |  |  |

|  | **0-6 month (n = 3349)** | **6-60 month (n = 4830)** |
| --- | --- | --- |
| **Variable** | **n (%)/Mean (SD) n Missing (%) OR (95%CI) P-value** | **n (%)/Mean (SD) n Missing (%) OR (95%CI) P-value** |
| **Not previously tested for HIV** | 2968 (88.6%) 0 (0%) 0.93 (0.64, 1.40) 0.719 |  |
| **Referral visit** | 1056 (31.5%) 1 (0.03%) 1.70 (1.31, 2.20) **<0.001** |  |
| **Neonatal jaundice** | 261 (7.8%) 34 (1.02%) 1.31 (0.83, 1.99) 0.219 |  |
| **Lactate level, mmol/L** | 2.5 (1.6) 9 (0.27%) 1.10 (1.03, 1.18) **0.003** |  |
| **Mother currently acutely ill** | 132 (3.9%) 13 (0.39%) 0.56 (0.22, 1.18) 0.171 |  |
| **Mother has chronic illness** | 251 (7.5%) 19 (0.57%) 1.29 (0.81, 1.97) 0.256 |  |
| **Child less than 30 days old** | 1353 (40.4%) 2 (0.06%) 0.68 (0.52, 0.89) **0.006** |  |
| **Pallor** | 307 (9.2%) 2 (0.06%) 2.15 (1.50, 3.03) **<0.001** |  |
| **Premature birth** | 210 (6.3%) 6 (0.18%) 2.05 (1.33, 3.06) **0.001** |  |
| **Prior care sought for current illness** | 1995 (59.6%) 0 (0%) 1.82 (1.38, 2.42) **<0.001** |  |
| **Sucking well when breastfeeding, or** | 1956 (58.4%) 8 (0.24%) 0.47 (0.36, 0.61) **<0.001** |  |
| **feeding well if not breastfed** |  |  |
| **Sucking well when breastfeeding, or** |  |  |
| **feeding well if not breastfed, prior to** | 2589 (77.3%) 392 (11.7%) 0.59 (0.42, 0.85) **0.004** |  |
| **illness** |  |  |
| **When did the baby cry after birth** | 97 (2.9%) |  |
| ***Immediately*** | 2805 (83.8%) ref. **0.041** |  |
| ***<5 minutes*** | 138 (4.1%) 1.83 (1.04, 3.02) **0.025** |  |
| ***5 to 10 minutes*** | 141 (4.2%) 1.32 (0.70, 2.30) 0.351 |  |
| ***11 to 30 minutes*** | 68 (2%) 1.26 (0.48, 2.72) 0.594 |  |
| ***>30 minutes*** | 100 (3%) 2.12 (1.14, 3.68) **0.011** |  |
| **Abnormal tone** | 285 (8.5%) 2 (0.06%) 3.11 (2.21, 4.31) **<0.001** |  |
| **Decreased urine production** | 677 (20.2%) 99 (2.96%) 1.91 (1.43, 2.52) **<0.001** |  |

1 For non-binary categorical variables, the p-value for the reference group (labelled ref.) indicates the global p-value. Odds ratios and p-values were not calculated
2 for discharge variables.
3 a MUAC thresholds given for 0-6-month / 6-60-month cohorts
4 b High prevalence of missing data for hemoglobin due to faulty capillary tubes during data collection
5 Abbreviations: BCS = Blantyre coma scale; BMI = body mass index; HIV+ = human immunodeficiency virus positive; MUAC = mid-upper arm circumference;
6 OR = odds ratio; SpO2 = oxygen saturation; WHO = World Health Organization

7

1 Prediction models
2 The intermediary variable models were large (coefficients, performance metrics, and variable
3 importance reported in **Supplementary S3-S5**). The models derived using all candidate
4 predictors (intermediary *any* variable model) included 41 unique variables in the 0-6-month
5 model and 19 unique variables in the 6-60-month model. Applied to the entire dataset for each
6 age group, the AUROC was 0.81 (95%CI 0.79 to 0.84) for the 0-6-month model and 0.79
7 (95%CI 0.77 to 0.82) for the 6-60-month model, with average AUROCs of 0.77 (range 0.69-
8 0.87) and 0.76 (range 0.71-0.81) across the 10 cross-validations, respectively; the PR-AUC was
9 0.27 for the 0-6-month model and 0.18 for the 6-60-month model, with average PR-AUCs of
10 0.22 (range 0.13-0.31) and 0.16 (range 0.11-0.21) across the 10 cross-validations, respectively.
11 Calibration was good at low predicted probabilities, with a Brier scores of 0.07 (range 0.06-0.07)
12 for the 0-6-month model and 0.04 (range 0.04-0.05) for the 6-60-month model. Calibration
13 decreased at higher predicted probabilities, although there were almost no individuals with
14 probabilities >40%. In both age groups, mid-upper arm circumference (MUAC) was identified as
15 the variable with the highest importance.
16 The final models are summarized in **Table 2**, and detailed in **Supplementary S6-S8**, including
17 all model terms, their coefficients, and plots outlining the relative importance of coefficients in
18 each model.

1 **Table 2.** Summary of performance and variables included in the set of final models with reduced number
2 of variables using the probability threshold that gave a sensitivity of 0.8.

| **A) 0-6-month models** | **M6PD-C0-6** | **M6PD-CS0-6 *** | **M6PD-A0-6 *** |
| --- | --- | --- | --- |
| **Average Cross-Validation Performance** |  |  |  |
| Specificity | 0.60 | 0.61 | 0.61 |
| AUROC | 0.75 | 0.76 | 0.76 |
| PPV | 0.15 | 0.16 | 0.16 |
| NPV | 0.97 | 0.97 | 0.97 |
| PRAUC | 0.23 | 0.23 | 0.23 |
| Brier Score | 0.07 | 0.07 | 0.07 |
| **Full Dataset Performance**  Specificity | 0.58 | 0.62 | 0.62 |
| AUROC | 0.77 | 0.77 | 0.77 |
| PPV | 0.14 | 0.15 | 0.15 |
| NPV | 0.97 | 0.97 | 0.97 |
| PRAUC | 0.23 | 0.22 | 0.22 |
| Brier Score | 0.07 | 0.07 | 0.07 |
| **Variables**  Age, months | ✓ ✓ ✓ | | |
| Duration of present illness, categorical | ✓ ✓ ✓ | | |
| MUAC, mm | ✓ ✓ ✓ | | |
| Neonatal jaundice, binary | ✓ ✓ ✓ | | |
| Sucking well when breastfeeding, binary | ✓ ✓ ✓ | | |
| SpO2, % | ✓ ✓ ✓ | | |
| Time to reach hospital, categorical | ✓ ✓ | | |
| Weight for age z-score | ✓ ✓ ✓ | | |
| Fontanelle, binary | ✓ | | |
| **B) 6-60-month models** | **M6PD-C6-60 M6PD-CS6-60 M6PD-A6-60** | | |
| **Average Cross-Validation Performance**  Specificity | 0.57 0.59 0.54 | | |
| AUROC | 0.73 0.74 0.75 | | |
| PPV | 0.09 0.09 0.08 | | |
| NPV | 0.98 0.98 0.98 | | |
| PRAUC | 0.16 0.15 0.15 | | |
| Brier Score | 0.04 0.04 0.04 | | |
| **Full Dataset Performance**  Specificity | 0.53 | 0.58 | 0.55 |
| AUROC | 0.75 | 0.76 | 0.77 |
| PPV | 0.08 | 0.09 | 0.08 |
| NPV | 0.98 | 0.98 | 0.98 |
| PRAUC | 0.17 | 0.16 | 0.17 |
| Brier Score | 0.04 | 0.04 | 0.04 |
| **Variables**  Age, months | ✓ ✓ ✓ | | |
| Haemoglobin, g/dl | ✓ | | |
| HIV, binary | ✓ ✓ | | |
| How long since last admission, categorical | ✓ ✓ ✓ | | |
| MUAC, mm | ✓ ✓ ✓ | | |
| SpO2, % | ✓ ✓ ✓ | | |

| Water source, categorical | ✓ ✓ |
| --- | --- |
| Weight for age z-score | ✓ ✓ ✓ |
| Abnormal BCS, binary | ✓ |
| Respiratory rate, bpm | ✓ |
| Temperature, °C | ✓ |
| Boil/disinfect/filter water | ✓ |

1 * Note, the 0-6-month final reduced (M6PD-A0-6) and final clinical and social model (M6PD-CS0-6) are identical
2 since the same variables were selected.
3 Abbreviations: AUROC = area under the receiver operating curve; BCS = Blantyre coma scale; bpm = breaths per
4 minute; HIV, human immunodeficiency virus; MUAC = mid-upper arm circumference; NPV = negative predictive
5 value; PPV = positive predictive value; PRAUC = area under the precision-recall curve; SpO2 = oxygen saturation

6

7 Final 0-6-month models
8 The M6PD-C0-6 model, using only simple *clinical* variables, included weight-for-age z-score
9 (mean rank [rm] =1.4, selection frequency [sf] =10), MUAC (rm=1.6, sf=10), feeding status
10 (rm=3.4, sf=10), SpO2 (rm=5.8, sf=9), duration of illness (rm=6.2, sf=9), age × jaundice (rm=7.8,
11 sf=7), and bulging fontanelle (rm=8.3, sf=8) (**Supplementary-Table S3.1)**. The AUROC was
12 0.77 (95%CI 0.74 to 0.80) and PR-AUC was 0.23 when applied to the entire 0-6-month dataset
13 (**Figure 3**), while the average AUROC and PR-AUC across the internal 10 cross-validations
14 were 0.75 (range 0.63-0.85) and 0.23 (range 0.11-0.33), respectively (**Table 2A;**
15 **Supplementary-S6**). Setting the sensitivity to 80%, the corresponding probability threshold was
16 0.058; at this threshold, positive and negative predictive values were 14% and 97%, respectively.
17 Calibration at low predicted probabilities was good, with a Brier score of 0.07 (**Figure 3,**
18 **Supplementary-Figure S6.3**). Calibration at probabilities beyond 30-40% was poor, but sample
19 sizes were very small in this range.

20

**Fig 3. Performance of the final clinical model for 0-6 months (M6PD-C0-6) on the full dataset.** The points on the receiver operating characteristic (ROC), precision recall (PR), and gain curve plots indicate co-ordinates for the probability threshold at sensitivity=80%, with positive predictive value (PPV) and negative predictive value (NPV) also reported at this threshold.

21

22

23

24 The M6PD-CS0-6 model, using *social and clinical* variables, was nearly identical in performance
25 to M6PD-C0-6; the variables were largely overlapping with only fontanelle status replaced by
1 travel time required to reach hospital (**Table 2A; Supplementary-S7**). M6PD-A0-6 that used *any*
2 available variable, was identical to M6PD-CS0-6 (**Table 2A; Supplementary-S8**).
3 Final 6-60-month models
4 The M6PD-C6-60 model, using only *clinical* predictors, included nine variables (the 8^th^ best-
5 performing variable included an interaction with a new variable; **Supplementary-Table S3.2**):
6 MUAC (rm=1, sf=10), SpO2 (rm=2.7, sf=10), weight-for-age z-score (rm=2.8, sf=10), time since
7 prior admission (rm=4.7, sf=10), abnormal coma score (rm=5.8, sf=9), temperature (rm=6.4, sf=9),
8 HIV status (rm=6.5, sf=9) and age × respiratory rate (rm=9.1, sf=2). The AUROC was 0.74
9 (95%CI 0.72 to 0.79) and PR-AUC was 0.17 when applied to the entire 6-60-month dataset
10 (**Figure 4**), with an average AUROC of 0.73 (range 0.67-0.77) and average PR-AUC of 0.16
11 (range 0.10-0.19) across the 10 cross-validations (**Table 2B; Supplementary-S6**). Setting
12 sensitivity to 80%, the corresponding probability threshold was 0.036; at this threshold, positive
13 and negative predictive values were 0.08 and 0.98, respectively. Calibration across risk strata
14 was good with a Brier score of 0.04 (**Figure 4, Supplementary-Figure S6.4**).

15

**Fig 4. Performance of the final clinical model for 6-60 months (M6PD-C6-60) on the full dataset.** The points on the receiver operating characteristic (ROC), precision recall (PR), and gain curve plots indicate co-ordinates for the probability threshold at sensitivity=80%, with positive predictive value (PPV) and negative predictive value (NPV) also reported at this threshold.

16

17

18

19 The M6PD-CS6-60 model, which used *clinical and social* variables, was almost identical to
20 M6PD-C6-60, with only home water source and water disinfection practices replacing coma score
21 (**Table 2B; Supplementary-S7**). M6PD-A6-60 was similar to M6PD-CS6-60, with water
22 disinfection practices replaced by hemoglobin; performance metrics were nearly identical (**Table**

#### 23 2B; Supplementary-S8).

24

## 1 Discussion

2 Using four large, objective-driven, prospective cohorts of under-5 children admitted with
3 suspected sepsis, we derived and internally-validated prediction models for post-discharge
4 mortality using only admission data. Their performance to predict mortality up to six months
5 post-discharge was good, suggesting potential utility to improve post-discharge outcomes by
6 linking individual risk to interventional intensity [25]. Data-driven, child-centred approaches to
7 post-discharge care have been strongly advocated for [5,26,27]. Our robust, cross-validated
8 models utilized data from multiple sites, captured over eight years, and should spur focus on
9 external validation outside Uganda.
10 Several recent studies have shown that post-discharge mortality can be closely linked to a variety
11 of key risk factors, such as malnutrition and disease severity [4–6]. Our results affirm this
12 through formal model development using varied sets of few, objective, and easy-to-collect
13 variables typically available in most settings where such models would be used. In a model
14 deployment context, however, the general approach of developing a single model may not
15 always be sufficient since missingness at the point-of-care may be common. Having multiple
16 simplified models with similar performance, as we saw in our models, may help alleviate these
17 kinds of logistical barriers to implementation [28,29].
18 Without an effective intervention, risk prediction has limited utility. Understanding discharge as
19 a dynamic process encompassing the time between admission and re-integration into community
20 care is integral to our focus on admission factors [30]. Early identification allows post-discharge
21 risk to be incorporated into discharge planning from the outset. Significant challenges in
22 preparing caregivers for discharge and the transition home have been identified, suggesting that
23 early planning is an essential component of effective peri-discharge care [30].
1 Choosing risk probability thresholds to classify post-discharge mortality as a binary outcome
2 depends on many factors, including availability of human resources, baseline risk, risk tolerance,
3 and impact on patients/caregivers. Though the thresholds chosen may prove useful in some
4 settings, choice of both the threshold and number of thresholds must be informed by local
5 context and constitutes a critically important consideration for deployment of this, or any, risk
6 model [31].
7 Although internal validation can justify using models within the region in which they are
8 derived, external validation using different data sources (ideally several) from different regions is
9 essential [32], using both existing and future data [5]. Consequently, we have several prospective
10 studies underway, and will establish data sharing agreements with other collaborators to enable
11 use of their collected data. However, not every conceivable implementation region for any given
12 model can be subjected to external validation. A more pragmatic approach is developing a
13 region-specific model-updating process, integrated over the life-course of the model. Calibration
14 drift due to secular trends, the measured impact of the model itself, and peculiarities of each
15 individual site are key considerations in model deployment [33]. Digitization of the healthcare
16 system will help establishing these processes [34].
17 As health systems in low-income countries increasingly adopt electronic health records,
18 incorporating algorithms to augment care decisions has tremendous potential to improve
19 outcomes and facilitate adoption of these digital systems [35,36]. Using routinely-collected
20 variables can allow models to run without additional user input and automatically prompt follow-
21 up guidance to the medical team and patient, encouraging adoption and linkage to interventional
22 programs. Furthermore, such systems can report baseline risk data and, when linked to follow-up
23 programs, data on readmission and mortality to national-level health management information
1 systems, such as DHIS2 [37]. These data can be used in model calibration and updating, ensuring
2 site-specific validity. Contextually-validated digital clinical decision support systems utilizing
3 risk algorithms are increasingly recognized as essential to achieving universal health coverage,
4 especially in low- and middle-income countries [38,39].
5 Limitations
6 This study has several limitations. While our models performed well with internal cross-
7 validation, demonstrating good performance in planned external validation is essential to
8 encourage adoption. Second, our models do not accommodate missing data for predictor
9 variables. While missing data rates were very low, this is unlikely to represent true rates of
10 missingness in real-world practice. We developed a family of models, varying in number and
11 type of predictors, which produced similar performance, to partially address this limitation.
12 Future research will explore more robust methods for addressing missing data, including
13 building sub-models to allow for every possible combination of missing variable [29]. Third,
14 these models were developed in the absence of a proven program to utilize a risk-based approach
15 to care, limiting their current utility. While merely knowledge of individual risk can change
16 behaviour and may influence provision of peri-discharge care, risk-informed approaches to
17 follow-up care are also currently under investigation [40]. Fourth, calibration was good at most
18 observed risk levels, but there were very few patients with predicted risk greater than 40-50%, so
19 calibration beyond these probabilities could not be assessed. Regardless, our models should
20 perform adequately for implementation purposes using the optimal threshold cut-offs identified.
21 Finally, the added value of these models may be questioned in the light of previously published
22 models [40–42]. Our models were based on purposively built cohorts, with *a priori* stakeholder
23 engagement regarding relevant variables and their measurement timing, and were uniquely
1 developed within the clinical rubric of suspected sepsis, which is increasingly recognized as a
2 global health priority.
3 Conclusion
4 Post-discharge mortality in the context of suspected sepsis occurs frequently in children under
5 five years old, but those at highest risk can be identified using simple clinical criteria, measured
6 at admission. Being able to select from a range of prediction models, with similar performance
7 parameters, may support wider implementation of digital risk-stratification tools in different
8 clinical settings. Future work must focus on both external validation as well evaluation of how
9 risk-stratified care can improve post-discharge outcomes.

10

## 1 Acknowledgements

2 We would like to acknowledge all past and present members of the Smart Discharges Research
3 program for their efforts in data collection, administration, logistics support, and all study
4 activities, including but not limited to: Tumwebaze Godfrey, Agaba Collins, Tumukunde Goreth,
5 Naturinda Mackline, Assimwe Abibu, Nakafero Joan, Kiiza Israel, Kitenda Julius, Kamba Ayub,
6 Kuguminkiriza Brenda, Kabajasi Olive, Kembabazi Brenda, Happy Annet, Tusingwire Fredson,
7 Nuwasasira Agaston, Ankatse Christine, Naturinda Rabecca, Nabawanuka Abbey Onyachi,
8 Kamazima Justine, Kairangwa Racheal, Ounyesiga Thomas, Mwoya Yuma, Twebaze Florence,
9 Bulage Mary, Tugumenawe Darius, Tuhame Dyonisius, Twesigye Leonidas, Kamusiime Olivia,
10 Ainembabazi Harriet, Abaho Samuel, Nakabiri Zaituni, Naigaga Shaminah, Kisame Zorah,
11 Babirye Clare, Kayegi Maliza, Opuko Wilson, Mwaka Savio, Baryahirwa Hassan, Mutungi
12 Alexander, Charlene Kanyali, Catherine Kiggundu, Alexia Krepiakevich, Brooklyn Nemetchek,
13 Jessica Trawin, Maryum Chaudhry, Peter Lewis, Rishika Bose, Sahar Zandi Nia, Tamara
14 Dudley, and Cherri Zhang. Without their effort and support, this study would not have been
15 possible.

16

## 17 Data sharing

18 Study materials including the study protocol, consent form, data collection tools, de-identified
19 participant data, data dictionary, and the analysis code are available on request to the
20 corresponding author (Matthew O. Wiens, [matthew.wiens@bcchr.ca](mailto:matthew.wiens@bcchr.ca)) or to the Institute for
21 Global Health at BC Children’s and Women’s Hospital (Jessica Trawin,
22 [jessica.trawin@cw.bc.ca](mailto:jessica.trawin@cw.bc.ca)) or through the published protocol
23 (<https://doi.org/10.5683/SP3/QRUMNQ>) and dataset (<https://doi.org/10.5683/SP3/REPMSY>).
1 Owing to the sensitive nature of clinical data, access to the de-identified data is granted on a
2 case-by-case basis and will require the signing of a data sharing agreement.

## References

1. Rudd KE, Johnson SC, Agesa KM, Shackelford KA, Tsoi D, Kievlan DR, et al. Global, regional, and national sepsis incidence and mortality, 1990–2017: analysis for the Global Burden of Disease Study. Lancet. 2020;395: 200–211. doi:10.1016/S0140- 6736(19)32989-7
2. Rudd KE, Kissoon N, Limmathurotsakul D, Bory S, Mutahunga B, Seymour CW, et al. The global burden of sepsis: barriers and potential solutions. Crit Care. 2018;22: 232. doi:10.1186/s13054-018-2157-z
3. Wiens MO, Kissoon N, Holsti L. Challenges in pediatric post-sepsis care in resource limited settings: a narrative review. Transl Pediatr. 2021;10: 2666–2677. doi:10.21037/tp- 20-390
4. Knappett M, Nguyen V, Chaudhry M, Trawin J, Kabakyenga J, Kumbakumba E, et al. Pediatric post-discharge mortality in resource-poor countries: a systematic review and meta-analysis. EClinicalMedicine. 2024;67: 102380. doi:10.1016/j.eclinm.2023.102380
5. Childhood Acute Illness and Nutrition (CHAIN) Network. Childhood mortality during and after acute illness in Africa and south Asia: a prospective cohort study. Lancet Glob Heal. 2022;10: e673–e684. doi:10.1016/S2214-109X(22)00118-8
6. Wiens MO, Bone JN, Kumbakumba E, Businge S, Tagoola A, Sherine SO, et al. Mortality after hospital discharge among children younger than 5 years admitted with suspected sepsis in Uganda: a prospective, multisite, observational cohort study. Lancet Child Adolesc Heal. 2023;7: 555–566. doi:10.1016/S2352-4642(23)00052-4
7. Nemetchek B, English L, Kissoon N, Ansermino JM, Moschovis PP, Kabakyenga J, et al. Paediatric postdischarge mortality in developing countries: a systematic review. BMJ Open. 2018;8: e023445. doi:10.1136/bmjopen-2018-023445
8. Paul S, Tickell KD, Ojee E, Oduol C, Martin S, Singa B, et al. Knowledge, attitudes, and perceptions of Kenyan healthcare workers regarding pediatric discharge from hospital. Prazeres F, editor. PLoS One. 2021;16: e0249569. doi:10.1371/journal.pone.0249569
9. Krepiakevich A, Khowaja AR, Kabajaasi O, Nemetchek B, Ansermino JM, Kissoon N, et al. Out of pocket costs and time/productivity losses for pediatric sepsis in Uganda: a mixed-methods study. BMC Health Serv Res. 2021;21: 1252. doi:10.1186/s12913-021- 07272-9
10. Nemetchek B, Khowaja A, Kavuma A, Kabajaasi O, Olirus Owilli A, Ansermino JM, et al. Exploring healthcare providers’ perspectives of the paediatric discharge process in Uganda: a qualitative exploratory study. BMJ Open. 2019;9: e029526. doi:10.1136/bmjopen-2019-029526
11. English L, Kumbakumba E, Larson CP, Kabakyenga J, Singer J, Kissoon N, et al. Pediatric out-of-hospital deaths following hospital discharge: a mixed-methods study. Afr Health Sci. 2016;16: 883–891. doi:10.4314/ahs.v16i4.2
12. Ahmed SM, Brintz BJ, Talbert A, Ngari M, Pavlinac PB, Platts-Mills JA, et al. Derivation and external validation of a clinical prognostic model identifying children at risk of death following presentation for diarrheal care. PLOS Glob public Heal. 2023;3: e0001937. doi:10.1371/journal.pgph.0001937
13. Wiens MO, Kumbakumba E, Larson CP, Ansermino JM, Singer J, Kissoon N, et al. Postdischarge mortality in children with acute infectious diseases: derivation of

postdischarge mortality prediction models. BMJ Open. 2015;5: e009449. doi:10.1136/bmjopen-2015-009449

14. Collins GS, Reitsma JB, Altman DG, Moons KGM. Transparent Reporting of a multivariable prediction model for Individual Prognosis Or Diagnosis (TRIPOD): The TRIPOD Statement. Ann Intern Med. 2015;162: 55–63. doi:10.7326/M14-0697
15. Uganda Bureau of Statistics. Population & Censuses. 2022 [cited 5 Dec 2023]. Available: https:/[/www.ubos.org/explore](http://www.ubos.org/explore-statistics/20/)-[statistics/20/](http://www.ubos.org/explore-statistics/20/)
16. Goldstein B, Giroir B, Randolph A, International Consensus Conference on Pediatric Sepsis. International pediatric sepsis consensus conference: definitions for sepsis and organ dysfunction in pediatrics. Pediatr Crit Care Med. 2005;6: 2–8. doi:10.1097/01.PCC.0000149131.72248.E6
17. Wiens M, Kissoon N (Tex), Ansermino JM, Barigye C, Businge S, Kumbakumba E, et al. Smart Discharges to improve post-discharge health outcomes in children: A prospective before-after study with staggered implementation. In: Borealis, V1 [Internet]. 2023 [cited 5 Dec 2023]. Available: https://doi.org/10.5683/SP3/QRUMNQ
18. Wiens MO, Kissoon N, Kumbakumba E, Singer J, Moschovis PP, Ansermino JM, et al. Selecting candidate predictor variables for the modelling of post-discharge mortality from sepsis: a protocol development project. Afr Health Sci. 2016;16: 162–9. doi:10.4314/ahs.v16i1.22
19. Nemetchek BR, Liang L, Kissoon N, Ansermino MJ, Kabakyenga J, Lavoie PM, et al. Predictor variables for post-discharge mortality modelling in infants: a protocol development project. Afr Health Sci. 2018;18: 1214–1225. doi:10.4314/ahs.v18i4.43
20. Harris PA, Taylor R, Thielke R, Payne J, Gonzalez N, Conde JG. Research electronic data capture (REDCap)--a metadata-driven methodology and workflow process for providing translational research informatics support. J Biomed Inform. 2009;42: 377–81. doi:10.1016/j.jbi.2008.08.010
21. Harris PA, Taylor R, Minor BL, Elliott V, Fernandez M, O’Neal L, et al. The REDCap consortium: Building an international community of software platform partners. J Biomed Inform. 2019;95: 103208. doi:10.1016/j.jbi.2019.103208
22. Jeni LA, Cohn JF, De La Torre F. Facing Imbalanced Data Recommendations for the Use of Performance Metrics. Int Conf Affect Comput Intell Interact Work [proceedings] ACII. 2013;2013: 245–251. doi:10.1109/ACII.2013.47
23. Kuhn M. Building Predictive Models in R Using the caret Package. J Stat Softw. 2008;28. doi:10.18637/jss.v028.i05
24. R Core Team. R: A language and environment for statistical computing. In: R Founcation for Statistical Computing, Vienna, Austria [Internet]. 2021 [cited 5 Dec 2023]. Available: https:/[/www.r](http://www.r-project.org/)-[project.org/](http://www.r-project.org/)
25. Wiens MO, Kumbakumba E, Larson CP, Moschovis PP, Barigye C, Kabakyenga J, et al. Scheduled Follow-Up Referrals and Simple Prevention Kits Including Counseling to Improve Post-Discharge Outcomes Among Children in Uganda: A Proof-of-Concept Study. Glob Heal Sci Pract. 2016;4: 422–434. doi:10.9745/GHSP-D-16-00069
26. Akech S, Kwambai T, Wiens MO, Chandna A, Berkley JA, Snow RW. Tackling post- discharge mortality in children living in LMICs to reduce child deaths. Lancet Child Adolesc Heal. 2023;7: 149–151. doi:10.1016/S2352-4642(22)00375-3
27. Wiens MO, Kissoon N, Kabakyenga J. Smart Hospital Discharges to Address a Neglected Epidemic in Sepsis in Low- and Middle-Income Countries. JAMA Pediatr. 2018;172: 213–214. doi:10.1001/jamapediatrics.2017.4519
28. Janssen KJM, Vergouwe Y, Donders ART, Harrell FE, Chen Q, Grobbee DE, et al. Dealing with missing predictor values when applying clinical prediction models. Clin Chem. 2009;55: 994–1001. doi:10.1373/clinchem.2008.115345
29. Hoogland J, van Barreveld M, Debray TPA, Reitsma JB, Verstraelen TE, Dijkgraaf MGW, et al. Handling missing predictor values when validating and applying a prediction model to new patients. Stat Med. 2020;39: 3591–3607. doi:10.1002/sim.8682
30. Kabajaasi O, Trawin J, Derksen B, Komugisha C, Mwaka S, Waiswa P, et al. Transitions from hospital to home: A mixed methods study to evaluate pediatric discharges in Uganda. PLOS Glob public Heal. 2023;3: e0002173. doi:10.1371/journal.pgph.0002173
31. Wynants L, van Smeden M, McLernon DJ, Timmerman D, Steyerberg EW, Van Calster

B. Three myths about risk thresholds for prediction models. BMC Med. 2019;17: 192. doi:10.1186/s12916-019-1425-3

32. Ramspek CL, Jager KJ, Dekker FW, Zoccali C, van Diepen M. External validation of prognostic models: what, why, how, when and where? Clin Kidney J. 2020;14: 49–58. doi:10.1093/ckj/sfaa188
33. Otokiti AU, Ozoude MM, Williams KS, Sadiq-onilenla RA, Ojo SA, Wasarme LB, et al. The Need to Prioritize Model-Updating Processes in Clinical Artificial Intelligence (AI) Models: Protocol for a Scoping Review. JMIR Res Protoc. 2023;12: e37685. doi:10.2196/37685
34. Davis SE, Greevy RA, Lasko TA, Walsh CG, Matheny ME. Detection of calibration drift in clinical prediction models to inform model updating. J Biomed Inform. 2020;112: 103611. doi:10.1016/j.jbi.2020.103611
35. Mitchell M, Kan L. Digital Technology and the Future of Health Systems. Heal Syst Reform. 2019;5: 113–120. doi:10.1080/23288604.2019.1583040
36. Sharma V, Ali I, van der Veer S, Martin G, Ainsworth J, Augustine T. Adoption of clinical risk prediction tools is limited by a lack of integration with electronic health records. BMJ Heal Care Informatics. 2021;28: e100253. doi:10.1136/bmjhci-2020-100253
37. University of Oslo. The world’s largest health information management system — developed through global collaboration led by UiO. In: dhis2 [Internet]. [cited 5 Dec 2023]. Available: https://dhis2.org/
38. Endalamaw A, Erku D, Khatri RB, Nigatu F, Wolka E, Zewdie A, et al. Successes, weaknesses, and recommendations to strengthen primary health care: a scoping review. Arch Public Health. 2023;81: 100. doi:10.1186/s13690-023-01116-0
39. Manyazewal T, Woldeamanuel Y, Blumberg HM, Fekadu A, Marconi VC. The potential use of digital health technologies in the African context: a systematic review of evidence from Ethiopia. NPJ Digit Med. 2021;4: 125. doi:10.1038/s41746-021-00487-4
40. ClinicalTrials.gov. Smart Discharges to Improve Post-discharge Health Outcomes in Children. In: NCT05730452 [Internet]. 2023 [cited 5 Dec 2023]. Available: https://clinicaltrials.gov/ct2/show/NCT05730452
41. Madrid L, Casellas A, Sacoor C, Quintó L, Sitoe A, Varo R, et al. Postdischarge Mortality Prediction in Sub-Saharan Africa. Pediatrics. 2019;143: e20180606.

doi:10.1542/peds.2018-0606

42. Ngari MM, Fegan G, Mwangome MK, Ngama MJ, Mturi N, Scott JAG, et al. Mortality after Inpatient Treatment for Severe Pneumonia in Children: a Cohort Study. Paediatr Perinat Epidemiol. 2017;31: 233–242. doi:10.1111/ppe.12348

## Figure Legends

#### Figure 1. Variable selection for model development

**Figure 2. Study enrolment flow diagram**

**Figure 3. Performance of the final clinical model for 0-6 months (M6PD-C0-6) on the full dataset**. The points on the receiver operating characteristic (ROC), precision recall (PR), and gain curve plots indicate co-ordinates for the probability threshold at sensitivity=80%, with positive predictive value (PPV) and negative predictive value (NPV) also reported at this threshold.

**Figure 4. Performance of the final clinical model for 6-60 months (M6PD-C6-60) on the full dataset.** The points on the receiver operating characteristic (ROC), precision recall (PR), and gain curve plots indicate co-ordinates for the probability threshold at sensitivity=80%, with positive predictive value (PPV) and negative predictive value (NPV) also reported at this threshold.

**Online supplementary material.** S1) Details of study cohorts and variables used for the full and intermediary models; S2) Statistical Methods and Analyses; S3) Intermediary Clinical Variable Models – Variable Importance; S4) Intermediary Clinical and Social Variable Models – Variable Importance; S5) Intermediary Any Variable Models – Performance Metrics, Coefficients and Variable Importance; S6) Final Clinical Variable Models, M6PD-C0-6 and M6PD-C6-60 – Performance Metrics and Coefficients; S7) Final Clinical and Social Variable Models, M6PD- CS0-6 and M6PD-CS6-60 – Performance Metrics and Coefficients; S8) Final Any Variable Models, M6PD-A0-6 and M6PD-A6-60 – Performance Metrics and Coefficients; S9) Literature review.

Figure 1	[Click here to access/download;Figure;Fig1.eps](https://www.editorialmanager.com/pgph/download.aspx?id=309558&guid=46710177-9aa6-49c8-97e2-14e8d7634f19&scheme=1)[<https://www.editorialmanager.com/pgph/download.aspx?id=309558&guid=46710177-9aa6-49c8-97e2-14e8d7634f19&scheme=1>](https://www.editorialmanager.com/pgph/download.aspx?id=309558&guid=46710177-9aa6-49c8-97e2-14e8d7634f19&scheme=1)

Figure 2	[Click here to access/download;Figure;Fig2.eps](https://www.editorialmanager.com/pgph/download.aspx?id=309577&guid=152a59a3-43b2-4efc-bd63-b9679a955db2&scheme=1)[<https://www.editorialmanager.com/pgph/download.aspx?id=309577&guid=152a59a3-43b2-4efc-bd63-b9679a955db2&scheme=1>](https://www.editorialmanager.com/pgph/download.aspx?id=309577&guid=152a59a3-43b2-4efc-bd63-b9679a955db2&scheme=1)

Figure 3	[Click here to access/download;Figure;Fig3.eps](https://www.editorialmanager.com/pgph/download.aspx?id=309562&guid=201c12ff-204f-4d4e-b4ab-27f26a36f774&scheme=1)

1.0

# 0−6M ROC Plot

1.0

# 0−6M PR Plot

0.8	0.8

AUC (95% CI): 0.77 (0.74, 0.8)

PPV: 0.14

NPV: 0.97

0.058 (0.58, 0.802)

PRAUC: 0.23

0.058 (0.802, 0.137)

Precision (PPV)

0.6	0.6

Sensitivity

0.4	0.4

0.2	0.2

0.0

1.0

0	0.2	0.4	0.6	0.8	1

1 − Specificity

# 0−6M Probability Thresholds

0.0

100

0.0	0.2	0.4	0.6	0.8	1.0

Recall (Sensitivity)

# 0−6M Gain Curve

0.8	80

Sensitivity Specificity

Sensitivity/Specificity

% Mortality Identified

0.6	60

0.4	40

0.2	20

0.0

0.00	0.05	0.10	0.15	0.20	0.25	0.30

### Probability Threshold

0

0	20	40	60	80	100

% Cohort Sampled

# 0−6M Calibration Plot 0−6M Predicted Probabilities

4

Figure 4	[Click here to access/download;Figure;Fig4.eps](https://www.editorialmanager.com/pgph/download.aspx?id=309563&guid=2cde2b97-9240-49b3-ba02-c723e511870d&scheme=1)

1.0

# 6−60m ROC Plot

1.0

# 6−60m PR Plot

0.8	0.8

AUC (95% CI): 0.75 (0.72, 0.79)

PPV: 0.08

NPV: 0.98

0.036 (0.526, 0.798)

PRAUC: 0.17

0.036 (0.798, 0.079)

Precision (PPV)

0.6	0.6

Sensitivity

0.4	0.4

0.2	0.2

0.0

1.0

0	0.2	0.4	0.6	0.8	1

1 − Specificity

# 6−60m Probability Thresholds

0.0

100

0.0	0.2	0.4	0.6	0.8	1.0

Recall (Sensitivity)

# 6−60m Gain Curve

0.8	80

Sensitivity Specificity

Sensitivity/Specificity

% Mortality Identified

0.6	60

0.4	40

0.2	20

0.0

0.00	0.05	0.10	0.15	0.20	0.25	0.30

### Probability Threshold

0

0	20	40	60	80	100

% Cohort Sampled

# 6−60m Calibration Plot 6−60m Predicted Probabilities

4

Online supplementary material

[Click here to access/download](https://www.editorialmanager.com/pgph/download.aspx?id=309564&guid=dce4b866-bdb2-4adf-9d0a-e3b82d41e14c&scheme=1)

[**Supporting Information** PDModel_Supplementary_02Mar2024.pdf](https://www.editorialmanager.com/pgph/download.aspx?id=309564&guid=dce4b866-bdb2-4adf-9d0a-e3b82d41e14c&scheme=1)

Revised Manuscript with Track Changes

1 Prediction models for post-discharge mortality among under-five
2 children with suspected sepsis in Uganda: A multicohort analysis

3

4 *Authors:* **Matthew O Wiens**^1,2,3,16^, PhD; **Vuong Nguyen**^1^, PhD; **Jeffrey N Bone**^3^, MSc; **Elias**
5 **Kumbakumba**^4^, MBChB; **Stephen Businge**^5^, MBChB; **Abner Tagoola**^6^, MBChB; **Sheila**
6 **Oyella Sherine**^7^, MBChB; **Emmanuel Byaruhanga**^8^, MBChB; **Edward Ssemwanga**^9^,
7 MBChB; **Celestine Barigye**^10^, MBChB; **Jesca Nsungwa**^11^, MBChB; **Charles Olaro**^11^, MBChB;
8 **J Mark Ansermino**^1,2,3^, MBBCh; **Niranjan Kissoon**^3,12^, MD; **Joel Singer**^13^, PhD; **Charles P**
9 **Larson**^14^, MDCM; **Pascal M Lavoie**^3,12^, MDCM; **Dustin Dunsmuir**^1,3^, MSc; **Peter P**
10 **Moschovis**^15^, MD; **Stefanie Novakowski**^1,2^, PhD; **Clare Komugisha**^16^, BSc; **Mellon**
11 **Tayebwa**^16^, MA; **Douglas Mwesigwa**^16^, BA; **Martina Knappett**^1^, BSc; **Nicholas West**^3^, MSc;
12 **Nathan Kenya Mugisha**^16^, MBChB; **Jerome Kabakyenga**^17,18^, PhD 13
14 *Affiliations:* **[1]** Institute for Global Health at BC Children’s and Women’s Hospital, 305-4088 Cambie
15 Street, Vancouver, BC V5Z 2X8, Canada; **[2]** Dept of Anesthesiology, Pharmacology & Therapeutics,
16 University of British Columbia, 217-2176 Health Sciences Mall, Vancouver, BC V6T 1Z3, Canada; **[3]**
17 BC Children’s Hospital Research Institute, 938 West 28th Ave, Vancouver, BC V5Z 4H4, Canada; **[4]**
18 Dept of Paediatrics and Child Health, Mbarara University of Science and Technology, Mbarara, Uganda;
19 **[5]** Holy Innocents Children's Hospital, PO Box 1685, Plot 6, Kaberebere-Insingiro Rd., Mbarara,
20 Uganda; **[6]** Jinja Regional Referral Hospital, P.O Box 43, Rotary Road, Jinja City, Uganda; **[7]** Masaka
21 Regional Referral Hospital, Alex Ssebowa, Masaka, Uganda; **[8]** Kawempe National Referral Hospital,
22 Kampala, Uganda; **[9]** Villa Maria Hospital, Masaka, Uganda; **[10]** Mbarara Regional Referral Hospital,
23 Mbarara, Uganda; **[11]** Ministry of Health for the Republic of Uganda, Kampala, Uganda; **[12]** Dept of
24 Pediatrics, University of British Columbia, Rm 2D19, 4480 Oak Street, BC Children’s Hospital,
25 Vancouver, BC V6H 3V4, Canada; **[13]** School of Population and Public Health, University of British
26 Columbia, 2206 East Mall, Vancouver, BC V6T 1Z3, Canada; **[14]** School of Population and Global
27 Health, McGill University, Suite 1200, 2001 McGill College Avenue, Montréal, Québec H3A 1G1,
28 Canada; **[15]** Division of Global Health, Massachusetts General Hospital, 125 Nashua Street, Suite 722,
29 Boston, MA 02114, Massachusetts, USA; **[16]** Walimu, Plot 5-7, Coral Crescent, Kololo, P .O. Box 9924,
30 Kampala, Uganda; **[17]** Maternal Newborn & Child Health Institute, Mbarara University of Science and
31 Technology, Mbarara, Uganda; **[18]** Dept of Community Health, Faculty of Medicine, Mbarara
32 University of Science and Technology, Mbarara, Uganda.

33

34	*Short title:* Predicting post-discharge mortality in young children with sepsis 35

36 *Corresponding authors:* **Matthew O. Wiens.** Institute for Global Health at BC Children’s and
37 Women’s Hospital~~Centre for International Child Health~~, 305 - 4088 Cambie Street, Vancouver,
38 BC, V5Z 2X8, Canada. tel: +1-604-829-2562. email: [matthew.wiens@bcchr.ca](mailto:matthew.wiens@bcchr.ca); **Jerome**
39 **Kabakyenga.** Mbarara University of Science and Technology, Mbarara, Uganda. Tel: + 256-77-
40 259-0409. email: [jkabakyenga@must.ac.ug](mailto:jkabakyenga@must.ac.ug)

## 1 Abstract

2 In many low-income countries, over five percent of hospitalized children die following hospital
3 discharge. IThe lack of available tools to identify~~ing~~ those at risk of post-discharge mortality has
4 limited the ability to make progress towards improving~~e~~ outcomes. We aimed to develop
5 algorithms designed to predict post-discharge mortality among children admitted with suspected
6 sepsis. Four prospective cohort studies of children in two age groups (0-6 and 6-60 months) were
7 conducted between 2012-2021 in six Ugandan hospitals. Prediction models were derived for six-
8 months post-discharge mortality, based on candidate predictors collected at admission, each with
9 a maximum of eight variables, and internally validated using 10-fold cross-validation. 8,810
10 children were enrolled: 470 (5.3%) died in hospital; 257 (7.7%) and 233 (4.8%) post-discharge
11 deaths occurred in the 0-6-month and 6-60-month age groups, respectively. The primary models
12 had an area under the receiver operating characteristic curve (AUROC) of 0.77 (95%CI 0.74-
13 0.80) for 0-6-month-olds and 0.75 (95%CI 0.72-0.79) for 6-60-month-olds; mean AUROCs
14 among the 10 cross-validation folds were 0.75 and 0.73, respectively. Calibration across risk
15 strata was good: Brier scores were 0.07 and 0.04, respectively. The most important variables
16 included anthropometry and oxygen saturation. Additional variables included: illness duration,
17 jaundice-age interaction, and a bulging fontanelle among 0-6-month-olds; and prior admissions,
18 coma score, temperature, age-respiratory rate interaction, and HIV status among 6-60-month-
19 olds. Simple prediction models at admission with suspected sepsis can identify children at risk of
20 post-discharge mortality. Further external validation is recommended for different contexts.
21 Models can be digitally integrated into existing processes to improve peri-discharge care as
22 children transition from the hospital to the community.

23

## 1 Introduction

2 Morbidity and mortality secondary to sepsis disproportionately affect children in low- and
3 middle-income countries, where >85% of global cases and deaths occur [1]. Lower income
4 regions are plagued by poorly resilient health systems, widespread socio-economic deprivation,
5 and unique vulnerabilities, including malnutrition. Reducing the overall sepsis burden requires a
6 multi-pronged strategy that addresses three periods along the care continuum – pre-facility,
7 facility and post-facility [2]. Of these, post-facility issues have been largely neglected in
8 research, policy, and practice [3].
9 Robust epidemiological data for pediatric post-discharge mortality in the context of sepsis and
10 severe infection have been limited [4]. Growing evidence points to a significant burden of post-
11 discharge mortality, which accounts for as many deaths as the acute hospital phase of illness
12 [5,6]. While comorbid conditions such as malnutrition and anemia have been linked to risk, other
13 factors such as illness severity (at admission and discharge), prior hospitalizations, and
14 underlying social vulnerability, are also independently associated with poor post-discharge
15 outcomes [7]. However, we lack simple data-driven methods to identify those at highest risk of
16 mortality.
17 Current epidemiological evidence has demonstrated critical gaps in care following discharge [8].
18 Most post-discharge deaths occur at home, rather than during a subsequent readmission,
19 indicating poor health utilization among the most vulnerable. Effective healthcare utilization is
20 often hampered by poverty, community and family social dynamics, and poorly linked and
21 unresponsive health facilities [9–11]. Providing quality care during and after discharge is a
22 significant challenge in many facilities, in part due to severely strained human and material
23 resources.
1 Effective solutions to improving the transition of care from hospital to home within poorly
2 resourced health systems must be child-centred and focused on identifying the most vulnerable
3 children [12]. In this study, we aim to update the development and validation of clinical
4 prediction models that identify children, admitted with suspected sepsis, who are at risk of post-
5 discharge mortality [13].

6

## 1 Materials and Methods

2 Study design and approvals
3 Four independently funded, prospective observational cohort studies were conducted with a
4 primary objective of generating model-building data: two among children under six months and
5 two among children 6-60 months of age. These studies were approved by the Mbarara University
6 of Science and Technology Research Ethics Committee (No. 05/11-11, 10-Nov-2011; and No.
7 15/10-16, 27-Jan-2017) and the University of British Columbia – Children’s and Women’s
8 Health Centre of BC Research Ethics Board (H10-01927, 01-Dec-2011; and H16-02679, 09-
9 May-2017). Written informed consent was obtained from the parent or legal guardian of all study
10 participants. This manuscript adheres to the Transparent Reporting of a multivariable prediction
11 model for Individual Prognosis Or Diagnosis (TRIPOD) statement [14].
12 Study setting and population
13 Subjects were enrolled from six hospitals in Uganda (**Supplementary-S1**). These facilities serve
14 catchments of 30 districts with a population of approximately 8.2 million individuals, including
15 approximately 1.4 million children under five years [15], in a mix of urban and rural areas,
16 reflecting a representative sampling of the Ugandan pediatric population.
17 All study cohorts had identical eligibility criteria. Any child admitted with suspected sepsis was
18 eligible. Suspected sepsis was defined as children admitted with a proven or suspected infection
19 (as determined by the treating medical team). We previously demonstrated that 90% of children
20 enrolled using these criteria meet the international pediatric sepsis consensus conference
21 (IPSCC) definition [16]. The IPSCC defines sepsis as the presence of the systemic inflammatory
22 response syndrome alongside a suspected or proven infection.
1 The first cohort (enrolment 13-Mar-2012 to 13-Jan-2014) was used previously to report a
2 predictive model for post-discharge mortality in 6-60-month-olds [13]. The second and third
3 cohorts were the primary enrolment for the present analysis, and were defined by age range: 0-6-
4 month-olds (enrolment 11-Jan-2018 to 30-Mar-2020) and 6-60-month-olds (enrolment 13-Jul-
5 2017 to 02-Jul-2019); these data have been previously reported [6]. The fourth cohort enrolled
6 only 0-6-month-olds (enrolment 31-Mar-2020 to 05-Aug-2021) in order to understand how the
7 early COVID-19 period impacted post-discharge outcomes. Protocols and procedures were
8 largely overlapping, and the same research staff were involved in data collection during all four
9 enrolment periods [17].
10 Data collection
11 Data collection tools are available through the Smart Discharges Dataverse [17]. Data collection
12 procedures were previously described (also **Supplementary-S1**) [6,13]. Briefly, trained study
13 nurses collected clinical, social, and demographic data from consented participants at hospital
14 admission; largely overlapping between the two age groups, some variables were specific to 0-6-
15 month-olds. These variables were our candidate predictors and were selected based on clinical
16 and contextual knowledge of possible factors relating to post-discharge mortality, using a
17 modified Delphi process to identify promising variables in each age group [18,19].
18 Study nurses recorded discharge diagnosis and status (died, discharged, discharged against
19 medical advice, referred). A field officer contacted enrolled children by phone two and four
20 months after discharge, with an in-person visit at six months to determine mortality status and, if
21 applicable, date of death. All data were collected using encrypted study tablets and uploaded to a
22 Research Electronic Data Capture (REDCap) database hosted at the BC Children’s Hospital
23 Research Institute (Vancouver, Canada) [20,21].
1 Model development
2 *Outcome definition and ascertainment*
3 The primary outcome of the prediction model was post-discharge mortality within six months of
4 discharge, analyzed as a binary outcome. While data were available to build a time-to-event
5 prediction model, time of death was considered irrelevant for modelling mortality. Complete six-
6 month follow-up data for vital status was available for 98% of our cohort.
7 Prediction model performance was evaluated primarily using area under the receiver operating
8 characteristic curve (AUROC). We also reported the precision-recall curve and area under the
9 precision-recall curve (PR-AUC), which are more appropriate for imbalanced datasets [22].
10 *Variable selection*
11 Recognising the challenges of implementing large prediction models in resource-constrained
12 settings, we determined *a priori* to develop three models for each age group and restricted each
13 model to eight variables drawing from a different pool of available predictors: one model
14 focused solely on commonly-available *clinical* variables; one model focused on commonly-
15 available *clinical and social* variables; and one model used *any* candidate predictor variable
16 (**Figure 1**). This approach aimed to reduce the impact of missingness in an implementation
17 scenario. A feature of our modelling approach (elastic net regression) was that final model size
18 could not be pre-specified, often resulting in large models. Therefore, we conducted two rounds
19 of variable selection.

20

**Fig 1. Variable selection for model development**

21

22 To prioritize parsimony, the first variable selection round reduced the list of possible predictors
23 to two subsets: one including only the most relevant *clinical* variables; and a second including
1 only the most relevant *clinical and social* variables. Variables included in these subsets were
2 determined *a priori*, based on clinical significance and ease of measurement in low-resource
3 settings*.* These subsets were used to derive ***intermediary models*** that were either clinically-
4 focused or clinically- and socially-focused; the full candidate predictor list for each age group
5 was also used to derive ***intermediary models*** that used any available variable (**Supplementary-**

#### 6 Tables S1.2, S1.3).

7 The second variable selection round involved ranking the importance of variables from each
8 intermediary model, which was calculated as the weighted sums of the absolute regression
9 coefficients [23]. The top eight unique variables (e.g., temperature and its quadratic term were
10 considered a single unique variable) were selected based on average ranking from 10-fold cross-
11 validation of the intermediary models. If an interaction term was ranked in the top eight
12 variables, both interaction terms were included. This second variable selection round produced a
13 family of ***final models*** to predict mortality within six months post-discharge (M6PD) that used
14 only the eight top-ranked variables in each age group: models using only ***clinical*** variables,
15 denoted by M6PD-C0-6 for 0-6-month-olds and M6PD-C6-60 for 6-60-month-olds; models using
16 ***clinical and social*** variables, denoted by M6PD-CS0-6 and M6PD-CS6-60; and models using ***any***
17 of available predictor variable, denoted by M6PD-A0-6 and M6PD-A6-60 (**Figure 1**).
18 Statistical analysis
19 The primary study sample size was determined to accomplish three aims: to explore the
20 epidemiology of post-discharge mortality, as previously reported;[6] to develop prediction
21 models; and as a control period for a later interventional phase. The estimated sample size was
22 determined as 2,117 and 1,551 for the 0-6-month and 6-60-month cohorts, respectively
1 (**Supplementary-S2**). All analyses were conducted using R version 4.2.2 (R Foundation for
2 Statistical Computing, Vienna, Austria) [24], reported in detail in **Supplementary-S2**.

3

## 1 Results

2 Study population
3 During the four enrolment periods, a total of 22,166 consecutively admitted children were
4 screened and 8,810 enrolled (**Figure 2**). Among 0-6-month-olds (n=3,665), 3,424 (93.4%)
5 survived to discharge. Complete 6-month outcomes were available for 3,349 (97.8%) of these
6 children, forming the full dataset for model development in this age group. Among 6-60-month-
7 olds (n=5,145), 4,916 (95.5%) survived to discharge. Complete 6-month outcomes were
8 available for 4,830 (98.2%) of these children, forming the full dataset for model development in
9 this age group.

10

**Fig 2. Study enrolment flow diagram**

11

12 Mortality within 6 months of discharge occurred in 257 (7.7%) 0-6-month-olds, with median
13 (interquartile range [IQR]) time to death of 31 (9-80) days, and in 233 (4.8%) 6-60-month-olds,
14 with time to death of 36 (11-105) days (**Supplementary**-**Figure S1.1**). Missing data were
15 minimal (**Table 1**).
16 These cohorts’ clinical and demographic details have been previously described (**Table 1**).[6,13]
17 The mean ±standard deviation [SD] age was 2.1 ±1.8 months with 1,884 (56.3%) male in the 0-
18 6-month group, and 21.7 ±13.7 months with 2,670 (55.3%) male in the 6-60-month group. Poor
19 growth/malnutrition was common, with 463 (13.8%) 0-6-month-olds and 668 (13.8%) 6-60-
20 month-olds classified as severely underweight (weight-for-age z-score <-3) and similar weight-
21 for-age z-score distributions in both age groups. Discharge diagnoses recorded by the clinical
22 team could be overlapping in the case of multiple diagnoses (**Supplementary-Table S1.4**). Most
23 predictor variables considered were associated with post-discharge mortality (**Table 1**).

1	**Table 1.** Demographics and univariable odds ratios for the risk of post-discharge infant mortality.

|  | **0-6 month (n = 3349)** | | | | **6-60 month (n = 4830)** | | | |
| --- | --- | --- | --- | --- | --- | --- | --- | --- |
| **Variable** | **n (%)/Mean (SD)** | **n Missing (%)** | **OR (95%CI)** | **P-value** | **n (%)/Mean (SD)** | **n Missing (%)** | **OR (95%CI)** | **P-value** |
| **A) Demographics** | | | | | | | | |
| **Sex, male** | 1884 (56.3%) | 0 (0%) | 1.18 (0.91, 1.53) | 0.218 | 2670 (55.3%) | 0 (0%) | 0.9 (0.69, 1.17) | 0.433 |
| **Age, months** | 2.1 (1.8) | 0 (0%) | 1.05 (0.98, 1.12) | 0.188 | 21.7 (13.7) | 1 (0.02%) | 1 (0.99, 1.01) | 0.471 |
| **B) Admission Anthropometry** | | | | | | | | |
| **BMI Z-scores** | -1 (2.2) | 5 (0.15%) | 0.78 (0.74, 0.82) | **<0.001** | -1 (9.8) | 32 (0.66%) | 0.86 (0.81, 0.91) | **<0.001** |
| ***< -3*** | 565 (16.9%) |  | 4.31 (3.24, 5.75) | **<0.001** | 775 (16%) |  | 2.31 (1.68, 3.16) | **<0.001** |
| ***-3 to -2*** | 399 (11.9%) |  | 2.36 (1.61, 3.40) | **<0.001** | 684 (14.2%) |  | 1.84 (1.28, 2.60) | **0.001** |
| ***> -2*** | 2380 (71.1%) |  | ref. | **<0.001** | 3339 (69.1%) |  | ref. | **<0.001** |
| **MUAC, mm a** | 113.7 (17.7) | 3 (0.09%) | 0.96 (0.96, 0.97) | **<0.001** | 139.2 (16.1) | 18 (0.37%) | 0.96 (0.95, 0.97) | **<0.001** |
| ***<110 / <115*** | 1304 (38.9%) |  | 3.81 (2.7, 5.51) | **<0.001** | 321 (6.6%) |  | 6.66 (4.76, 9.25) | **<0.001** |
| ***110-120 / 115-125*** | 942 (28.1%) |  | 1.58 (1.04, 2.42) | **0.033** | 514 (10.6%) |  | 2.77 (1.92, 3.92) | **<0.001** |
| ***>120 / >125*** | 1100 (32.8%) |  | ref. | **<0.001** | 3977 (82.3%) |  | ref. | **<0.001** |
| **Weight for age Z-scores** | -1.1 (2) | 2 (0.06%) | 0.71 (0.67, 0.75) | **<0.001** | -1.3 (1.7) | 12 (0.25%) | 0.71 (0.66, 0.76) | **<0.001** |
| ***< -3*** | 463 (13.8%) |  | 6.15 (4.58, 8.26) | **<0.001** | 668 (13.8%) |  | 4.58 (3.40, 6.17) | **<0.001** |
| ***-3 to -2*** | 356 (10.6%) |  | 3.61 (2.51, 5.14) | **<0.001** | 723 (15%) |  | 1.77 (1.20, 2.55) | **0.003** |
| ***> -2*** | 2528 (75.5%) |  | ref. | **<0.001** | 3427 (71%) |  | ref. | **<0.001** |
| **Weight for length Z-scores** | -1 (2.6) | 5 (0.15%) | 0.87 (0.84, 0.91) | **<0.001** | -1.2 (2) | 30 (0.62%) | 0.83 (0.78, 0.89) | **<0.001** |
| ***< -3*** | 627 (18.7%) |  | 2.52 (1.88, 3.35) | **<0.001** | 725 (15%) |  | 2.52 (1.83, 3.45) | **<0.001** |
| ***-3 to -2*** | 365 (10.9%) |  | 1.73 (1.16, 2.52) | **0.006** | 718 (14.9%) |  | 1.86 (1.30, 2.61) | **<0.001** |
| ***> -2*** | 2352 (70.2%) |  | ref. | **<0.001** | 3357 (69.5%) |  | ref. | **<0.001** |
| **C) Admission Clinical Assessment** | | | | | | | | |
| **How long ago since last admission** | 20 (0.6%) | | | | 20 (0.41%) | | | |
| ***Never*** | 2848 (85%) |  | ref. | **<0.001** | 2647 (54.8%) |  | ref. | **<0.001** |
| ***< 7days*** | 122 (3.6%) |  | 2.04 (1.12, 3.47) | **0.013** | 191 (4%) |  | 2.37 (1.34, 3.94) | **0.002** |
| ***7 days to <1 month*** | 180 (5.4%) |  | 2.68 (1.71, 4.06) | **<0.001** | 400 (8.3%) |  | 2.39 (1.60, 3.52) | **<0.001** |
| ***1 month to <1 year*** | 179 (5.3%) |  | 2.47 (1.56, 3.79) | **<0.001** | 1175 (24.3%) |  | 1.42 (1.03, 1.94) | **0.031** |
| ***≥1 year*** | 0 (0%) | | | | 397 (8.2%) |  | 0.50 (0.22, 0.97) | 0.06 |
| **SpO2** | 93.8 (6.8) | 9 (0.27%) | 0.96 (0.95, 0.98) | **<0.001** | 94.2 (6.5) | 22 (0.46%) | 0.95 (0.94, 0.97) | **<0.001** |
| ***< 90%*** | 598 (17.9%) |  | 1.78 (1.31, 2.41) | **<0.001** | 774 (16%) |  | 2.07 (1.49, 2.84) | **<0.001** |
| ***90% to 95%*** | 891 (26.6%) |  | 0.87 (0.62, 1.20) | 0.406 | 1236 (25.6%) |  | 1.15 (0.82, 1.58) | 0.404 |
| ***> 95%*** | 1851 (55.3%) |  | ref. | **<0.001** | 2798 (57.9%) |  | ref. | **<0.001** |
| **Heart rate, beats per minute** | 149.2 (23.6) | 3 (0.09%) | 1.00 (0.99, 1.00) | 0.276 | 144.8 (25.5) | 3 (0.06%) | 1.00 (0.99, 1.00) | 0.599 |

|  | **0-6 month (n = 3349)** | | | | **6-60 month (n = 4830)** | | | |
| --- | --- | --- | --- | --- | --- | --- | --- | --- |
| **Variable** | **n (%)/Mean (SD)** | **n Missing (%)** | **OR (95%CI)** | **P-value** | **n (%)/Mean (SD)** | **n Missing (%)** | **OR (95%CI)** | **P-value** |
| **Respiratory rate, breaths per minute** | 57.4 (17) | 5 (0.15%) | 1 (0.99, 1.01) | 0.875 | 48.1 (15.7) | 7 (0.14%) | 1.01 (1.00, 1.02) | **0.003** |
| **Systolic blood pressure, mmHg** | 85.1 (16.5) | 10 (0.3%) | 0.99 (0.99, 1.00) | 0.08 | 95.2 (13.4) | 8 (0.17%) | 0.99 (0.98, 1.00) | **0.028** |
| **Diastolic blood pressure, mmHg** | 46.3 (12.8) | 10 (0.3%) | 0.99 (0.98, 1.00) | 0.213 | 54.4 (11.6) | 8 (0.17%) | 0.99 (0.98, 1.00) | 0.079 |
| **Temperature, °C** | 37.4 (0.9) | 1 (0.03%) | 0.90 (0.78, 1.04) | 0.167 | 37.7 (1.2) | 3 (0.06%) | 0.81 (0.72, 0.91) | **<0.001** |
| ***< 36.5*** | 386 (11.5%) |  | 0.96 (0.62, 1.43) | 0.835 | 505 (10.5%) |  | 1.28 (0.84, 1.89) | 0.234 |
| ***36.5 to 37.5*** | 1699 (50.7%) |  | ref. | 0.581 | 1868 (38.7%) |  | ref. | **0.014** |
| ***37.6 to 39*** | 1072 (32%) |  | 1.01 (0.76, 1.34) | 0.923 | 1638 (33.9%) |  | 0.82 (0.6, 1.12) | 0.222 |
| ***> 39*** | 191 (5.7%) |  | 0.65 (0.32, 1.20) | 0.202 | 816 (16.9%) |  | 0.58 (0.37, 0.89) | **0.016** |
| **Abnormal BCS score** | 285 (8.5%) | 0 (0%) | 2.37 (1.64, 3.34) | **<0.001** | 408 (8.4%) | 0 (0%) | 1.93 (1.30, 2.78) | **0.001** |
| **Malaria test positive** | 324 (9.7%) | 1 (0.03%) | 0.56 (0.31, 0.92) | **0.032** | 1480 (30.6%) | 11 (0.23%) | 0.76 (0.56, 1.02) | 0.075 |
| **HIV+** | 119 (3.6%) | 2 (0.06%) | 1.37 (0.70, 2.42) | 0.317 | 144 (3%) | 22 (0.46%) | 3.81 (2.31, 6.00) | **<0.001** |
| **Haemoglobin, g/dL** | 13 (3.3) | 4 (0.12%) | 0.96 (0.92, 1.00) | **0.036** | 10.4 (3.2) | 608 (12.59%) b | 0.88 (0.85, 0.92) | **<0.001** |
| ***No anaemia*** | 2435 (72.7%) |  | ref. | **0.003** | 1983 (41.1%) |  | ref. | **<0.001** |
| ***Mild anaemia*** | 788 (23.5%) |  | 1.29 (0.96, 1.72) | 0.091 | 1535 (31.8%) |  | 1.59 (1.15, 2.21) | **0.006** |
| ***Severe anaemia*** | 122 (3.6%) |  | 2.47 (1.44, 4.04) | **0.001** | 704 (14.6%) |  | 2.67 (1.87, 3.82) | **<0.001** |
| **D) Maternal and Social Characteristics** | | | | | | | | |
| **Time it took to reach hospital** | 0 (0%) | | | | 1 (0.02%) | | | |
| ***<30 minutes*** | 806 (24.1%) |  | ref. | **<0.001** | 1015 (21%) |  | ref. | **<0.001** |
| ***30 minutes to <1 hour*** | 1224 (36.5%) |  | 1.15 (0.77, 1.75) | 0.498 | 1519 (31.4%) |  | 1.67 (1.04, 2.75) | **0.037** |
| ***≥1 hour*** | 1319 (39.4%) |  | 2.65 (1.86, 3.88) | **<0.001** | 2295 (47.5%) |  | 2.89 (1.90, 4.58) | **<0.001** |
| **Maternal age, years** | 26.3 (5.7) | 50 (1.49%) | 1.00 (0.98, 1.02) | 0.871 | 27.9 (6.4) | 167 (3.46%) | 1.00 (0.98, 1.02) | 0.899 |
| **Number of children** | 2.8 (1.8) | 1 (0.03%) | 1.01 (0.94, 1.08) | 0.87 | 3.2 (2.1) | 3 (0.06%) | 1.04 (0.98, 1.10) | 0.228 |
| **Had a child who died previously** | 577 (17.2%) | 1 (0.03%) | 1.21 (0.87, 1.65) | 0.249 | 1066 (22.1%) | 3 (0.06%) | 1.27 (0.93, 1.70) | 0.123 |
| **Maternal education** | 16 (0.48%) | | | | 49 (1.01%) | | | |
| ***No school*** | 105 (3.1%) |  | ref. | **0.001** | 334 (6.9%) |  | ref. | **<0.001** |
| ***≤P3*** | 207 (6.2%) |  | 1.16 (0.57, 2.48) | 0.684 | 345 (7.1%) |  | 1.30 (0.70, 2.43) | 0.411 |
| ***P4-P7*** | 1327 (39.6%) |  | 0.71 (0.39, 1.41) | 0.297 | 2088 (43.2%) |  | 0.98 (0.61, 1.65) | 0.922 |
| ***S1-S6*** | 1175 (35.1%) |  | 0.58 (0.32, 1.16) | 0.098 | 1517 (31.4%) |  | 0.61 (0.36, 1.07) | 0.073 |
| ***Post-Secondary*** | 519 (15.5%) |  | 0.36 (0.18, 0.77) | **0.006** | 497 (10.3%) |  | 0.41 (0.19, 0.85) | **0.018** |
| **Maternal HIV** | 1 (0.03%) | | | | 6 (0.12%) | | | |
| ***No*** | 3052 (91.1%) |  | ref. | 0.125 | 3915 (81.1%) |  | ref. | **0.012** |
| ***Yes*** | 246 (7.3%) |  | 1.48 (0.95, 2.24) | 0.071 | 432 (8.9%) |  | 1.63 (1.07, 2.40) | **0.016** |
| ***Unknown*** | 50 (1.5%) |  | 1.71 (0.65, 3.77) | 0.222 | 477 (9.9%) |  | 1.57 (1.05, 2.29) | **0.023** |

|  | **0-6 month (n = 3349)** | | | | **6-60 month (n = 4830)** | | | |
| --- | --- | --- | --- | --- | --- | --- | --- | --- |
| **Variable** | **n (%)/Mean (SD)** | **n Missing (%)** | **OR (95%CI)** | **P-value** | **n (%)/Mean (SD)** | **n Missing (%)** | **OR (95%CI)** | **P-value** |
| **Bed net use** | 1 (0.03%) | | | | 3 (0.06%) | | | |
| ***Never*** | 2809 (83.9%) |  | 0.85 (0.44, 1.57) | 0.611 | 3630 (75.2%) |  | 1.05 (0.64, 1.73) | 0.841 |
| ***Sometimes*** | 337 (10.1%) |  | ref. | 0.544 | 631 (13.1%) |  | ref. | 0.443 |
| ***Always*** | 202 (6%) |  | 0.80 (0.55, 1.20) | 0.262 | 566 (11.7%) |  | 0.85 (0.59, 1.26) | 0.389 |
| **Water source** | 0 (0%) | | | | 3 (0.06%) | | | |
| ***Bore hole*** | 655 (19.6%) |  | 1.72 (1.22, 2.4) | **0.002** | 1042 (21.6%) |  | 2.61 (1.84, 3.72) | **<0.001** |
| ***Fast running water*** | 21 (0.6%) |  | 0.84 (0.05, 4.08) | 0.862 | 515 (10.7%) |  | 1.76 (1.08, 2.80) | **0.019** |
| ***Municipal water*** | 1630 (48.7%) |  | ref. | **<0.001** | 1981 (41%) |  | ref. | **<0.001** |
| ***Open source*** | 541 (16.2%) |  | 2.12 (1.51, 2.98) | **<0.001** | 558 (11.6%) |  | 1.88 (1.19, 2.93) | **0.006** |
| ***Protected spring*** | 392 (11.7%) |  | 1.49 (0.97, 2.23) | 0.063 | 590 (12.2%) |  | 2.03 (1.30, 3.11) | **0.001** |
| ***Slow running water*** | 110 (3.3%) |  | 1.67 (0.80, 3.16) | 0.14 | 141 (2.9%) |  | 1.99 (0.86, 4.03) | 0.075 |
| **Boil/disinfect/filter water** | 2526 (75.4%) | 0 (0%) | 0.84 (0.64, 1.13) | 0.237 | 3402 (70.4%) | 2 (0.04%) | 0.51 (0.39, 0.67) | **<0.001** |
| **E) Discharge Characteristics** | | | | | | | | |
| **Length of stay, days** | 5.6 (4.4) | 0 (0%) |  |  | 5.1 (8.2) | 0 (0%) |  |  |
| **Discharge status** | 2 (0.06%) | | | | 0 (0%) | | | |
| ***Referred to higher level of care*** | 164 (4.9%) | | | | 101 (2.1%) | | | |
| ***Routine discharge*** | 2810 (83.9%) | | | | 4143 (85.8%) | | | |
| ***Unplanned discharge*** | 373 (11.1%) | | | | 586 (12.1%) | | | |
| **F) Variables collected only for 0-6-month** | | | | | | | | |
| **Abdominal distension** | 217 (6.5%) | 2 (0.06%) | 1.79 (1.15, 2.70) | **0.007** |  | | | |
| **Antenatal visits** | 4.9 (1) | 48 (1.43%) | 0.89 (0.78, 1.01) | 0.066 |  |  |  |  |
| **Dehydration, WHO categories** | 11 (0.33%) | | | |  |  |  |  |
| ***No dehydration*** | 2844 (84.9%) |  | ref. | **<0.001** |  |  |  |  |
| ***Some dehydration*** | 399 (11.9%) |  | 1.64 (1.15, 2.30) | **0.005** |  |  |  |  |
| ***Severe dehydration*** | 95 (2.8%) |  | 3.40 (1.96, 5.62) | **<0.001** |  |  |  |  |
| **Delivery method, caesarean** | 497 (14.8%) | 4 (0.12%) | 0.74 (0.49, 1.08) | 0.136 |  |  |  |  |
| **Duration of present illness** | 4 (0.12%) | | | |  |  |  |  |
| ***<48 hours*** | 957 (28.6%) |  | ref. | **<0.001** |  |  |  |  |
| ***48 hours to 7 days*** | 60 (1.8%) |  | 1.46 (1.05, 2.06) | **0.026** |  |  |  |  |
| ***8 days to 1 month*** | 1985 (59.3%) |  | 3.16 (2.09, 4.80) | **<0.001** |  |  |  |  |
| ***>1 month*** | 343 (10.2%) |  | 5.13 (2.52, 9.87) | **<0.001** |  |  |  |  |
| **Fontanelle** | 132 (3.9%) | 6 (0.18%) | 2.4 (1.44, 3.81) | **<0.001** |  |  |  |  |
| **Glucose, mmol/L** | 5.7 (2.5) | 2 (0.06%) | 1.03 (0.98, 1.08) | 0.188 |  |  |  |  |

|  | **0-6 month (n = 3349)** | **6-60 month (n = 4830)** |
| --- | --- | --- |
| **Variable** | **n (%)/Mean (SD) n Missing (%) OR (95%CI) P-value** | **n (%)/Mean (SD) n Missing (%) OR (95%CI) P-value** |
| **Not previously tested for HIV** | 2968 (88.6%) 0 (0%) 0.93 (0.64, 1.40) 0.719 |  |
| **Referral visit** | 1056 (31.5%) 1 (0.03%) 1.70 (1.31, 2.20) **<0.001** |  |
| **Neonatal jaundice** | 261 (7.8%) 34 (1.02%) 1.31 (0.83, 1.99) 0.219 |  |
| **Lactate level, mmol/L** | 2.5 (1.6) 9 (0.27%) 1.10 (1.03, 1.18) **0.003** |  |
| **Mother currently acutely ill** | 132 (3.9%) 13 (0.39%) 0.56 (0.22, 1.18) 0.171 |  |
| **Mother has chronic illness** | 251 (7.5%) 19 (0.57%) 1.29 (0.81, 1.97) 0.256 |  |
| **Child less than 30 days old** | 1353 (40.4%) 2 (0.06%) 0.68 (0.52, 0.89) **0.006** |  |
| **Pallor** | 307 (9.2%) 2 (0.06%) 2.15 (1.50, 3.03) **<0.001** |  |
| **Premature birth** | 210 (6.3%) 6 (0.18%) 2.05 (1.33, 3.06) **0.001** |  |
| **Prior care sought for current illness** | 1995 (59.6%) 0 (0%) 1.82 (1.38, 2.42) **<0.001** |  |
| **Sucking well when breastfeeding, or** | 1956 (58.4%) 8 (0.24%) 0.47 (0.36, 0.61) **<0.001** |  |
| **feeding well if not breastfed** |  |  |
| **Sucking well when breastfeeding, or** |  |  |
| **feeding well if not breastfed, prior to** | 2589 (77.3%) 392 (11.7%) 0.59 (0.42, 0.85) **0.004** |  |
| **illness** |  |  |
| **When did the baby cry after birth** | 97 (2.9%) |  |
| ***Immediately*** | 2805 (83.8%) ref. **0.041** |  |
| ***<5 minutes*** | 138 (4.1%) 1.83 (1.04, 3.02) **0.025** |  |
| ***5 to 10 minutes*** | 141 (4.2%) 1.32 (0.70, 2.30) 0.351 |  |
| ***11 to 30 minutes*** | 68 (2%) 1.26 (0.48, 2.72) 0.594 |  |
| ***>30 minutes*** | 100 (3%) 2.12 (1.14, 3.68) **0.011** |  |
| **Abnormal tone** | 285 (8.5%) 2 (0.06%) 3.11 (2.21, 4.31) **<0.001** |  |
| **Decreased urine production** | 677 (20.2%) 99 (2.96%) 1.91 (1.43, 2.52) **<0.001** |  |

1 For non-binary categorical variables, the p-value for the reference group (labelled ref.) indicates the global p-value. Odds ratios and p-values were not calculated
2 for discharge variables.
3 a MUAC thresholds given for 0-6-month / 6-60-month cohorts
4 b High prevalence of missing data for hemoglobin due to faulty capillary tubes during data collection
5 Abbreviations: BCS = Blantyre coma scale; BMI = body mass index; HIV+ = human immunodeficiency virus positive; MUAC = mid-upper arm circumference;
6 OR = odds ratio; SpO2 = oxygen saturation; WHO = World Health Organization

7

1 Prediction models
2 The intermediary variable models were large (coefficients, performance metrics, and variable
3 importance reported in **Supplementary S3-S5**). The models derived using all candidate
4 predictors (intermediary *any* variable model) included 41 unique variables in the 0-6-month
5 model and 19 unique variables in the 6-60-month model. Applied to the entire dataset for each
6 age group, the AUROC was 0.81 (95%CI 0.79 to 0.84) for the 0-6-month model and 0.79
7 (95%CI 0.77 to 0.82) for the 6-60-month model, with average AUROCs of 0.77 (range 0.69-
8 0.87) and 0.76 (range 0.71-0.81) across the 10 cross-validations, respectively; the PR-AUC was
9 0.27 for the 0-6-month model and 0.18 for the 6-60-month model, with average PR-AUCs of
10 0.22 (range 0.13-0.31) and 0.16 (range 0.11-0.21) across the 10 cross-validations, respectively.
11 Calibration was good at low predicted probabilities, with a Brier scores of 0.07 (range 0.06-0.07)
12 for the 0-6-month model and 0.04 (range 0.04-0.05) for the 6-60-month model. Calibration
13 decreased at higher predicted probabilities, although there were almost no individuals with
14 probabilities >40%. In both age groups, mid-upper arm circumference (MUAC) was identified as
15 the variable with the highest importance.
16 The final models are summarized in **Table 2**, and detailed in **Supplementary S6-S8**, including
17 all model terms, their coefficients, and plots outlining the relative importance of coefficients in
18 each model.

1 **Table 2.** Summary of performance and variables included in the set of final models with reduced number
2 of variables using the probability threshold that gave a sensitivity of 0.8.

| **A) 0-6-month models** | **M6PD-C0-6** | **M6PD-CS0-6 *** | **M6PD-A0-6 *** |
| --- | --- | --- | --- |
| **Average Cross-Validation Performance** |  |  |  |
| Specificity | 0.60 | 0.61 | 0.61 |
| AUROC | 0.75 | 0.76 | 0.76 |
| PPV | 0.15 | 0.16 | 0.16 |
| NPV | 0.97 | 0.97 | 0.97 |
| PRAUC | 0.23 | 0.23 | 0.23 |
| Brier Score | 0.07 | 0.07 | 0.07 |
| **Full Dataset Performance**  Specificity | 0.58 | 0.62 | 0.62 |
| AUROC | 0.77 | 0.77 | 0.77 |
| PPV | 0.14 | 0.15 | 0.15 |
| NPV | 0.97 | 0.97 | 0.97 |
| PRAUC | 0.23 | 0.22 | 0.22 |
| Brier Score | 0.07 | 0.07 | 0.07 |
| **Variables**  Age, months | ✓ ✓ ✓ | | |
| Duration of present illness, categorical | ✓ ✓ ✓ | | |
| MUAC, mm | ✓ ✓ ✓ | | |
| Neonatal jaundice, binary | ✓ ✓ ✓ | | |
| Sucking well when breastfeeding, binary | ✓ ✓ ✓ | | |
| SpO2, % | ✓ ✓ ✓ | | |
| Time to reach hospital, categorical | ✓ ✓ | | |
| Weight for age z-score | ✓ ✓ ✓ | | |
| Fontanelle, binary | ✓ | | |
| **B) 6-60-month models** | **M6PD-C6-60 M6PD-CS6-60 M6PD-A6-60** | | |
| **Average Cross-Validation Performance**  Specificity | 0.57 0.59 0.54 | | |
| AUROC | 0.73 0.74 0.75 | | |
| PPV | 0.09 0.09 0.08 | | |
| NPV | 0.98 0.98 0.98 | | |
| PRAUC | 0.16 0.15 0.15 | | |
| Brier Score | 0.04 0.04 0.04 | | |
| **Full Dataset Performance**  Specificity | 0.53 | 0.58 | 0.55 |
| AUROC | 0.75 | 0.76 | 0.77 |
| PPV | 0.08 | 0.09 | 0.08 |
| NPV | 0.98 | 0.98 | 0.98 |
| PRAUC | 0.17 | 0.16 | 0.17 |
| Brier Score | 0.04 | 0.04 | 0.04 |
| **Variables**  Age, months | ✓ ✓ ✓ | | |
| Haemoglobin, g/dl | ✓ | | |
| HIV, binary | ✓ ✓ | | |
| How long since last admission, categorical | ✓ ✓ ✓ | | |
| MUAC, mm | ✓ ✓ ✓ | | |
| SpO2, % | ✓ ✓ ✓ | | |

| Water source, categorical | ✓ ✓ |
| --- | --- |
| Weight for age z-score | ✓ ✓ ✓ |
| Abnormal BCS, binary | ✓ |
| Respiratory rate, bpm | ✓ |
| Temperature, °C | ✓ |
| Boil/disinfect/filter water | ✓ |

1 * Note, the 0-6-month final reduced (M6PD-A0-6) and final clinical and social model (M6PD-CS0-6) are identical
2 since the same variables were selected.
3 Abbreviations: AUROC = area under the receiver operating curve; BCS = Blantyre coma scale; bpm = breaths per
4 minute; HIV, human immunodeficiency virus; MUAC = mid-upper arm circumference; NPV = negative predictive
5 value; PPV = positive predictive value; PRAUC = area under the precision-recall curve; SpO2 = oxygen saturation

6

7 Final 0-6-month models
8 The M6PD-C0-6 model, using only simple *clinical* variables, included weight-for-age z-score
9 (mean rank [rm] =1.4, selection frequency [sf] =10), MUAC (rm=1.6, sf=10), feeding status
10 (rm=3.4, sf=10), SpO2 (rm=5.8, sf=9), duration of illness (rm=6.2, sf=9), age × jaundice (rm=7.8,
11 sf=7), and bulging fontanelle (rm=8.3, sf=8) (**Supplementary-Table S3.1)**. The AUROC was
12 0.77 (95%CI 0.74 to 0.80) and PR-AUC was 0.23 when applied to the entire 0-6-month dataset
13 (**Figure 3~~A~~**), while the average AUROC and PR-AUC across the internal 10 cross-validations
14 were 0.75 (range 0.63-0.85) and 0.23 (range 0.11-0.33), respectively (**Table 2A;**
15 **Supplementary-S6**). Setting the sensitivity to 80%, the corresponding probability threshold was
16 0.058; at this threshold, positive and negative predictive values were 14% and 97%, respectively.
17 Calibration at low predicted probabilities was good, with a Brier score of 0.07 (**Figure 3~~A~~,**
18 **Supplementary-Figure S6.3**). Calibration at probabilities beyond 30-40% was poor, but sample
19 sizes were very small in this range.

20

**Fig 3. Performance of the final clinical model~~s~~ for 0-6 months (M6PD-C0-6) on the full dataset.** The points on the receiver operating characteristic (ROC), precision recall (PR), and gain curve plots indicate co-ordinates for the probability threshold at sensitivity=80%, with positive predictive value (PPV) and negative predictive value (NPV) also reported at this threshold.

21

22

23

24 The M6PD-CS0-6 model, using *social and clinical* variables, was nearly identical in performance
25 to M6PD-C0-6; the variables were largely overlapping with only fontanelle status replaced by
1 travel time required to reach hospital (**Table 2A; Supplementary-S7**). M6PD-A0-6 that used *any*
2 available variable, was identical to M6PD-CS0-6 (**Table 2A; Supplementary-S8**).
3 Final 6-60-month models
4 The M6PD-C6-60 model, using only *clinical* predictors, included nine variables (the 8^th^ best-
5 performing variable included an interaction with a new variable; **Supplementary-Table S3.2**):
6 MUAC (rm=1, sf=10), SpO2 (rm=2.7, sf=10), weight-for-age z-score (rm=2.8, sf=10), time since
7 prior admission (rm=4.7, sf=10), abnormal coma score (rm=5.8, sf=9), temperature (rm=6.4, sf=9),
8 HIV status (rm=6.5, sf=9) and age × respiratory rate (rm=9.1, sf=2). The AUROC was 0.74
9 (95%CI 0.72 to 0.79) and PR-AUC was 0.17 when applied to the entire 6-60-month dataset
10 (**Figure 4~~3B~~**), with an average AUROC of 0.73 (range 0.67-0.77) and average PR-AUC of 0.16
11 (range 0.10-0.19) across the 10 cross-validations (**Table 2B; Supplementary-S6**). Setting
12 sensitivity to 80%, the corresponding probability threshold was 0.036; at this threshold, positive
13 and negative predictive values were 0.08 and 0.98, respectively. Calibration across risk strata
14 was good with a Brier score of 0.04 (**Figure 4~~3B~~, Supplementary-Figure S6.4**).

15

**Fig 4. Performance of the final clinical model for 6-60 months (M6PD-C6-60) on the full dataset.** The points on the receiver operating characteristic (ROC), precision recall (PR), and gain curve plots indicate co-ordinates for the probability threshold at sensitivity=80%, with positive predictive value (PPV) and negative predictive value (NPV) also reported at this threshold.

16

17

18

19 The M6PD-CS6-60 model, which used *clinical and social* variables, was almost identical to
20 M6PD-C6-60, with only home water source and water disinfection practices replacing coma score
21 (**Table 2B; Supplementary-S7**). M6PD-A6-60 was similar to M6PD-CS6-60, with water
22 disinfection practices replaced by hemoglobin; performance metrics were nearly identical (**Table**

#### 23 2B; Supplementary-S8).

24

## 1 Discussion

2 Using four large, objective-driven, prospective cohorts of under-5 children admitted with
3 suspected sepsis, we derived and internally-validated prediction models for post-discharge
4 mortality using only admission data. Their performance to predict mortality up to six months
5 post-discharge was good, suggesting potential utility to improve post-discharge outcomes by
6 linking individual risk to interventional intensity [25]. Data-driven, child-centred approaches to
7 post-discharge care have been strongly advocated for [5,26,27]. Our robust, cross-validated
8 models utilized data from multiple sites, captured over eight years, and should spur focus on
9 external validation outside Uganda.
10 Several recent studies have shown that post-discharge mortality can be closely linked to a variety
11 of key risk factors, such as malnutrition and disease severity [4–6]. Our results affirm this
12 through formal model development ~~These results confirm high post-discharge mortality rates, as~~
13 ~~previously demonstrated [4,5], but also indicate that those at highest risk can be identified~~ using
14 varied sets of few, objective, and easy-to-collect variables ~~that are~~ typically available in most
15 ~~austere~~ settings where such models would be used. In a model deployment context, however, the
16 general approach of developing a single model may not always be sufficient since missingness at
17 the point-of-care may be common. Having multiple simplified models with similar performance,
18 as we saw in our models, ~~s within each cohort~~ may help alleviate these kinds of logistical barriers
19 to implementation [28,29] ~~in different contexts~~. ~~We observed similar between-model~~
20 ~~performance (within each cohort) and minimal reduction in performance in the final models~~
21 ~~compared to the intermediary models, which would themselves be logistically challenging to~~
22 ~~implement. Collinearity within the large sets of predictors may account for this minimal~~
23 ~~performance loss.~~
1 Without an effective intervention, risk prediction has limited utility. Understanding discharge as
2 a dynamic process encompassing the time between admission and re-integration into community
3 care is integral to our focus on admission factors [30]. Early identification allows post-discharge
4 risk to be incorporated into discharge planning from the outset. Significant challenges in
5 preparing caregivers for discharge and the transition home have been identified, suggesting that
6 early planning is an essential component of effective peri-discharge care [30].
7 Choosing risk probability thresholds to classify post-discharge mortality as a binary outcome
8 depends on many factors, including availability of human resources, baseline risk, risk tolerance,
9 and impact on patients/caregivers. Though the thresholds chosen may prove useful in some
10 settings, choice of both the threshold and number of thresholds must be informed by local
11 context and constitutes a critically important consideration for deployment of this, or any, risk
12 model [31].
13 Although internal validation can justify using models within the region in which they are
14 derived, external validation using different data sources (ideally several) from different regions is
15 essential [32], using both existing and future data [5]. Consequently, we have several prospective
16 studies underway, and will establish data sharing agreements with other collaborators to enable
17 use of their collected data. However, not every conceivable implementation region for any given
18 model can be subjected to external validation. A more pragmatic approach is developing a
19 region-specific model-updating process, integrated over the life-course of the model. Calibration
20 drift due to secular trends, the measured impact of the model itself, and peculiarities of each
21 individual site are key considerations in model deployment [33]. Digitization of the healthcare
22 system will help establishing these processes [34].
1 As health systems in low-income countries increasingly adopt electronic health records,
2 incorporating algorithms to augment care decisions has tremendous potential to improve
3 outcomes and facilitate adoption of these digital systems [35,36]. Using routinely-collected
4 variables can allow models to run without additional user input and automatically prompt follow-
5 up guidance to the medical team and patient, encouraging adoption and linkage to interventional
6 programs. Furthermore, such systems can report baseline risk data and, when linked to follow-up
7 programs, data on readmission and mortality to national-level health management information
8 systems, such as DHIS2 [37]. These data can be used in model calibration and updating, ensuring
9 site-specific validity. Contextually-validated digital clinical decision support systems utilizing
10 risk algorithms are increasingly recognized as essential to achieving universal health coverage,
11 especially in low- and middle-income countries [38,39].
12 Limitations
13 This study has several limitations. While our models performed well with internal cross-
14 validation, demonstrating good performance in planned external validation is essential to
15 encourage adoption. Second, our models do not accommodate missing data for predictor
16 variables. While missing data rates were very low, this is unlikely to represent true rates of
17 missingness in real-world practice. We developed a family of models, varying in number and
18 type of predictors, which produced similar performance, to partially address this limitation.
19 Future research will explore more robust methods for addressing missing data, including
20 building sub-models to allow for every possible combination of missing variable [29]. Third,
21 these models were developed in the absence of a proven program to utilize a risk-based approach
22 to care, limiting their current utility. While merely knowledge of individual risk can change
23 behaviour and may influence provision of peri-discharge care, risk-informed approaches to
1 follow-up care are also currently under investigation [40]. Fourth, calibration was good at most
2 observed risk levels, but there were very few patients with predicted risk greater than 40-50%, so
3 calibration beyond these probabilities could not be assessed. Regardless, our models should
4 perform adequately for implementation purposes using the optimal threshold cut-offs identified.
5 Finally, the added value of these models may be questioned in the light of previously published
6 models [40–42]. Our models were based on purposively built cohorts, with *a priori* stakeholder
7 engagement regarding relevant variables and their measurement timing, and were uniquely
8 developed within the clinical rubric of suspected sepsis, which is increasingly recognized as a
9 global health priority.
10 Conclusion
11 Post-discharge mortality in the context of suspected sepsis occurs frequently in children under
12 five years old, but those at highest risk can be identified using simple clinical criteria, measured
13 at admission. Being able to select from a range of prediction models, with similar performance
14 parameters, may support wider implementation of digital risk-stratification tools in different
15 clinical settings. Future work must focus on both external validation as well evaluation of how
16 risk-stratified care can improve post-discharge outcomes~~Though future external validation is~~
17 ~~required, the use of these models within the context where they were collected can begin to~~
18 ~~transform discharge and post-discharge care~~.

19

## 1 Acknowledgements

2 We would like to acknowledge all past and present members of the Smart Discharges Research
3 program for their efforts in data collection, administration, logistics support, and all study
4 activities, including but not limited to: Tumwebaze Godfrey, Agaba Collins, Tumukunde Goreth,
5 Naturinda Mackline, Assimwe Abibu, Nakafero Joan, Kiiza Israel, Kitenda Julius, Kamba Ayub,
6 Kuguminkiriza Brenda, Kabajasi Olive, Kembabazi Brenda, Happy Annet, Tusingwire Fredson,
7 Nuwasasira Agaston, Ankatse Christine, Naturinda Rabecca, Nabawanuka Abbey Onyachi,
8 Kamazima Justine, Kairangwa Racheal, Ounyesiga Thomas, Mwoya Yuma, Twebaze Florence,
9 Bulage Mary, Tugumenawe Darius, Tuhame Dyonisius, Twesigye Leonidas, Kamusiime Olivia,
10 Ainembabazi Harriet, Abaho Samuel, Nakabiri Zaituni, Naigaga Shaminah, Kisame Zorah,
11 Babirye Clare, Kayegi Maliza, Opuko Wilson, Mwaka Savio, Baryahirwa Hassan, Mutungi
12 Alexander, Charlene Kanyali, Catherine Kiggundu, Alexia Krepiakevich, Brooklyn Nemetchek,
13 Jessica Trawin, Maryum Chaudhry, Peter Lewis, Rishika Bose, Sahar Zandi Nia, Tamara
14 Dudley, and Cherri Zhang. Without their effort and support, this study would not have been
15 possible.

16

## 17 Data sharing

18 Study materials including the study protocol, consent form, data collection tools, de-identified
19 participant data, data dictionary, and the analysis code are available on request to the
20 corresponding author (Matthew O. Wiens, [matthew.wiens@bcchr.ca](mailto:matthew.wiens@bcchr.ca)) or to the Institute for
21 Global Health at BC Children’s and Women’s Hospital (Jessica Trawin,
22 [jessica.trawin@cw.bc.ca](mailto:jessica.trawin@cw.bc.ca)) or through the published protocol
23 (<https://doi.org/10.5683/SP3/QRUMNQ>) and dataset (<https://doi.org/10.5683/SP3/REPMSY>).
1 Owing to the sensitive nature of clinical data, access to the de-identified data is granted on a
2 case-by-case basis and will require the signing of a data sharing agreement.

## References

1. Rudd KE, Johnson SC, Agesa KM, Shackelford KA, Tsoi D, Kievlan DR, et al. Global, regional, and national sepsis incidence and mortality, 1990–2017: analysis for the Global Burden of Disease Study. Lancet. 2020;395: 200–211. doi:10.1016/S0140- 6736(19)32989-7
2. Rudd KE, Kissoon N, Limmathurotsakul D, Bory S, Mutahunga B, Seymour CW, et al. The global burden of sepsis: barriers and potential solutions. Crit Care. 2018;22: 232. doi:10.1186/s13054-018-2157-z
3. Wiens MO, Kissoon N, Holsti L. Challenges in pediatric post-sepsis care in resource limited settings: a narrative review. Transl Pediatr. 2021;10: 2666–2677. doi:10.21037/tp- 20-390
4. Knappett M, Nguyen V, Chaudhry M, Trawin J, Kabakyenga J, Kumbakumba E, et al. Pediatric post-discharge mortality in resource-poor countries: a systematic review and meta-analysis. EClinicalMedicine. 2024;67: 102380. doi:10.1016/j.eclinm.2023.102380
5. Childhood Acute Illness and Nutrition (CHAIN) Network. Childhood mortality during and after acute illness in Africa and south Asia: a prospective cohort study. Lancet Glob Heal. 2022;10: e673–e684. doi:10.1016/S2214-109X(22)00118-8
6. Wiens MO, Bone JN, Kumbakumba E, Businge S, Tagoola A, Sherine SO, et al. Mortality after hospital discharge among children younger than 5 years admitted with suspected sepsis in Uganda: a prospective, multisite, observational cohort study. Lancet Child Adolesc Heal. 2023;7: 555–566. doi:10.1016/S2352-4642(23)00052-4
7. Nemetchek B, English L, Kissoon N, Ansermino JM, Moschovis PP, Kabakyenga J, et al. Paediatric postdischarge mortality in developing countries: a systematic review. BMJ Open. 2018;8: e023445. doi:10.1136/bmjopen-2018-023445
8. Paul S, Tickell KD, Ojee E, Oduol C, Martin S, Singa B, et al. Knowledge, attitudes, and perceptions of Kenyan healthcare workers regarding pediatric discharge from hospital. Prazeres F, editor. PLoS One. 2021;16: e0249569. doi:10.1371/journal.pone.0249569
9. Krepiakevich A, Khowaja AR, Kabajaasi O, Nemetchek B, Ansermino JM, Kissoon N, et al. Out of pocket costs and time/productivity losses for pediatric sepsis in Uganda: a mixed-methods study. BMC Health Serv Res. 2021;21: 1252. doi:10.1186/s12913-021- 07272-9
10. Nemetchek B, Khowaja A, Kavuma A, Kabajaasi O, Olirus Owilli A, Ansermino JM, et al. Exploring healthcare providers’ perspectives of the paediatric discharge process in Uganda: a qualitative exploratory study. BMJ Open. 2019;9: e029526. doi:10.1136/bmjopen-2019-029526
11. English L, Kumbakumba E, Larson CP, Kabakyenga J, Singer J, Kissoon N, et al. Pediatric out-of-hospital deaths following hospital discharge: a mixed-methods study. Afr Health Sci. 2016;16: 883–891. doi:10.4314/ahs.v16i4.2
12. Ahmed SM, Brintz BJ, Talbert A, Ngari M, Pavlinac PB, Platts-Mills JA, et al. Derivation and external validation of a clinical prognostic model identifying children at risk of death following presentation for diarrheal care. PLOS Glob public Heal. 2023;3: e0001937. doi:10.1371/journal.pgph.0001937
13. Wiens MO, Kumbakumba E, Larson CP, Ansermino JM, Singer J, Kissoon N, et al. Postdischarge mortality in children with acute infectious diseases: derivation of

postdischarge mortality prediction models. BMJ Open. 2015;5: e009449. doi:10.1136/bmjopen-2015-009449

14. Collins GS, Reitsma JB, Altman DG, Moons KGM. Transparent Reporting of a multivariable prediction model for Individual Prognosis Or Diagnosis (TRIPOD): The TRIPOD Statement. Ann Intern Med. 2015;162: 55–63. doi:10.7326/M14-0697
15. Uganda Bureau of Statistics. Population & Censuses. 2022 [cited 5 Dec 2023]. Available: https:/[/www.ubos.org/explore](http://www.ubos.org/explore-statistics/20/)-[statistics/20/](http://www.ubos.org/explore-statistics/20/)
16. Goldstein B, Giroir B, Randolph A, International Consensus Conference on Pediatric Sepsis. International pediatric sepsis consensus conference: definitions for sepsis and organ dysfunction in pediatrics. Pediatr Crit Care Med. 2005;6: 2–8. doi:10.1097/01.PCC.0000149131.72248.E6
17. Wiens M, Kissoon N (Tex), Ansermino JM, Barigye C, Businge S, Kumbakumba E, et al. Smart Discharges to improve post-discharge health outcomes in children: A prospective before-after study with staggered implementation. In: Borealis, V1 [Internet]. 2023 [cited 5 Dec 2023]. Available: https://doi.org/10.5683/SP3/QRUMNQ
18. Wiens MO, Kissoon N, Kumbakumba E, Singer J, Moschovis PP, Ansermino JM, et al. Selecting candidate predictor variables for the modelling of post-discharge mortality from sepsis: a protocol development project. Afr Health Sci. 2016;16: 162–9. doi:10.4314/ahs.v16i1.22
19. Nemetchek BR, Liang L, Kissoon N, Ansermino MJ, Kabakyenga J, Lavoie PM, et al. Predictor variables for post-discharge mortality modelling in infants: a protocol development project. Afr Health Sci. 2018;18: 1214–1225. doi:10.4314/ahs.v18i4.43
20. Harris PA, Taylor R, Thielke R, Payne J, Gonzalez N, Conde JG. Research electronic data capture (REDCap)--a metadata-driven methodology and workflow process for providing translational research informatics support. J Biomed Inform. 2009;42: 377–81. doi:10.1016/j.jbi.2008.08.010
21. Harris PA, Taylor R, Minor BL, Elliott V, Fernandez M, O’Neal L, et al. The REDCap consortium: Building an international community of software platform partners. J Biomed Inform. 2019;95: 103208. doi:10.1016/j.jbi.2019.103208
22. Jeni LA, Cohn JF, De La Torre F. Facing Imbalanced Data Recommendations for the Use of Performance Metrics. Int Conf Affect Comput Intell Interact Work [proceedings] ACII. 2013;2013: 245–251. doi:10.1109/ACII.2013.47
23. Kuhn M. Building Predictive Models in R Using the caret Package. J Stat Softw. 2008;28. doi:10.18637/jss.v028.i05
24. R Core Team. R: A language and environment for statistical computing. In: R Founcation for Statistical Computing, Vienna, Austria [Internet]. 2021 [cited 5 Dec 2023]. Available: https:/[/www.r](http://www.r-project.org/)-[project.org/](http://www.r-project.org/)
25. Wiens MO, Kumbakumba E, Larson CP, Moschovis PP, Barigye C, Kabakyenga J, et al. Scheduled Follow-Up Referrals and Simple Prevention Kits Including Counseling to Improve Post-Discharge Outcomes Among Children in Uganda: A Proof-of-Concept Study. Glob Heal Sci Pract. 2016;4: 422–434. doi:10.9745/GHSP-D-16-00069
26. Akech S, Kwambai T, Wiens MO, Chandna A, Berkley JA, Snow RW. Tackling post- discharge mortality in children living in LMICs to reduce child deaths. Lancet Child Adolesc Heal. 2023;7: 149–151. doi:10.1016/S2352-4642(22)00375-3
27. Wiens MO, Kissoon N, Kabakyenga J. Smart Hospital Discharges to Address a Neglected Epidemic in Sepsis in Low- and Middle-Income Countries. JAMA Pediatr. 2018;172: 213–214. doi:10.1001/jamapediatrics.2017.4519
28. Janssen KJM, Vergouwe Y, Donders ART, Harrell FE, Chen Q, Grobbee DE, et al. Dealing with missing predictor values when applying clinical prediction models. Clin Chem. 2009;55: 994–1001. doi:10.1373/clinchem.2008.115345
29. Hoogland J, van Barreveld M, Debray TPA, Reitsma JB, Verstraelen TE, Dijkgraaf MGW, et al. Handling missing predictor values when validating and applying a prediction model to new patients. Stat Med. 2020;39: 3591–3607. doi:10.1002/sim.8682
30. Kabajaasi O, Trawin J, Derksen B, Komugisha C, Mwaka S, Waiswa P, et al. Transitions from hospital to home: A mixed methods study to evaluate pediatric discharges in Uganda. PLOS Glob public Heal. 2023;3: e0002173. doi:10.1371/journal.pgph.0002173
31. Wynants L, van Smeden M, McLernon DJ, Timmerman D, Steyerberg EW, Van Calster

B. Three myths about risk thresholds for prediction models. BMC Med. 2019;17: 192. doi:10.1186/s12916-019-1425-3

32. Ramspek CL, Jager KJ, Dekker FW, Zoccali C, van Diepen M. External validation of prognostic models: what, why, how, when and where? Clin Kidney J. 2020;14: 49–58. doi:10.1093/ckj/sfaa188
33. Otokiti AU, Ozoude MM, Williams KS, Sadiq-onilenla RA, Ojo SA, Wasarme LB, et al. The Need to Prioritize Model-Updating Processes in Clinical Artificial Intelligence (AI) Models: Protocol for a Scoping Review. JMIR Res Protoc. 2023;12: e37685. doi:10.2196/37685
34. Davis SE, Greevy RA, Lasko TA, Walsh CG, Matheny ME. Detection of calibration drift in clinical prediction models to inform model updating. J Biomed Inform. 2020;112: 103611. doi:10.1016/j.jbi.2020.103611
35. Mitchell M, Kan L. Digital Technology and the Future of Health Systems. Heal Syst Reform. 2019;5: 113–120. doi:10.1080/23288604.2019.1583040
36. Sharma V, Ali I, van der Veer S, Martin G, Ainsworth J, Augustine T. Adoption of clinical risk prediction tools is limited by a lack of integration with electronic health records. BMJ Heal Care Informatics. 2021;28: e100253. doi:10.1136/bmjhci-2020-100253
37. University of Oslo. The world’s largest health information management system — developed through global collaboration led by UiO. In: dhis2 [Internet]. [cited 5 Dec 2023]. Available: https://dhis2.org/
38. Endalamaw A, Erku D, Khatri RB, Nigatu F, Wolka E, Zewdie A, et al. Successes, weaknesses, and recommendations to strengthen primary health care: a scoping review. Arch Public Health. 2023;81: 100. doi:10.1186/s13690-023-01116-0
39. Manyazewal T, Woldeamanuel Y, Blumberg HM, Fekadu A, Marconi VC. The potential use of digital health technologies in the African context: a systematic review of evidence from Ethiopia. NPJ Digit Med. 2021;4: 125. doi:10.1038/s41746-021-00487-4
40. ClinicalTrials.gov. Smart Discharges to Improve Post-discharge Health Outcomes in Children. In: NCT05730452 [Internet]. 2023 [cited 5 Dec 2023]. Available: https://clinicaltrials.gov/ct2/show/NCT05730452
41. Madrid L, Casellas A, Sacoor C, Quintó L, Sitoe A, Varo R, et al. Postdischarge Mortality Prediction in Sub-Saharan Africa. Pediatrics. 2019;143: e20180606.

doi:10.1542/peds.2018-0606

42. Ngari MM, Fegan G, Mwangome MK, Ngama MJ, Mturi N, Scott JAG, et al. Mortality after Inpatient Treatment for Severe Pneumonia in Children: a Cohort Study. Paediatr Perinat Epidemiol. 2017;31: 233–242. doi:10.1111/ppe.12348

**Figure Legends**

#### Figure 1. Variable selection for model development

**Figure 2. Study enrolment flow diagram**

**Figure 3. Performance of the final clinical model for 0-6 months (M6PD-C0-6) on the full dataset**. The points on the receiver operating characteristic (ROC), precision recall (PR), and gain curve plots indicate co-ordinates for the probability threshold at sensitivity=80%, with positive predictive value (PPV) and negative predictive value (NPV) also reported at this threshold.

**Figure 4. Performance of the final clinical model for 6-60 months (M6PD-C6-60) on the full dataset.** The points on the receiver operating characteristic (ROC), precision recall (PR), and gain curve plots indicate co-ordinates for the probability threshold at sensitivity=80%, with positive predictive value (PPV) and negative predictive value (NPV) also reported at this threshold.

**Online supplementary material.** S1) Details of study cohorts and variables used for the full and intermediary models; S2) Statistical Methods and Analyses; S3) Intermediary Clinical Variable Models – Variable Importance; S4) Intermediary Clinical and Social Variable Models – Variable Importance; S5) Intermediary Any Variable Models – Performance Metrics, Coefficients and Variable Importance; S6) Final Clinical Variable Models, M6PD-C0-6 and M6PD-C6-60 – Performance Metrics and Coefficients; S7) Final Clinical and Social Variable Models, M6PD- CS0-6 and M6PD-CS6-60 – Performance Metrics and Coefficients; S8) Final Any Variable Models, M6PD-A0-6 and M6PD-A6-60 – Performance Metrics and Coefficients; S9) Literature review.

Response to Reviewers

2^nd^ March 2024

Dear Dr Collins Otieno Asweto,

Re: **PGPH-D-24-00205**, Prediction models for post-discharge mortality among under-five children with suspected sepsis in Uganda: A multicohort analysis, PLOS Global Public Health

Please thank Dr Botha and Dr Likaka for reviewing our manuscript and for their thoughtful comments and helpful suggestions. We address each of their points below along with your journal requirements. Each point (journal requirement or reviewer’s comment) is presented on a light grey background; our response is underneath, with any revisions to the text marked in italics.

Some of these comments have prompted modifications to the manuscript text. A revised version has been submitted, with tracked changes along with a final version, as requested.

We hope that we have addressed the reviewers’ questions adequately and that you find our proposed revisions to the manuscript acceptable.

Sincerely,

**Dr Matthew O Wiens**, PharmD, PhD

Assistant Professor | Department of Anesthesiology, Pharmacology & Therapeutics | Institute for Global Health The University of British Columbia | BC Children's Hospital

938 W 28th Ave | Vancouver BC | V5Z 4H4 Canada, Phone 604 997 7753, [matthew.wiens@bcchr.ca](mailto:matthew.wiens@bcchr.ca)

**Dr Jerome Kabakyenga**, MBChB, PhD

Professor | Maternal Newborn & Child Health Institute | Mbarara University of Science and Technology

P.O. Box 1410 | Mbarara, Uganda. Phone +256 77 259 0409 [jkabakyenga@must.ac.ug](mailto:jkabakyenga@must.ac.ug)

#### Journal Requirements

1. Please review your reference list to ensure that it is complete and correct. If you have cited papers that have been retracted, please include the rationale for doing so in the manuscript text, or remove these references and replace them with relevant current references. Any changes to the reference list should be mentioned in the rebuttal letter that accompanies your revised manuscript. If you need to cite a retracted article, indicate the article’s retracted status in the References list and also include a citation and full reference for the retraction notice.

We have checked each of our references to ensure they are complete and correct. We amended minor citation details for existing references and added two additional references:

- Knappett et al. Pediatric post-discharge mortality in resource-poor countries: a systematic review and meta- analysis. EClinicalMedicine. 2024;67: 102380. doi:10.1016/j.eclinm.2023.102380 (addressing reviewer 1 comment on our Introduction)
- Janssen et al. Dealing with missing predictor values when applying clinical prediction models. Clin Chem. 2009;55: 994–1001. doi:10.1373/clinchem.2008.115345 (addressing reviewer 1 comment on our Discussion)
- Hoogland et al. Handling missing predictor values when validating and applying a prediction model to new patients. Stat Med. 2020;39: 3591–3607. doi:10.1002/sim.8682 (again, addressing reviewer 1 comment on our Discussion)

2. Please note that PLOS GPH has specific guidelines on code sharing for submissions in which author- generated code underpins the findings in the manuscript. In these cases, all author-generated code must be made available without restrictions upon publication of the work. Please review our guidelines at <https://journals.plos.org/globalpublichealth/s/materials-and-software-sharing#loc-sharing-code> and ensure that your code is shared in a way that follows best practice and facilitates reproducibility and reuse.

We have identified the code that we will release. It will be available openly, as described in our data sharing statement on p23 of the manuscript.

3. Please provide separate figure files in .tif or .eps format only and remove any figures embedded in your manuscript file. Please also ensure all files are under our size limit of 10MB.

For more information about figure files please see our guidelines:

<https://journals.plos.org/globalpublichealth/s/figures> <https://journals.plos.org/globalpublichealth/s/figures#loc-file-requirement>

We have provided all figures as .eps files. We noticed a small correction was required in the x-axis of one of the component graphs in figure 3. In correcting this, we have decided to separate the two halves of figure 3 into separate figures (now figure 3 and figure 4) as each is already a multi-component figure.

We have renumbered figure citations in the manuscript and provided the updated legends.

4. We have noticed that you have uploaded Supporting Information files, but you have not included a list of legends. Please add a full list of legends for your Supporting Information files after the references list.

We apologize for missing this in our original submission. We have added a list of legends after the references – for our four figures (see above, we have split our original figure 3 into separate figure 3 and figure 4) and our one supporting information file, which includes a detailed contents list on its first two pages.

#### Reviewers' comments

Reviewer #1: BOTHA, Nkosi Nkosi

On the whole, the study was very well executed and presented. The authors have demonstrated a very knowledge of the subject matter and language. I commend the authors for a very good job done and suggest that they address the few remarks provided to improve the overall quality of the manuscript. The main issue is with the discussion.

Thank you for these comments. We address each of your separate points below.

**TOPIC**

Prediction models for post-discharge mortality among under-five children with suspected sepsis in Uganda: A multicohort analysis

**Remark(s) 1:** Reads fine.

Thank you.

**ABSTRACT**

**Introduction:** “Identifying those at risk has limited progress to improve outcomes.”

**Remark(s) 2, page 2, line 3:** This sentence does not read well. Recast, please.

**Methods:** Adequate.

**Results:** Adequate.

**Conclusion:** Adequate.

We have re-written this sentence:

*“The lack of available tools to identify those at risk of post-discharge mortality has limited the ability to make progress towards improving outcomes.”*

**INTRODUCTION**

**Page 3, para 2, lines 9 & 10:** “Robust epidemiological data for pediatric post-discharge mortality in the context of sepsis and severe infection have been limited.”

**Remark(s) 3:** Provide reference for this statement.

We are now able to cite our recently published meta-analysis, which includes all post-discharge research including the few sepsis focused studies, and have added this reference to the manuscript:

- Knappett et al. Pediatric post-discharge mortality in resource-poor countries: a systematic review and meta- analysis. EClinicalMedicine. 2023;67:102380. doi: 10.1016/j.eclinm.2023.102380.

**MATERIALS AND METHODS**

**Protocol**

Detailed and rigorous

**RESULTS**

Adequately presented

Thank you.

**DISCUSSION**

**Remark(s):** While I credit the authors for a thorough presentation of this session, the session must be re- written completely. Typically, discussions are expected to highlight the main findings of the current study (not a mere repeat of the results) and show evidence of how these findings affirm or disaffirm existing evidence or previous studies. This way, readers can clearly relate the findings of the current study to existing evidence or previous studies and make better sense of the study. As it stands, the discussion looks more like the ‘results’. I further suggest that, if possible, the discussion be organised under themes for ease of understanding by readers.

Thank you for your careful review of our paper and your comments specifically related to the discussion. We agree that parts of the discussion were perhaps too focused on repeating some of our results. In particular, the 2^nd^ paragraph seems to inappropriately repeat some of our key findings. We have re- written this paragraph completely, removing several sentences and re-writing the remaining parts to focus specifically on the issue of (1) how our results fit into the existing literature on post-discharge risk and (2) how model deployment considerations, such as missingness of parameters is critically important for adoption and reach, and thus how having multiple models helps assuage such concerns. See p19 of the revised manuscript.

With regards to the remaining paragraphs, we do not feel we have re-stated our results.

- The 3^rd^ paragraph focuses on how prediction alone is insufficient to improve outcomes and we cite research to this effect. Of note, no studies have been conducted to address post-discharge mortality in the context of sepsis, thus we have no other interventional literature to draw on.
- The 4^th^ paragraph highlights the perennial issue of choosing model thresholds. What defines high vs low risk is context and resources. This issue is rarely addressed in the modelling literature, and we

feel it’s critical to include such a discussion given the context where these kinds of models might be deployed.

- The 5^th^ paragraph relates to validation and the gradient that defines external vs internal validation. This is key given most models are never validated.
- The 6^th^ paragraph relates to digital health strategies within low-income country settings. Increasingly countries are implementing digital systems to facilitate patient care in both facility and community settings. As nations adopt such strategies, its importance (and potential) as it pertains to prediction cannot be understated.

These paragraphs form our discussion, and we feel they are quite relevant to the topic of prediction models in child health. We would welcome further discussion on this and are keen to do any further revisions on our discussion as it is deemed necessary by the editorial and review team.

**CONCLUSION**

**Remark(s):** Authors should make provision for contribution to knowledge and practice and also propose some clear recommendations for further research.

Thank you for this comment. We have re-written the conclusion highlighting the contribution to knowledge (development of models, reporting of post-discharge outcomes) and a call to further research (external validation, interventional research). See p22 of the revised manuscript.

**OVERALL COMMENTS**

On the whole, the study was very well executed and presented. The authors have demonstrated a very knowledge of the subject matter and language. I commend the authors for a very good job done and suggest that they address the few remarks provided to improve the overall quality of the manuscript. The main issue is with the discussion. The authors need to reorganise this session so as the readers can make a better sense of the study.

Thank you for the positive comments. See our response above to your comment about our Discussion.

Reviewer #2: Dr Andrew Likaka

The study has been rigorous, following all research standards. The statistics are well thoughtful and easy to apply. Great findings too. Relevance to the study setting and the results can be generalized due to the methodology used.

Thank you for these positive comments.
